# Distinct gut and oral microbiome patterns associated with dyslexia in a family-based cohort: A preliminary exploratory study

**DOI:** 10.1371/journal.pone.0353463

**Published:** 2026-07-21

**Authors:** Sterling L. Wright, Mia Joslin, Yookyung Kim, Magdalena Olson, Ayden Hall, Beate Peter, Corrie M. Whisner

**Affiliations:** 1 College of Health Solutions, Arizona State University, Phoenix, Arizona, United States of America; 2 Center for Health Through Microbiomes, The Biodesign Institute, Arizona State University, Tempe, Arizona, United States of America; 3 School of Human Evolution and Social Change, Arizona State University, Tempe, Arizona, United States of America; 4 School of Life Sciences, The College of the Liberal Arts and Sciences, Arizona State University, Tempe, Arizona, United States of America; Suez Canal University Faculty of Medicine, EGYPT

## Abstract

**Background:**

Many neurodevelopmental disorders, including dyslexia and childhood apraxia of speech (CAS), have genetic predispositions that are understood to varying degrees. However, the microbiome in individuals with dyslexia and CAS remains underexplored. The goal of this exploratory study was to determine whether fecal and saliva microbiome diversity and composition are associated with dyslexia or CAS.

**Methods:**

To this end, we examined the fecal and saliva microbiota of individuals with dyslexia, CAS, and their neurotypical family members using 16S rRNA gene amplicon sequencing in a family-based cohort composing of 7 individuals with dyslexia, 11 with CAS, and 10 neurotypical family members (n = 28). Participants with dyslexia and CAS were drawn from separate families, with neurotypical relatives serving as within-family controls. A total of 19 fecal and 29 saliva samples were collected, with paired fecal-saliva samples available for 19 individuals. Taxonomic classification was performed using four 16S rRNA reference databases, and microbial diversity, composition, and functional potential were analyzed.

**Results:**

Individuals with dyslexia consistently showed distinct fecal microbiome alpha and beta diversity patterns at the species level compared to neurotypical family members and participants with CAS histories, irrespective of taxonomic database employed. Both fecal and saliva datasets identified key taxa associated with dyslexia, but not with CAS. Predicted functional profiling further identified dyslexia-associated pathways in the fecal microbiome, whereas no functional differences were detected in saliva.

**Conclusion:**

Although these results suggest that individuals with dyslexia may harbor distinct fecal and saliva microbiomes, the findings are exploratory and should be considered as hypothesis-generating. Future studies leveraging larger, independent cohorts will be essential to validate these findings and to more rigorously examine the oral-gut-brain axis in language-based syndromes.

## Introduction

Many neurodevelopmental disorders (NDDs) affect cognitive, behavioral, and communication functioning, which in turn can impact an individual’s personal, social, academic, and occupational performance. NDDs encompass a broad range of phenotypes, defined here as clinically assessed speech and language statuses, including childhood apraxia of speech (CAS) and dyslexia [1,2]. CAS is a severe disorder of speech sound production characterized by small consonant inventories, vowel errors, and inconsistent word productions thought to arise from motor discoordination [3–5]. Dyslexia, on the other hand, is a learning disability primarily associated with written language skills [6,7]. Although each is distinct in their core observable characteristics (*i.e.,* phenotypes) and clinical management, both are frequently comorbid [8] and share some biomarkers for motor discoordination and difficulties with sequential information processing [9].

Over the past two decades, significant progress has been made in identifying genetic factors linked to CAS and dyslexia. Several candidate genes, such as *FOXP2, SETBP1, SETD1A, DDX3X,* and *BCL11A* are among genes that have been implicated for CAS [10–16], whereas *ROBO1, DCDC2, KIAA0319,* and *DYXC1,* have been associated with dyslexia [6,17–20]. These genes influence neural pathways involved in auditory processing, working memory, and motor planning for speech. However, no single gene accounts for the full range of phenotypic variation observed in either CAS or dyslexia, highlighting the complexity and polygenic nature of these disorders and suggesting an important role of gene-environment interactions.

CAS and dyslexia are related but separable language-based neurodevelopmental disorders that affect different components of speech and language processing. Reading and phonological decoding are involved with dyslexia, while speech and motor planning in CAS. Yet, they may share overlapping neurobiological vulnerabilities. This makes them a useful comparative framework for exploring biological factors that may be shared across language impairments versus those that are disorder specific. One such factor may be the microbiome, as emerging evidence indicates that microbial communities involved along the oral-gut-brain axis can influence neurodevelopment, cognition, and behavior through immune, metabolic, and neuroactive signaling pathways [21–23]. These pathways include microbial metabolites, immune signaling, and neural routes, all of which have been implicated in modulating brain function and behavior [24–27]. For example, microbial pathways related to γ-aminobutyric acid (GABA) production and sulfur metabolism have been implicated in neurodevelopmental and neuropsychiatric conditions [28–30].

To date, however, most studies have examined either the gut-brain axis or the oral-brain axis in isolation, with relatively few exploring these systems in conjunction [23]. Numerous studies have identified gut microbiome differences associated with autism spectrum disorder (ASD) [31–34], including variation in microbial diversity, community composition, and metabolic activity when compared to unaffected, *i.e.,* neurotypical, controls [35–37]. In addition, the oral microbiome may also be implicated in both gut and brain health [38,39]. Within this context, variation in oral and gut microbial communities may act as mediators that interact with underlying genetic susceptibility, potentially contributing to the heterogeneity of speech and language outcomes observed in individuals living with CAS and dyslexia.

Despite the growing body of literature linking the gut and oral microbiome to NDDs [40,41], potential microbiome associations with CAS or dyslexia remain largely unexplored. We address this gap by examining the fecal and saliva microbiomes of individuals diagnosed with dyslexia or CAS, and their neurotypical family members. Stool and saliva samples were used as proxies for the gut and oral microbiota, respectively. Throughout the manuscript, we refer to these datasets specifically as the fecal microbiome and salivary microbiome, while the terms gut microbiome and oral microbiome are used more broadly in conceptual discussions. By integrating microbiome data with neurodevelopmental assessments, our exploratory study provides an initial step toward identifying microbial patterns potentially linked to CAS and dyslexia, offering a novel lens on how the oral-gut-brain axis interacts with neurodevelopmental pathways.

## Materials and methods

### Recruitment and sample collection

This study was approved by the Institutional Review Board of the Arizona State University (ASU) in accordance with the Code of Ethics of the World Medical Association (IRB: STUDY00013638 “Biology of Language”). Participants were originally enrolled at the University of Washington (IRB # 34416). Written informed consent was obtained from all adult participants, and parental consent was provided for all participants who were minors. The recruitment period took place from 07/19/2021–07/18/2022.

### Participant information

**Fig 1:**
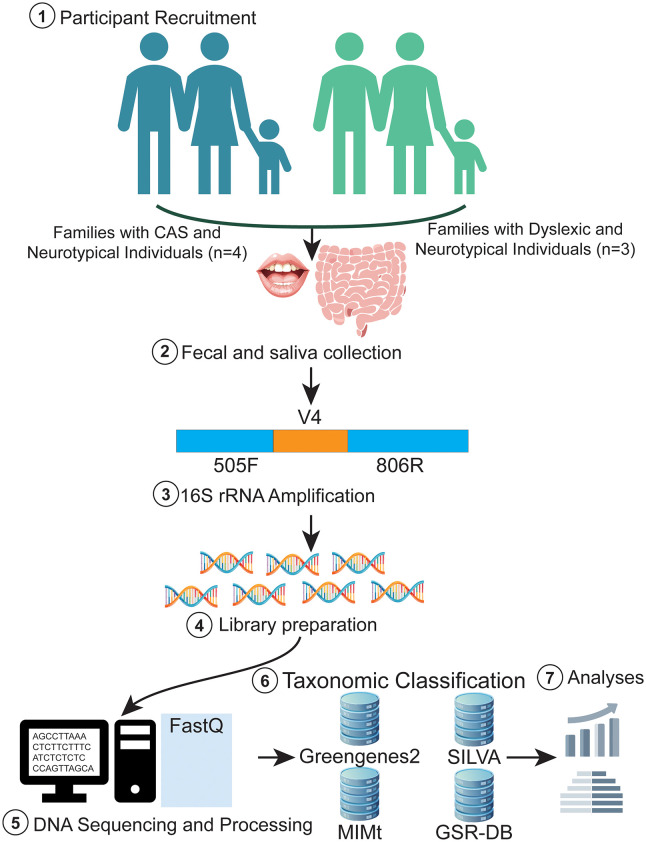
Schematic overview of the study design and analytical workflow. Families with childhood apraxia of speech (CAS) and dyslexia, along with neurotypical family members, were recruited for participation. Fecal and saliva samples were collected from all participants, followed by 16S rRNA gene amplification targeting the V4 region. Amplicon libraries were prepared for Illumina sequencing and sequenced on a MiSeq platform using 2 × 250 bp paired-end chemistry. Sequence data were processed using QIIME2, and taxonomic classification was performed using multiple reference databases (Greengenes2, SILVA, MIMt, and GSR-DB) with the classify-sklearn method. Downstream analyses were conducted at the genus and species levels and included alpha diversity, beta diversity, differential abundance testing, and functional inference using PICRUSt.

A total of 29 individuals participated in the study (Fig 1; Table 1). Participants were characterized as ‘Typical’ (neurotypical), ‘Apraxia of Speech’ (CAS), or ‘Dyslexia’ based on standardized speech and language assessments (S1 Appendix). One participant could not be definitively classified due to inconclusive assessment results and was therefore categorized as having an undetermined phenotype (*i.e.,* Unknown).

**Table 1:** Demographic and clinical characteristics of the study cohort.

Cohort	Diagnosis	# of individuals	Sex	Age Mean (SD)
Washington	Dyslexia	7	M = 1; F = 6	42.14 (17.53)
	Control: unaffected family members	1	F = 1	59
Arizona	CAS	11	M = 7; F = 4	30.33 (19.33)
	Control: unaffected family members	9	M = 5; F = 4	29.13 (10.00)
	Unknown	1	F = 1	41

This table summarizes the geographic distribution (Washington and Arizona), diagnostic categories, number of individuals per group, sex distribution, and mean age.

Participants were classified as having CAS history if they had (1) a self-reported history of CAS or severe childhood speech difficulties and (2) scored below −1 standard deviation (SD) on at least one of three multisyllabic diadochokinetic (DDK) tasks (/pata/, /taka/, /pataka/), which have been shown to reveal residual speech motor deficits in individuals with a history of CAS [9]. When DDK testing was not feasible, CAS status was determined based on family history and responses to a questionnaire regarding prior speech therapy. Participants in the dyslexia group were required to have a professional diagnosis of dyslexia and score below −1 SD on at least one of five standardized tests of written language ability: the Word Identification and Word Attack subtests from the Woodcock Reading Mastery Test–Revised (WRMT-III) [42], the Sight Word Reading Efficiency and the Phonemic Decoding Efficiency subtests from the Test of Word Reading Efficiency—Second Edition (TOWRE-2) [43], or the spelling subtest from the Wechsler Individual Achievement Test–Third Edition (WIAT-III) [44]. Additional details about these tests can be found in the S1 Appendix.

The CAS group comprised four families with familial CAS. The first CAS family consisted of a mother and two offspring, all affected, and an unaffected father, all of whom provided saliva samples only. The second CAS family consisted of an unaffected mother and six affected offspring; six of the seven members provided both fecal and saliva samples and one member provided a saliva sample only. The third CAS family consisted of two unaffected parents, one affected offspring and one unaffected offspring, and all provided saliva samples only. The fourth family consisted of an unaffected mother, three affected offspring, the mother’s sister, and that sister’s offspring, all of whom provided both types of samples. In total, samples were collected from 13 participants with CAS and 6 neurotypical family members. Across these families, twelve participants provided both sample types, while nine provided saliva samples only.

The dyslexia group comprised three families with familial dyslexia. The first family included an unaffected mother and one affected offspring, both of whom provided fecal and saliva samples. The second family consisted of an unaffected mother, an affected father, and an affected offspring; all three provided both sample types. The third family included an affected mother and two affected offspring; two provided both fecal and saliva samples, while one provided a saliva sample only. In total, samples were collected from six participants with dyslexia and two neurotypical family members. Across these families, seven participants provided both fecal and saliva samples, and one participant provided a saliva sample only.

In total, 29 saliva samples and 19 fecal samples were collected (S1 Table in S1 File). The difference in sample numbers reflect participant choosing to provide either both sample types or saliva only. For all 19 fecal samples, a matched saliva sample from the same participant was also available.

### 16S rRNA gene sequencing

Fecal and saliva samples were collected and placed in Gut (OMR-200) kits (DNA Genotek Inc., Ontario, Canada) and OMNIgene® Oral (OME-505) kits, respectively (Fig 1B). DNA was extracted using the DNeasy PowerSoil Kit (QIAGEN, Hilden, Germany) according to the directions from the manufacturer. The V4 region of the 16S rRNA gene was amplified using the barcoded primer set 515f/806r [45], following the Earth Microbiome Project (EMP) (http://www.earthmicrobiome.org/emp-standard-protocols/). PCR reactions were performed in duplicates, pooled, and quantified using the AccuBlue® dsDNA Quantitation Kit (Biotium, Fremont, CA, USA). For library preparation, 240 ng of pooled DNA per sample was combined, purified with the QIA quick PCR purification kit (QIAGEN, Hilden, Germany), and quantified on a Qubit Fluorometer (Thermo Fisher Scientific, Waltham, MA, USA). The final library was diluted to 4 nM, denatured, and further diluted to 4 pM with 25% of PhiX. Finally, sequencing was performed on an Illumina MiSeq platform at the Arizona State University Genomics Core using the 2 x 250 bp paired-end version 2 chemistry.

No extraction blanks or PCR-negative controls were included during DNA extraction amplification, or library preparation. As a result, formal contaminant identification methods based on negative controls (*e.g.,* frequency- or prevalence-based filtering) could not be applied.

### Sequence data processing

Amplicon data was bioinformatically processed on the Sol Supercomputer at Arizona State University [46]. The raw amplicon sequencing reads were initially processed with AdapterRemoval2 to remove adapter sequences and low-quality bases. Reads were filtered using the following parameters: --min-length 30, --trimqualities, --trimns, --minquality 20, ensuring the removal of low-quality bases and ambiguous nucleotides while retaining reads of sufficient quality and length for downstream analyses. Basic sequencing statistics, including read count, average read length, GC content of reads per sample, and quality metrics, were calculated using SeqKit (v.2.9.0) with the seqkit stats -a command.

Adapter-trimmed reads were then imported into QIIME2 (v.2024.5) [47,48] for further processing. Paired-end reads were denoised using the DADA2 plugin (*qiime dada2 denoise-pair*) with the following truncation parameters: --p-trunc-len-f 250 and –p-trunc-len-r 250. This step removed low-quality trailing regions and generated a feature table and representative sequences.

### Taxonomic classification

For many years, the SILVA database has served as a foundational resource for 16S rRNA gene-based microbiome studies. Its widespread use was largely due to its status as one of the largest and most comprehensive 16S databases available, providing extensive coverage of aligned, quality-checked small subunit (SSU) rRNA gene sequences [49,50]. However, the landscape of 16S reference databases has expanded significantly in recent years. The introduction of updated resources such as Greengenes2 [51], along with the emergence of newer databases like GSR-DB [52] and MIMt [53], highlights the value of incorporating and validating multiple reference frameworks. These newer databases offer alternative taxonomic curation strategies and provide opportunities to assess the consistency of taxonomic assignments across varying database structures. For instance, these databases differ in content (some prioritize specific regions (V3 or V4), while others rely on the full-length of the 16S rRNA gene), number of reference sequences, curation methods, and how often they are updated [54].

To address the potential for reference database bias, we aligned our V4 region sequences against multiple databases: Greengenes2 (v.2024.09) [51], SILVA (version 138, 99%) [55], MIMt2.0 [53], and GSR-DB (full-length 16S database) [52]. Because each database includes the V4 region, this approach allowed us to identify discrepancies in taxonomic classification across databases and ensure that observed trends were not dependent on a single reference framework. The results reported in the main text are derived from the Greengenes2 dataset. Benchmarking studies have demonstrated that Greengenes2 offers highly accurate and consistent classifications, with notably low false positive and false negative rates in both human fecal and oral microbiome datasets [51,56,57]. For example, Nagai et al. (2024) reported that V4-derived sequences aligned to Greengenes2 showed the strongest agreement with theoretical species compositions compared to SILVA and the Human Oral Microbiome Database (HOMD), suggesting it minimizes taxonomic assignment bias [57].

Taxonomic classification of representative sequences was performed using the QIIME2 *feature-classifier* plugin with the *classify-sklearn* method. For each reference database, a pre-trained Naive Bayes classifier was used to generate corresponding feature tables. Reference sequences were not trimmed to the exact V4 amplicon region prior to classifier training. Each of the four feature tables were collapsed to the genus (*i.e.,* --p-level 6) and species level (*i.e., --*p-level 7) using the *qiime taxa collapse* command. These collapsed tables were used in subsequent taxon-based statistical and diversity analyses.

To reduce technical noise and minimize the influence of rare taxa that may represent potential contaminants, features were filtered prior to downstream analyses. Specifically, features observed in fewer than two samples and with a total frequency less than 10 reads across the entire dataset were removed. This filtering step reduced the contribution of extremely low-abundance features that are more susceptible to stochastic and contamination-related bias, while retaining taxa consistently detected across individuals.

### Relative abundance analysis

To assess microbial composition, relative abundance data were analyzed separately for fecal and saliva microbiome samples. The dataset was divided into two subsets: one containing only fecal samples and the other containing only saliva samples. Within each subset, microbial species that had at least 5% relative abundance in a minimum of two samples were retained for visualization. Species that did not meet this criterion were grouped into a composite category labeled “Other.” The resulting data were plotted as stacked bar charts using the *ggplot2* package (v.3.5.1) in R (v.4.1) [58].

### Microbiome alpha and beta diversity analyses

We assessed both within-sample (alpha) and between-sample (beta) microbial diversity to compare microbiome structure across phenotypic groups.

Microbial alpha diversity was assessed using three metrics: observed features, Shannon diversity, and Simpson diversity. These metrics were calculated using the alpha-group-significance command, which implements a Kruskal-Wallis test to identify differences in diversity distributions.

For beta diversity, a pseudocount of 1 was added to the genus- and species-level fecal and saliva feature tables (--p-pseudocount 1). The data were transformed using the Aitchison distance metric (--p-metric aitchison) to account for the compositional characteristics of microbiome data [59–61]. The resulting Aitchison distance matrices were visualized using principal coordinates analysis (PCoA) to assess patterns of microbial community structure. Group-level differences in beta diversity were tested using PERMANOVA (permutational multivariate analysis of variance), as implemented in the qiime diversity beta-group-significance function.

### Differential abundance analysis

Maaslin2 (Multivariable Association with Linear Models 2) in R (v.4.1) was used to identify differentially abundant microbial species. Separate analyses were performed for each species table (Greengenes2, SILVA, MIMt, and GSR). For each analysis, the following parameters were used: input – species abundance table in CSV format, seed set to 1, maximum significance threshold set to 0.05, and the metadata phenotypes were set to “Typical,” “Apraxia of speech,” and “Dyslexia.” Statistical significance was set to corrected p-values (*i.e., q-values)* equal to or less than 0.05.

### Predicted function analysis with PICRUSt2

Predicted microbial function was assessed using the Phylogenetic Investigation of Communities by Reconstruction of Unobserved States 2 (PICRUSt2) pipeline (version 2.4.1) [62]. Functional prediction was performed using the *picrust2_pipeline.py* command with the default parameters, which executes a streamlined workflow including phylogenetic placement, hidden-state prediction, and functional inference. This pipeline integrates several steps: (1) placing ASVs into a reference phylogeny using EPA-NG, (2) inferring gene family copy numbers based on ancestral-state reconstruction, and (3) mapping those predictions to KEGG Ortholog (KO) groups, Enzyme Commission (EC) numbers, and MetaCyc metabolic pathways.

Prior to differential abundance analysis with MaAsLin2, the predicted functional profiles were filtered to remove low-prevalence features, excluding those present in fewer than 10% of samples to minimize noise and reduce the chances of spurious associations. This filtering step was applied to KO, EC, and pathways tables independently. Separate MaAsLin2 models were run for each functional table (KO, EC, and MetaCyc pathways). Statistical significance was determined using FDR-corrected q-values, with a threshold of *q* ≤ 0.05.

## Results

### Distinct fecal microbial communities in individuals with dyslexia compared to CAS and neurotypical participants

#### Alpha diversity.

**Fig 2:**
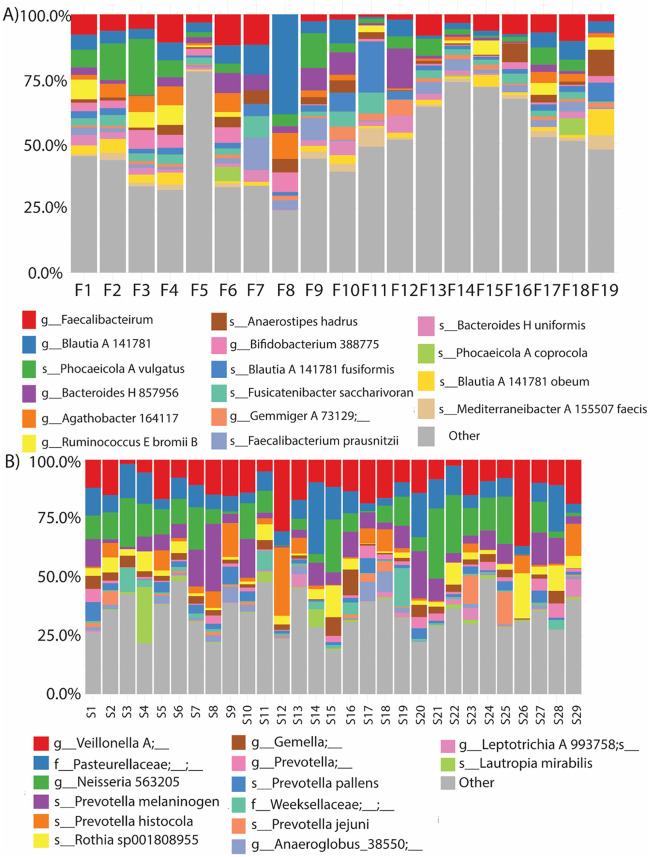
Relative abundance of microbial communities across all participants. (A) Fecal microbiome composition showing ASVs with a minimum relative abundance of 5% and present in at least two samples. (B) Salivary microbiome composition showing ASVs meeting the same inclusion criteria (≥5% relative abundance and present in at least two samples). ASVs not meeting these thresholds are grouped into the remaining ‘Other’ category.

We first assessed the relative abundance of prevalent taxa across fecal samples using taxonomic assignments based on the Greengenes2 database. In the fecal microbiome dataset, the most prevalent taxa included members from the genera *Blautia, Faecalibacterium,* and *Phocaeicola*, each comprising at least 5% relative abundance and detected in at least two samples (Fig 2A). However, there was a long tail of low abundant taxa, with the “Other” category accounting for nearly half of the community across samples (mean relative abundance = 49%).

**Fig 3:**
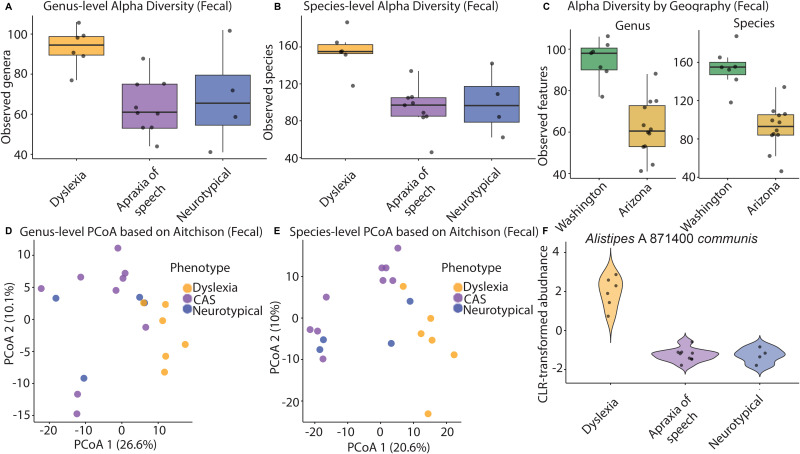
Diversity and compositional differences in fecal microbiomes across phenotypes. (A) Genus-level alpha diversity, colored by phenotype. (B) Species-level alpha diversity by phenotype. (C) Alpha diversity at both genus and species levels stratified by geographic residence. (D) Principal coordinates analysis (PCoA) of genus-level composition based on Aitchison distance. (E) Species-level PCoA based on Aitchison distance, colored by phenotype. (F) Differential abundance results from MaAsLin2 highlighting *Alistipes* A 871400 communis; the y-axis represents centered log-ratio (CLR)–transformed abundances. Collectively, these results suggest that individuals with dyslexia may exhibit distinct gut microbiome signatures relative to other groups.

At the genus level, individuals with dyslexia exhibited greater alpha diversity in their fecal microbiome compared to individuals with CAS history (H = 9.423, *q* = 0.006), though the difference between the dyslexia and neurotypical individuals was not significant (H = 2.227, *q* = 0.203) (Fig 3A; S2 Table in S1 File).

This trend was slightly different at the species level where individuals with dyslexia exhibited higher alpha diversity compared to neurotypical controls (H = 5.534, *q* = 0.028) and the individuals with CAS history (H = 9.406, *q* = 0.006) (Fig 3B; S2 Table in S1 File). This pattern remained consistent when comparing alpha diversity using both Shannon diversity and Simpson diversity (S3 Table in S1 File). Individuals with dyslexia also had a higher alpha diversity than when using the species-level datasets based on the GSR-DB, MIMt, and SILVA databases (S4 Table in S1 File). However, geographic location may represent a potential confounding factor as participants from Washington exhibited significantly higher alpha diversity than those in Arizona at both the genus level (H = 12.028, *q* = 0.0005) and species level (H = 12.028, *q* = 0.0005) (Fig 3C; S2-S3 Tables in S1 File).

#### Beta diversity.

We also explored the beta diversity to assess whether overall microbial community composition differed across diagnostic groups. At the genus level, dyslexic individuals exhibited significantly different microbial communities from neurotypical individuals (pseudo-F = 2.062, q = 0.005), and those with CAS (pseudo-F = 3.494, q = 0.003) (Fig 3D; S2 Table in S1 File). No significant differences were observed between typical individuals and those with CAS (S2 Table in S1 File).

The species-level analysis indicated individuals with dyslexia had a significantly distinct microbial community from that of the neurotypical (pseudo-F = 1.740, *q* = 0.009) and CAS groups (pseudo-F = 2.845, *q* = 0.003) (Fig 3E; S3 Table in S1 File), independently. In contrast, the beta diversity did not differ significantly between CAS and neurotypical groups (pseudo-F = 1.098, *q* = 0.263). This trend was further supported by analyses based on species-level datasets using the GSR-DB, MIMt, and SILVA taxonomic reference databases (S5 Table in S1 File).

Using the Greengenes2 database, geographic location was identified as a significant confounding factor at both the genus (pseudo-F = 3.497, *q* = 0.002) and species levels (pseudo-F = 2.737, *q* = 0.001). However, no significant differences were observed with respect to age.

### Differential abundance analyses

At the species level, MaAsLin2 identified a significantly higher abundance of *Alistipes A 871400 communis* in individuals with dyslexia compared to both neurotypical family members and individuals with CAS (β = 2.575, *q* = 0.004) (Fig 3F; S6 Table in S1 File). However, this result may also be confounded by geography as *Alistipes A 871400 communis* was significantly enriched in the Washington group compared to the Arizona group (β = 2.206, *q* = 0.003; S7 Table in S1 File). These results are unlikely to be confounded by age, as *Alistipes* was not significantly associated with age (S8 Table in supporting information).

### High similarity in salivary microbial community structure across groups

#### Alpha diversity.

Compared to the fecal samples, the saliva microbiome of the participants showed a more even distribution across top taxa, though a substantial portion of the community was represented in the “Other” category (mean relative abundance = 34%) (Fig 2B). The most prevalent taxa across the salivary dataset were *Vellonella A, Pasteurellaceae,* and *Neisseria* 563205.

**Fig 4:**
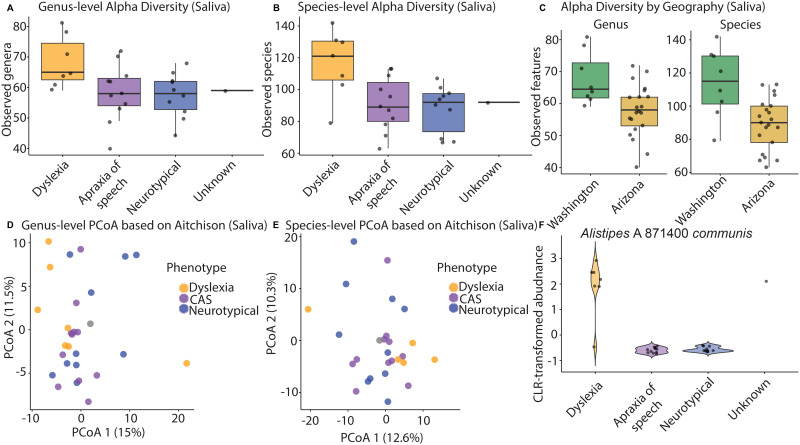
Diversity and compositional patterns in salivary microbiomes across phenotypes. (A) Genus-level alpha diversity by phenotype. (B) Species-level alpha diversity by phenotype. (C) Alpha diversity at both genus and species levels stratified by geographic residence. (D) Principal coordinates analysis (PCoA) of genus-level composition based on Aitchison distance, colored by phenotype. (E) Species-level PCoA based on Aitchison distance, colored by phenotype. (F) Differential abundance results from MaAsLin2; the y-axis represents centered log-ratio (CLR)–transformed abundances. In contrast to fecal microbiome results, these analyses suggest that dyslexia is not associated with detectable differences in the salivary microbiome.

While not statistically significant, individuals with dyslexia tended to exhibit a higher alpha diversity compared to those with CAS and their neurotypical family members at the genus and species levels (Fig 4A-4B; S2-S3 Tables in S1 File). However, both age and location were significant factors when comparing saliva samples at both the species and genus levels (Fig 4C; S2-S3 Table in S1 File).

#### Beta diversity.

At the genus level, individuals with dyslexia did not exhibit a significantly distinct oral microbiome compared to neurotypical individuals (pseudo-F = 1.259, *q* = 0.448) and CAS individuals (pseudo-F = 1.495, *q* = 0.102) (Fig 4D; S2 Table in S1 File). No significant differences in saliva microbiome composition were detected between neurotypical individuals and those with CAS (pseudo-F = 1.037, *q* = 0.315). However, at the species level, individuals with dyslexia exhibit a distinct community composition to individuals with a history of CAS (pseudo-F = 1.751, *q* = 0.048) (Fig 4E). The microbial community composition between individuals with dyslexia and neurotypical individuals was not distinct (pseudo-F = 1.460, *q* = 0.162). However, our analyses also indicated that the microbial communities between individuals residing in Arizona and Washington were significant at both the genus level and species level (Fig 4E; S2-S3 Tables in S1 File).

### Differential abundance analyses

While we did not observe significant differences in alpha or beta diversity in the saliva microbiome dataset, *Treponema C. lecithinolyticum* (β = 1.945, *q* = 6.28x10^-5^) and *Treponema D. amylovorum* (β = 3.467, *q* = 0.048) exhibited significantly higher abundances in individuals with dyslexia compared to both CAS and neurotypical family members (Fig 4F, S9 Table in S1 File). However, *Treponema C. lecithinolyticum* was enriched in the Washington cohort (β = 1.703, q = 9.25x10^-5^) (S10 Table in S1 File). No specific taxa showed significant differences in abundance across age groups (S11 Table in S1 File).

### Differential abundance analysis for potential functional profiles

#### Fecal microbiome dataset.

**Fig 5:**
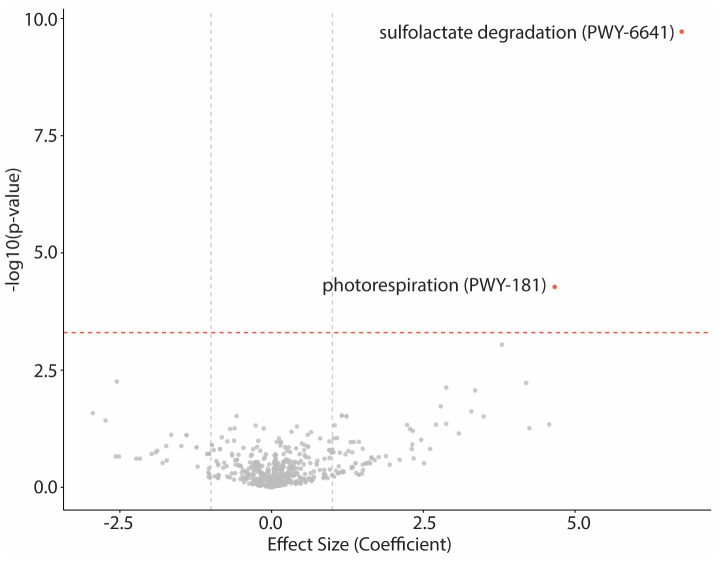
Differentially abundant functional pathways in the fecal microbiome inferred using PICRUSt and tested with MaAsLin2. Volcano plot displays the association between pathway abundance and phenotype, with effect size on the x-axis and statistical significance (−log10 *p*-value) on the y-axis. Pathways passing multiple testing correction are highlighted, including photorespiration (PWY-181) and sulfolactate degradation (PWY-6641), both of which exhibited moderate effect sizes and were enriched in the dyslexia group. These results indicate functional differences in predicted metabolic potential across groups.

We conducted a MaAsLin2 analysis on PICRUSt-based functional potential profiles for both the fecal and saliva microbiome datasets. For the fecal dataset, individuals with dyslexia showed a significantly higher predicted abundance of sulfolactate degradation (MetaCyc pathway PWY-6641, β = 6.76, *q* = 1.37x10^-7^) and photorespiration (MetaCyc pathway PWY-181, β = 4.66, *q* = 0.019) compared to neurotypical participants and individuals with CAS (Fig 5; S12 Table in S1 File). Further analysis showed that the enzyme sulfoacetaldehyde acetyltransferase was enriched in the individuals with dyslexia (EC 2.3.3.15, β = 0.58, *q* = 3.72x10^-6^, S13 Table in S1 File). However, as with the previous analyses, the enrichment of these features may be due to geographic differences. The sulfolactate degradation pathway (PWY-6641, β = 6.41, q = 4.37x10^-6^), photorespiration pathway (PWY-181, β = 6.28, q = 3.12x10^-5^), and sulfoacetaldehyde acetyltransferase (EC 2.3.3.15, β = 7.25, q = 4.51x10^-6^) were enriched in individuals living in Washington (S14-S16 Tables in S1 File).

#### Saliva microbiome dataset.

As for the saliva microbiota dataset, PICRUSt2 did not identify any significant predicted pathways or EC enzyme features after multiple testing correction (S17-S18 Tables in S1 File).

## Discussion

This exploratory study is, to our knowledge, the first to examine both the fecal and saliva microbiota of individuals with dyslexia, those with CAS histories, and their neurotypical, first-degree relatives. Although our sample size is relatively small, and thus limits the statistical power of our analyses, the family-based cohort scheme employed here has been effective in other microbiome studies [63]. While the present findings should be interpreted as hypothesis-generating rather than confirmatory, the patterns observed highlight the potential for microbiome analyses to provide new perspectives on the biological underpinnings linked to NDDs. Integrating neurological assessments with microbiome data offers a promising foundation for developing complementary diagnostic approaches and informing future studies aimed at targeted therapeutic treatments.

### Individuals with dyslexia exhibit distinct fecal and salivary microbial profiles from CAS and neurotypical family members

Dyslexia cannot be diagnosed and treated until the early school years when difficulties with written language begin to emerge. Identifying biological markers that could supplement current diagnostic practices may eventually enable earlier risk stratification and intervention. Microbiome-informed approaches represent one possible avenue, though at present such applications remain highly exploratory.

In this study, we observed preliminary differences in both the fecal and salivary microbiomes of individuals with dyslexia compared to neurotypical family members and those with histories of CAS. In the fecal dataset, individuals with dyslexia exhibited higher alpha diversity and a distinct microbial community structure, with *Alistipes communis* A871400 enriched relative to neurotypical individuals. This finding is intriguing because *Alistipes* taxa have previously been linked to depression, anxiety, and ASD [64,65]. Several studies have reported elevated levels of *Alistipes* in individuals with ASD compared to neurotypical controls [36,66,67]. One proposed mechanism to explain these findings is that certain *Alistipes* species secrete glutamate decarboxylase, an enzyme that catalyzes the transformation of glutamate into γ-aminobutyric acid (GABA), an inhibitory neurotransmitter in the central nervous system [68]. The elevated levels of *Alistipes* in individuals with dyslexia in our study may support this hypothesis. However, whether *Alistipes* is directly involved with dyslexia cannot be inferred because of the cross-sectional nature of the study, coupled with its modest sample size. Further research incorporating larger, independent cohorts and integrated multi-omic approaches, including shotgun metagenomics, metabolomics, and other functional profiling methods, will be needed to understand whether this taxon plays an active role in neurodevelopmental processes, reflects downstream consequences of shared environmental or dietary factors, or represents a correlated but unrelated signal.

In the salivary microbiome, overall alpha and beta diversity did not differ between groups based on phenotype. This pattern may reflect the fact that the oral microbiome is generally more stable and less susceptible to short-term changes compared to the gut [69–71]. While we did not observe community structure differences, we identified two *Treponema* species enriched in the individuals with dyslexia, specifically *Treponema C. lecithinolyticum* and *Treponema D. amylovorum*. *Treponema* species are traditionally associated with periodontal disease [72–74], but have recently also been implicated in neurodegenerative conditions [75–79]. For instance, both *T. lecithinolyticum* and *T. amylovorum* were identified in Alzheimer’s Disease brain tissue [76]. These findings further support the notion that shared pathways between the mouth and gut may jointly modulate host physiology, including cognition.

Predicted functional profiling with PICRUSt2 provided additional exploratory insights. Individuals with dyslexia exhibited a higher abundance of microbial pathways related to sulfur metabolism and photorespiration. Sulfur metabolism has been implicated in ASD, AD, and Parkinson’s disease [80–82]. Additionally, the photorespiration pathway was enriched in the dyslexia group. While typically associated with plant metabolic cycles, photorespiration in microbial systems has been linked to glyoxylate and serine metabolism, both of which are involved in cellular redox balance and amino acid processing [83]. As such, the pathway’s enrichment may reflect shifts in the gut microbial function related to nitrogen cycling, oxidative stress regulation, or carbon overflow metabolism [84]. While these predicted functional differences suggest that individuals with dyslexia may harbor fecal microbiomes with altered sulfur metabolism and amino acid processing capabilities, these predictions are based on 16S data and should be considered hypothesis-generating only.

Together, our results suggest that the microbial community structure of individuals with dyslexia may be unique from CAS and neurotypical family members. The stronger associations in the fecal dataset, relative to the saliva microbiome, may reflect the greater sensitivity of the gut microbiota to neurodevelopmental and behavioral factors—a pattern that has also been reported in ASD studies [85]. For instance, Qiao et al., (2018) reported that salivary samples showed no difference in richness and diversity between ASD and non-ASD children [86]. However, because geographic location was confounded by phenotype, we were unable to disentangle whether the observed microbiome differences were attributable to neurodevelopmental status or geographic variation, which is known to influence oral microbiome composition [87]. Future studies will need to more carefully account for geography, as well as other potential confounders—age, sex, BMI, diet, antibiotic use, and oral health status—all of which are known to influence the gut and oral microbiome [88–93].

### Consistency of fecal and saliva microbiomes findings across 16S rRNA databases

Taxonomic classification remains a major challenge in microbiome bioinformatics, with one persistent issue being the use of different marker gene reference databases [94]. To account for this issue, we classified our 16S rRNA data using four 16S rRNA databases: Greengenes2, GSR-DB, MIMt, SILVA. Overall, our findings were largely consistent across comparisons. For example, the patterns of higher alpha diversity in individuals with dyslexia was consistent across all four datasets. Similarly, we did not detect differences in the oral microbiome datasets across phenotypic comparisons. This finding is consistent with other studies indicating that the gut microbial community structure is more responsive to systemic physiological and behavioral factors than the oral microbiome [70,95–97]. Furthemore, the enrichment of *Treponema* species in the oral cavity of individuals with dyslexia may provide valuable systems-level insights and underscore the importance of considering both oral and gut sites when investigating speech and language phenotypes.

While the community-level results were consistent across datasets using different taxonomic databases, we observed discrepancies in the differential abundance analyses. For example, *Alistipes communis* was identified as significantly enriched in individuals with dyslexia in the Greengenes2 database and the GSR dataset. *Alistipes communis* could not be evaluated for differential abundance in the MIMt- or SILVA-based analyses because this species was not included in those reference databases. This highlights how differences in database references can affect downstream results and highlights the importance of careful database selection.

### Limitations

The small sample size in this study does not fully represent the broader population. The study cohort, for example, was predominantly White. Moreover, the limited sample size in the present cohort precluded formal assessment of inter-sex variability, which has also been reported as a significant factor [88,98]. The inclusion of both sibling and parental controls, where age-related differences could contribute to the inter-individual variability, is another limitation. While we conducted age-based comparisons, the limited number of samples precluded formal statistical adjustment for age in our models. Moreover, we recognize that person-to-person transmission plays a significant role in shaping both the gut and oral microbiome [89], which may further confound interpretations in family-based cohort studies. In future studies, the use of age-matched sibling controls and designs that account for household-level microbial sharing may help disentangle host-genetic effects from environmentally acquired microbiota. In turn, understanding the relationship between host genetics and the microbiome may offer additional insights into the biological mechanisms underlying dyslexia. Such efforts will require substantially larger sample sizes to achieve sufficient statistical power, as demonstrated in large-scale studies investigating host genetic-microbiome associations [99–103]. In short, larger cross-sectional and longitudinal studies are needed to validate these associations, explore potential causal mechanisms, and evaluate the diagnostic and therapeutic potential of the oral-gut axis in treating dyslexia.

Lastly, this study did not include extraction blanks or PCR-negative controls, which limits the ability to formally identify and remove potential reagent- or laboratory-derived contaminants. The absence of negative controls necessitates cautious interpretation of low-abundance taxa. Future studies should incorporate negative controls at multiple stages of the wet-lab pipeline and apply contamination-sensitive filtering approaches to more robustly distinguish biological signal from technical noise [104,105].

## Conclusion

Using a family-based cohort design, this study combined high-throughput 16S rRNA gene sequencing with phenotype data to examine associations between gut and oral microbiomes and dyslexia and CAS. We observed differences in gut microbial diversity, taxonomic composition, and predicted functional potential between individuals with and without dyslexia, as well as enrichment of specific *Treponema* species in the salivary microbiomes of individuals with dyslexia. These findings represent associative patterns and should be interpreted in the context of the study’s exploratory design and the potential influence of familial clustering and unmeasured confounding factors.

This study broadens the scope of microbiome research to include underexplored language-based neurodevelopmental conditions and highlights the value of interdisciplinary, systems-level approaches to understanding the biological correlates of language and learning. By integrating microbiome data with phenotype information in a family-based framework, our findings contribute to a growing body of evidence recognizing the oral-gut-brain axis as a relevant biological system for studying neurodevelopment and neurocognition. As such, these results provide a foundation for future research aimed at clarifying the role of microbial communities in dyslexia and related communication disorders.

## Supporting information

S1 Appendix Supporing informationabout diagnostic criteria, quality assessment of the sequencing data, database reference results, and pedigree information. (PDF)

S1 File Supporting information files including S1-S18 Tables. (XLSX)
