# Supplementary material for: Distinct gut and oral microbiome patterns associated with dyslexia in a family-based cohort: A preliminary exploratory study
Source: PLoS One. 2026 Jul 21;21(7):e0353463. doi: 10.1371/journal.pone.0353463 (PMC13387549; doi:10.1371/journal.pone.0353463)
Supplement: S1 Appendix — (PDF) [file pone.0353463.s001.pdf]

Appendix

Contextual information for diagnostic criteria

This section provides additional context on the diagnostic criteria and procedures used to determine dyslexia and childhood apraxia of speech (CAS) status in our study participants.

All assessments were conducted in a research lab setting at the University of Washington in Seattle and administered by trained research staff. Scoring was done offline, using audio- and video-recorded session data. Scores were checked by a second lab member to ensure reliability across participants.

Early diagnosis of CAS and dyslexia is complicated by overlapping symptoms with other neurodevelopmental conditions, the wide variability in typical language acquisition, and the limitations of standardized assessments [1]. What appears to be a delay in speech or reading may fall within the bounds of typical development, making it difficult to distinguish children who require clinical intervention from those who do not [2–4]. Cultural and linguistic differences can further obscure accurate identification, particularly when assessment tools are not adapted for diverse backgrounds [5]. Collectively, these factors contribute to delayed or missed diagnoses, which in turn can postpone access to essential therapeutic services.

It has been hypothesized that CAS is influenced by cerebellar dysfunctions, likely due to genetic influences [6–12]. Adults with a history of CAS continue to exhibit difficulties with motor coordination in their speech and tasks that require sequential processing of information consistent, with a suspected cerebellar locus of impairment [7,9,12].

Many individuals diagnosed with dyslexia struggle with word reading, decoding, spelling, and reading fluency, and difficulty with parsing speech sounds in words is common. These speech and language challenges have prompted increased interest in the neural underpinnings of dyslexia, particularly the role of the cerebellum in language processing [13–15].

The observation that CAS and dyslexia often co-occur in the same children has been interpreted as stemming from shared deficits in phonological awareness and motor coordination [1,16] and shared cerebellar dysfunctions [9]. From a clinical perspective, both CAS and dyslexia are difficult to diagnose early, as symptoms typically do not emerge, or are not identifiable, until children reach key developmental milestones. For CAS, diagnosis typically occurs when a child begins to produce speech between the ages of 2 to 4 years, while dyslexia often goes unrecognized until the elementary school years, when children fail to make expected progress with formal reading instruction. Even after diagnosis, treatment can be lengthy and costly for CAS [17] and dyslexia [18].

In our study, participants were identified as having dyslexia if they had a professional diagnosis and scored at least 1 standard deviation (SD) below the population mean on at least one of five standardized assessments of written language, described below. All five measures are widely used in research and clinical settings. They were selected to evaluate key areas of written language skills, namely accuracy and speed of sight word reading and phonemic decoding, and additionally, spelling.

To assess accuracy of sight word recognition and nonword decoding, we administered the Word Identification (WID) and Word Attack (WATT) subtests from the Woodcock Reading Mastery Test–Third Edition (WRMT-III) (Woodcock, 2011). These untimed subtests evaluate single-word reading

of real and pseudowords, respectively. For WID, participants are shown a list of sight words with increasing complexity and asked to read each aloud; for WATT, they are presented with pseudowords (i.e., pronounceable letter strings that follow orthographic rules but have no meaning) to assess phonological decoding in the absence of lexical content. Both subtests have basal and ceiling rules and are normed for ages 4 to 9 years, with start items given for each participant age bracket.

To complement these accuracy-based measures, we assessed word-level reading fluency using the Sight Word Reading Efficiency (SWE) and Phonemic Decoding Efficiency (PDE) subtests from the Test of Word Reading Efficiency–Second Edition (TOWRE-2) (Torgesen et al., 2012). These timed subtests require participants to read as many items as possible within 45 seconds. SWE presents a list of real words, while PDE consists of pronounceable pseudowords, both with increasing complexity. Participants are instructed to read each list aloud as quickly and accurately as possible, starting with the first item regardless of age. Each subtest includes 104 items. The number of correctly produced items is the raw score, which is age-normed for individuals ages 6 to 24 years, though they are sometimes used informally with older populations. Including both real-word and pseudoword tasks under time constraints enables researchers to assess automaticity of sight word retrieval and speeded phonemic decoding. By incorporating both timed and untimed measures, we aimed to distinguish between deficits in reading accuracy (i.e., the ability to recognize or decode word correctly) and fluency (i.e., the speed and automaticity of word and nonword reading).

Lastly, conventional spelling skills were assessed using the Spelling subtest of the Wechsler Individual Achievement Test–Third Edition (WIAT-III) (Wechsler, 2009). This untimed subtest requires participants to write orally dictated words of increasing difficulty, assessing their orthographical knowledge. It includes 45 items, with administration continuing until a ceiling is reached based on the number of errors. The WIAT-III is normed for ages 4 to 55 years. Including spelling in a dyslexia assessment is particularly relevant, as spelling deficits often persist even in individuals who have made gains in reading accuracy (Rubenstein et al., 2011). Including this measure provided additional information on participants' phonological processing and orthographic integration, which is also a common deficit observed in dyslexia.

For each of the standardized assessments, raw scores were recorded based on the total number of correct responses and then converted to age-normed standard scores using scoring manuals. These standard scores allowed for comparison across individuals with different age and were used to determine whether a participants' performance fell below -1 SD from the normative mean, which served as the threshold for classification for dyslexia group. The focus of this study was on core word-level skills, which are decoding, fluency, and spelling, hence we did not evaluate other components, however, comprehensive dyslexia evaluation can include additional areas of reading, including reading comprehension, oral reading fluency, or connected reading at the passage level. For additional details on our dyslexia phenotyping, see previous publications (Peter et al., 2020; Peter, Andria, et al., 2021; Peter et al., 2018; Peter et al., 2019).

To assess speech motor planning and execution associated with childhood apraxia of speech (CAS), we administered a comprehensive battery of motor speech and articulation assessments. These included the Goldman-Fristoe Test of Articulation–Third Edition (GFTA-3; Goldman, 2015), which evaluates speech sound production, the Syllable Repetition Task (SRT; Shriberg & Lohmeier, 2008), which targets phonological memory and motor planning through syllable combination, and finger tapping tasks, which assess fine motor timing. Participants also completed oral diadochokinetic

(DDK) task which evaluate the speed and coordination of articulatory movements independent of lexical or syntactic demands (Fletcher, 1972).

Unlike dyslexia, CAS does not have a universally accepted, operationalized diagnostic protocol with high sensitivity of specificity that distinguish CAS from other speech sound disorders (Shriberg et al., 2012). As a result, diagnosis often relies on a combination of expert judgement and objective motor speech assessments. Among these, DDK is frequently assessed as part of an oro-motor assessment (Murrell, 2023). Individuals with CAS often demonstrate slowed DDK rates and disrupted sequencing during DDK tasks, reflecting impaired speech motor planning and execution (Murray et al., 2015; Peter, Bruce, et al., 2021). Hence, although the tasks we administered collectively offer a rich dataset, we focused on our CAS classification on oral DDK performance, as it provides a well-established index of articulatory timing and coordination.

Participants were asked to perform rapid repetition of monosyllabic (e.g., “pa”), bisyllabic (e.g., “pata”), and/or trisyllabic (e.g., “pataka”) sequences. These tasks provide quantifiable data on articulatory timing and sequencing, typically expressed in syllables per second. Raw DDK rates were recorded for each participant, including the total number of syllables produced, the total duration of the sequence, and the percent of syllables produced correctly. From these values, the average syllable duration was calculated by dividing total duration by the number of syllables produced. To enable standardized interpretation across individuals, z-scores were calculated to reflect speech timing.

While DDK tasks alone are not sufficient for a diagnosis of CAS, they provide a structured and objective methods for evaluating one of its most distinct features: impaired speech motor coordination. In clinical practice, additional tools such as dynamic motor speech assessments and prosody evaluations are often used to supplement DDK performance. However, in this study, DDK tasks and derived z-scores were used to identify speech motor deficits and classify participants into the CAS group in cases where a formal professional diagnosis was not available. Additional details on our CAS phenotyping procedures can be found in previous publications (Button et al., 2013; Peter, 2018; Peter et al., 2018; Peter et al., 2013; Peter & Raskind, 2011; Peter et al., 2016).

Quality assessment of the sequencing data

To evaluate the quality of sequencing data prior to downstream analysis, we assessed three key metrics for each sample: total sequence count, median fragment, and GC content (%). These quality metrics were chosen because they can provide insights into the accuracy of the downstream analyses.

These metrics were summarized across samples by sample type (Figure S1). By evaluating these indicators independently for fecal and saliva samples, we ensured that quality thresholds were appropriate for the biological context of each sample type.

There was a considerable amount of variability for the number of sequences across the fecal and saliva samples (Figure S1A). The mean number of sequences for the fecal samples was 66,529 (SD=31,918), while the mean number of sequences for the saliva samples was 98,714 (SD=23,372). As expected for 16S rRNA amplicon data, the mean for the median fragment lengths for both fecal (mean=243, SD =0) and saliva samples (mean=253, SD=0.258) was consistent (Figure S1B). Lastly, it did not appear that there was a strong bias in GC content (%) for either fecal (mean=52.2, SD=0.654) or saliva sequences (mean=51.9, SD=0.424) (Figure S1C). Together, these results indicate that there was little technical variability within the fecal and saliva datasets.

Rationale for utilizing four taxonomic databases

For many years, the SILVA database has served as a foundational resource for 16S rRNA gene-based microbiome studies. Its widespread use was largely due to its status as one of the largest and most comprehensive 16S databases available, providing extensive coverage of aligned, quality-checked small subunit (SSU) rRNA gene sequences [19,20]. However, the landscape of 16S reference databases has expanded significantly in recent years. The introduction of updated resources such as Greengenes2 [21], along with the emergence of newer databases like GSR-DB [22] and MIMt [23], highlights the value of incorporating and validating multiple reference frameworks. These newer databases offer alternative taxonomic curation strategies and provide opportunities to assess the consistency of taxonomic assignments across varying database structures. For instance, these databases differ in content (some prioritize specific regions (V3 or V4), while others rely on the full-length of the 16S rRNA gene), number of reference sequences, curation methods, and how often they are updated [24].

To explore the potential for reference database bias, we aligned our V4 region sequences against multiple databases: Greengenes2 (v.2024.09) [21], SILVA (version 138, 99%) [25], MIMt2.0 [23], and GSR-DB (full-length 16S database) [22]. This approach allowed us to identify discrepancies in taxonomic classification across databases and ensure that observed trends were not dependent on a single reference framework.

Moreover, while we sequenced only the V4 region of the 16S gene, recent studies have demonstrated that taxonomic assignment discrepancies between partial (e.g., V3-V4) and full-length 16S rRNA gene sequences are often minimal at higher taxonomic levels, and can remain reliable down to the species level when aligned to well-curated, full-length reference databases [19,26]. These findings support the methodological validity of assigning species-level taxonomy from V4 reads. However, certain taxa, particularly members of the Enterobacteriaceae family and Clostridiales order, remain poorly resolved using the V4 region alone [27]. This limitation in mitigated in part by databases like Greengenes2, which are optimized for full-length sequences while remaining backward-compatible with short-read amplicons.

For the primary analyses presented in the main text, we prioritized results derived from the Greengenes2 database. Benchmarking studies have demonstrated that Greengenes2 offers highly accurate and consistent classifications, with notably low false positive and false negative rates in both human fecal and oral microbiome datasets [21,28,29]. For example, Nagai et al. (2024) demonstrated that the Greengenes2 database had the strongest minimized taxonomic assignment bias in oral microbiota analysis [29].

With these considerations in mind, we assessed the robustness and reproducibility of our findings by conducting parallel analyses using three additional reference frameworks: GSR-DB, MIMt, and SILVA. These complementary analyses yielded comparable results, including consistent trends of increased alpha diversity in the fecal of individuals with dyslexia, similar trends in beta diversity comparisons for both the fecal and saliva datasets, and overlapping differentially abundant taxa when using the same statistical parameters. This cross-database consistency supports confidence in the stability of our findings and highlights the importance of validating results across multiple reference taxonomies in microbiome research.

Alpha diversity results for GSR-DB, MIMt, and SILVA

Alpha diversity is a critical metric in microbiome research used to assess within-sample microbial richness and evenness. However, diversity estimates can be influenced by database-specific biases in taxonomic classification, which may impact downstream analyses and biological interpretations [30–32]. To account for these potential biases, we classified our sequences using four reference databases: Greengenes2 (v.2024.09) [21], SILVA (version 138, 99%) [25], MIMt2.0 [23], and GSR-DB (full-length 16S database) [22].

Using the observed ASVs metric with the GSR dataset, the fecal microbiome from individuals with dyslexia exhibited a significantly higher alpha diversity compared to the neurotypical individuals (H=5.500, q= 0.029) and individuals with apraxia of speech (H= 8.759, q= 0.009) (Figure S2; Table S3). Alpha diversity utilizing the Shannon diversity index stayed consistent with this trend when comparing individuals with dyslexia to neurotypical controls (H=4.545, q= 0.050) and individuals with apraxia of speech (H=6.125, q= 0.040). The alpha diversity of individuals with apraxia of speech did not exhibit significant differences compared to neurotypical individuals using observed ASVs (Table S3).

Based on observed ASVs for the fecal microbiome sequences aligned to the MIMt database, there was no significant difference in the alpha diversity of fecal samples from individuals with dyslexia compared to the neurotypical controls (H=2.227, q=0.203). However, there was an increase in alpha diversity when compared to individuals with apraxia of speech (H=8.367, q=0.011) (Figure S3; Table S3). Alpha diversity using Shannon diversity index followed this trend between the individuals with dyslexia and neurotypical controls (H=1.636, q= 0.301), but individuals with dyslexia did exhibit a higher alpha diversity than individuals with apraxia of speech (H=8.000, q= 0.014). Alpha diversity using the Simpson metric provided no statistically significant associations when comparing individuals with dyslexia against the neurotypical controls (H=0.727, q=0.440) and individuals with apraxia of speech (H=0.889, q=0.440). Alpha diversity between individuals with apraxia of speech and neurotypical controls was not significantly different.

Lastly, taxonomic profiles using the SILVA database indicated that the observed ASVs for individuals with dyslexia did not exhibit a significantly higher alpha diversity compared to neurotypical controls (H=3.682, q=0.083) but did to individuals with apraxia of speech (H= 9.406, q= 0.006) (Figure S4; Table S3). When comparing the alpha diversity with the Shannon diversity index, individuals with dyslexia demonstrated higher diversity than both the neurotypical controls (H= 5.500, q= 0.029) and individuals with apraxia of speech (H= 8.00, q= 0.014). The alpha diversity using Simpson diversity continued to support higher alpha diversity in individuals with dyslexia when compared to neurotypical controls (H= 6.545, q= 0.016) and individuals with apraxia of speech (H= 7.347, q= 0.016). Consistent with the previous databases, the alpha diversity of individuals with apraxia of speech exhibited no significant differences compared to neurotypical individuals, irrespective of diversity metric.

Regardless of the reference database and diversity metric used, we observed that alpha diversity in saliva did not differ by speech phenotype (Table S3; Figures S5-S8).

In summary, our findings suggest that individuals with dyslexia exhibit higher alpha diversity compared to those with apraxia of speech and their neurotypical family members. Although not all group comparisons reached statistical significance, the boxplots indicate potential trends that may be obscured by the limited sample size. A larger dataset with a similar study design could enhance the robustness and statistical power of these observations.

Beta diversity results for GSR-DB, MIMt, and SILVA

To evaluate between-sample differences in microbial community composition, we performed beta diversity analysis using compositional data methods. A pseudocount of 1 was added to the species-level ASV fecal and saliva feature tables (--p-pseudocount 1) and the dataset was transformed using the Aitchison distance metric (--p-metric aitchison) to account for the compositional characteristics of microbiome data [33–35]. The resulting Aitchison distance matrices were visualized using principal coordinates analysis (PCoA) to assess patterns of microbial community structure. Group-level differences in beta diversity were tested using PERMANOVA (permutational multivariate analysis of variance), as implemented in the qiime diversity beta-group-significance function. which is specifically designed to handle the sparse and compositional nature of microbiome data (Gloor et al. 2017; Gloor et al. 2016; Martino et al. 2019).

To visualize microbial community structure, we generated ordination plots using Emperor in QIIME2 [36], which allowed us to assess clustering patterns by sample type or metadata categories. For statistical evaluation of group-level differences, we applied PERMANOVA (permutational multivariate analysis of variance) with 999 permutations [37,38].

Distinct compositional differences based on phenotype were consistently observed across the GSR, MIMt, and SILVA reference databases (Figures S9–S11; Table S4). For example, analysis using the GSR-DB dataset indicated that individuals with dyslexia exhibited significantly different fecal microbiome compositions compared to neurotypical individuals (pseudo-F = 1.837, q = 0.029) and individuals with apraxia of speech (pseudo-F = 2.521, q = 0.003) (Figure S12 A-B, Table S4). This trend was similarly reflected in the MIMt results, which showed distinct microbial profiles for individuals with dyslexia relative to neurotypical individuals (pseudo-F = 1.674, q = 0.015) and those with apraxia of speech (pseudo-F = 2.799, q = 0.006) (Figure S13 A=B, Table S4). The SILVA database yielded comparable findings, with individuals with dyslexia differing from neurotypical individuals (pseudo-F = 1.738, q = 0.036) and those with apraxia of speech (pseudo-F = 2.836, q = 0.003) (Figure S14, Table S4).

In contrast, no significant differences in fecal microbiome composition were observed between individuals with apraxia of speech and neurotypical individuals in any of the three databases: GSR (pseudo-F = 1.006, q = 0.375), MIMt (pseudo-F = 1.059, q = 0.325), or SILVA (pseudo-F = 0.987, q = 0.419). These results across all databases further support the Greengenes2 findings, highlighting that individuals with dyslexia possess a distinct microbial signature when compared to both neurotypical family members and those with apraxia of speech.

The PERMANOVA results for the saliva microbiome comparisons showed a different trend. Of the GSR, MIMt, and SILVA databases, only the SILVA database returned significant results (Table S4). While individuals with dyslexia exhibited distinct saliva microbiomes compared to individuals with apraxia of speech (pseudo-F= 1.727, q= 0.018), the saliva microbiome of individuals with dyslexia and neurotypical individuals were not statistically distinct (pseudo-F= 1.322, q= 0.267). Similar to the fecal microbiome results, no significant differences in beta diversity were observed between neurotypical individuals and individuals with apraxia of speech, irrespective of taxonomic database (Table S4).

MaAsLin2 results for GSR-DB, MIMt, and SILVA

MaAsLin2 was used to identify differentially abundant microbial species. Separate analyses were performed for each species table from the four databases (Greengenes2, SILVA, MIMt, and GSR). Only individuals with a known phenotype were included in the analysis (*i.e.,* individual with "Unknown" phenotype was excluded from the analysis). For the analysis, the seed was set to 1, maximum significance threshold set to 0.05, and "Phenotype" was set as a fixed effect. Statistical significance was set to corrected p-values (*i.e., q-values)* equal to or less than 0.05.

Interestingly, in the taxonomic dataset based on the GSR database, we found that *Alistipes communis* was enriched in the individuals with dyslexia compared to the CAS-affected and neurotypical family members (β=2.567, q=0.002; Figure S13; Table S13). This finding corroborates our primary results from the Greengenes2-based analysis, where *Alistipes communis* was also enriched in the individuals with dyslexia, supporting the strength of this association across reference databases. *Desulfovibrio* (β=5.240, q=0.001, Figure S16A), and *Noviherbaspirillum* (β=1.453, q=0.048, Figure S16B), were statistically significant in the MIMt dataset (Table S14). Similarly, in the SILVA dataset, we identified enrichment of *Alistipes obesi* (β=2.593, q = 0.001, Figure S17A), *Desulfovibrio* (β=5.268, q = 3.75×10^{-5}, Figure S17B), and *Oscillospiraceae NK4A214* (β=3.108, q = 0.002, Figure S17C) in individuals with dyslexia relative to their CAS-affected and neurotypical family members (Table S15).

In the saliva samples using the GSR database, both the *Treponema lecithinolyticum* (β=1.267×10^{-7}, q=4.509×10^{-7} , Figure S18A) and *Treponema amylovorum* (β=3.484, q=0.031, Figure S18B) were significantly enriched in individuals with dyslexia compared to CAS-affected and neurotypical family members (Table S16). In contrast, no differentially abundant taxa reached statistical significance in the MIMt-based saliva dataset (Table S17). However, *Treponema lecithinolyticum* was also found to be significantly enriched in the dyslexia group in the SILVA-based analysis (β=1.946, q=4.685×10^{-5} , Figure S19, Table S18). These findings suggest that *Treponema lecithinolyticum* may be a consistent microbial marker associated with dyslexia in the oral microbiome. The lack of significance in the MIMt dataset likely reflects how *Treponema* was not identified in the dataset when using this database.

Age as a potential confounding factor

To assess whether age may have contributed to the observed microbiome differences among individuals with dyslexia, CAS, and neurotypical controls, we conducted exploratory analyses incorporating age as a covariate.

We tested associations between age and key microbiome features, including alpha diversity metrics (Observed ASVs, Shannon, and Simpson indices) beta diversity for the fecal (Figures S10 A-D) and saliva (Figures S11 A-D) datasets. While exploratory, these results suggest that age is unlikely to be a major confounding factor in the reported findings and support the robustness of our conclusions regarding phenotype-specific microbiome differences.

Pedigree information

Pedigrees illustrating the familial relationships between affected and unaffected participants are provided in Figures S20-S26. These diagrams detail the distribution of dyslexia, CAS, and neurotypical phenotypes within each family and were used to support phenotype classification.

Supplementary Figures

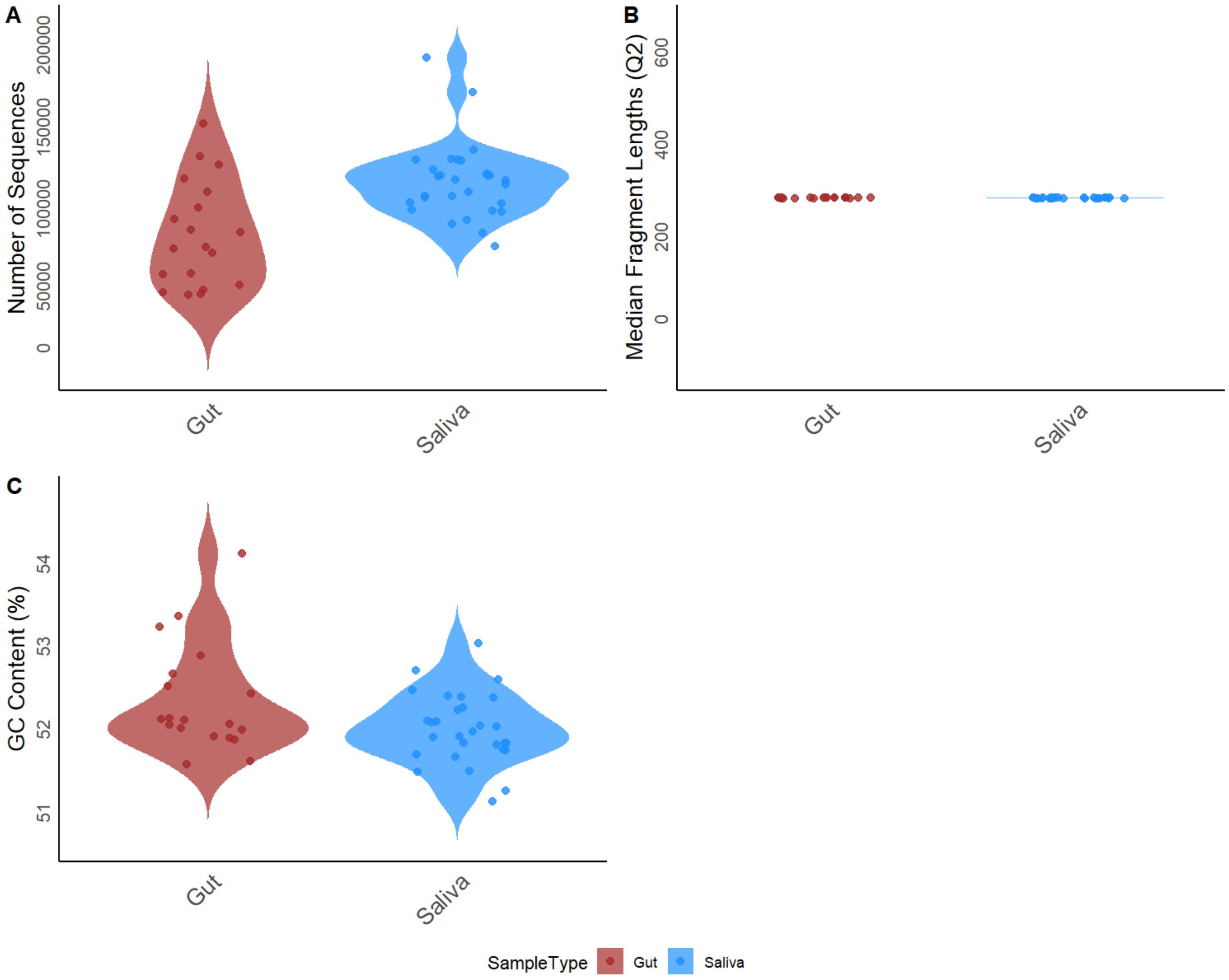

Figure S1. Summary of sequencing quality metrics across samples.

A) Number of sequences per sample, B) Median fragment lengths, and C) GC content per sample. These metrics were used to assess overall sequencing quality and consistency across the dataset.

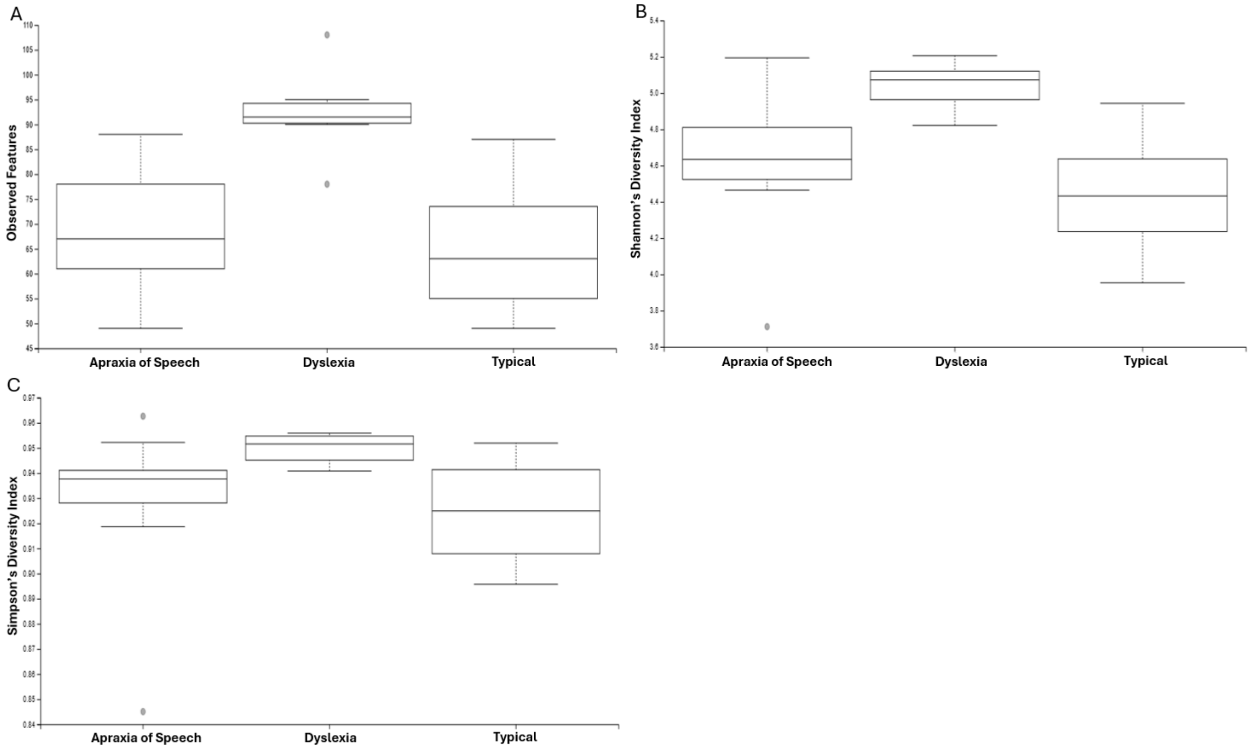

Figure S2. Alpha diversity boxplots of fecal samples aligned against the GSR database.

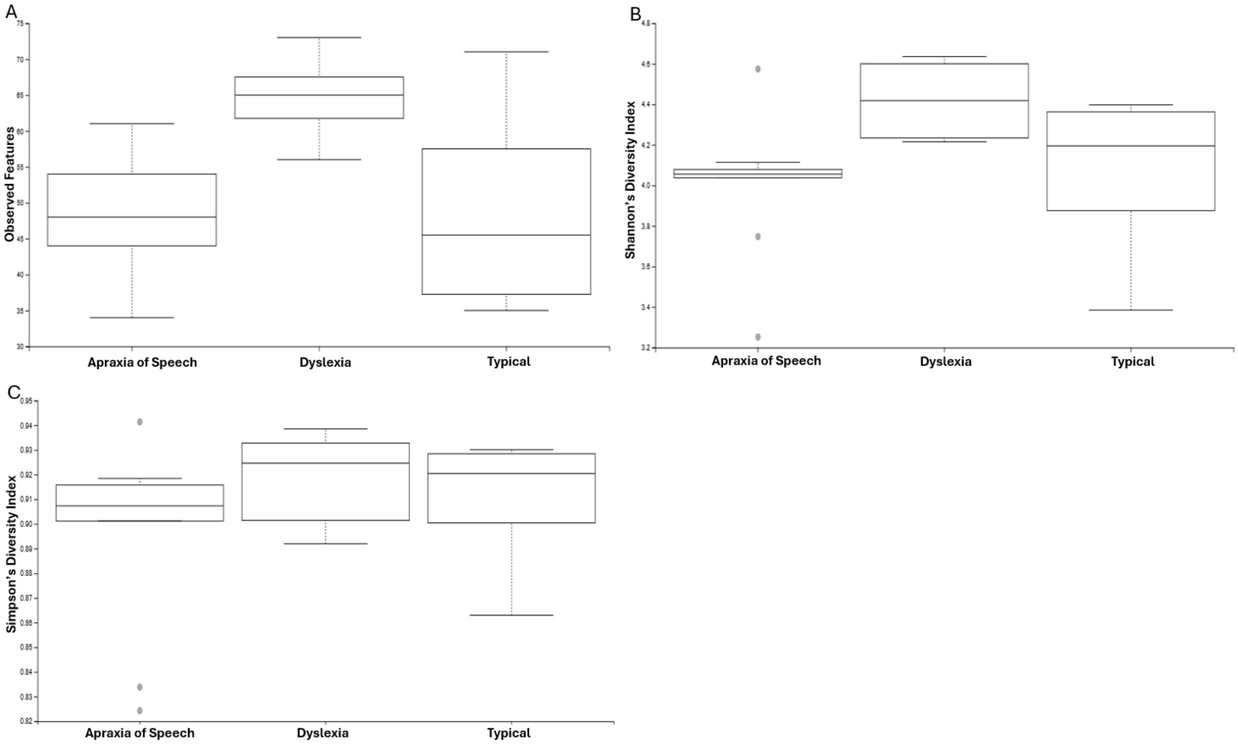

Figure S3. Alpha diversity boxplots of fecal samples aligned against the MIMt database.

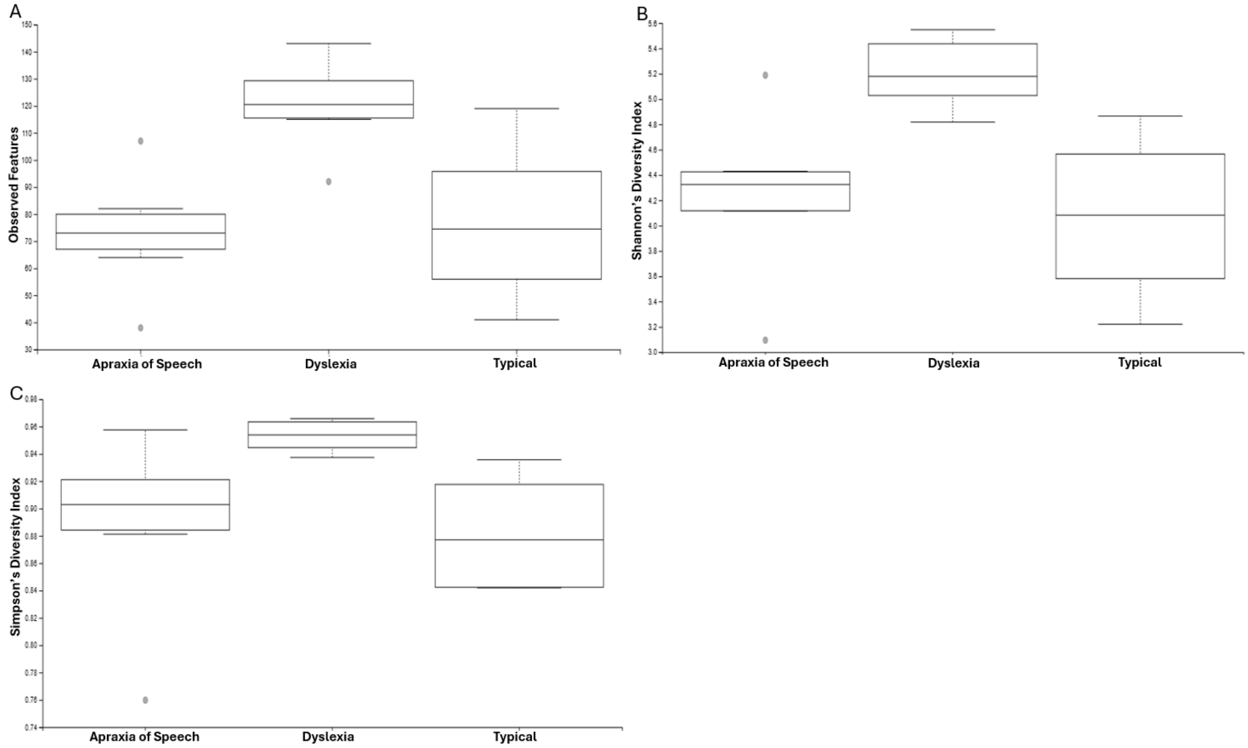

Figure S4. Alpha diversity boxplots of fecal samples aligned against the SILVA-138 database.

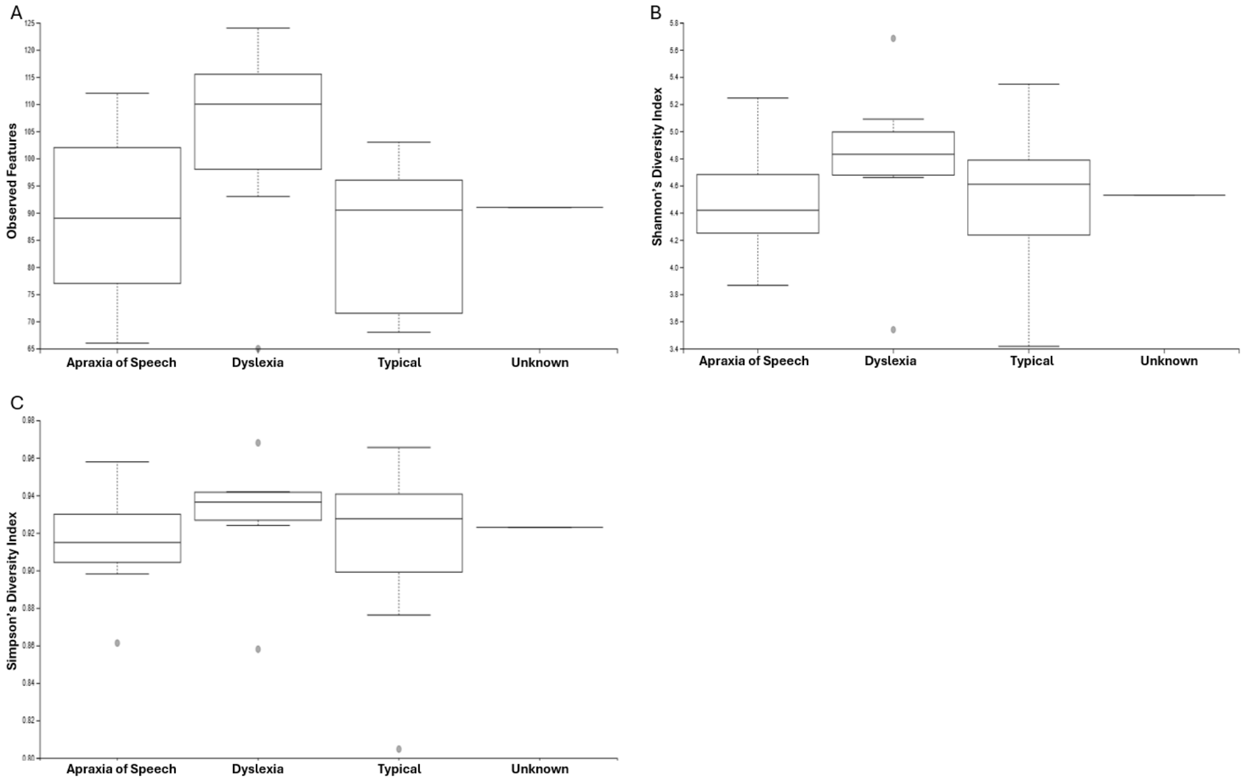

Figure S5. Alpha diversity boxplots of saliva samples aligned against the GSR database.

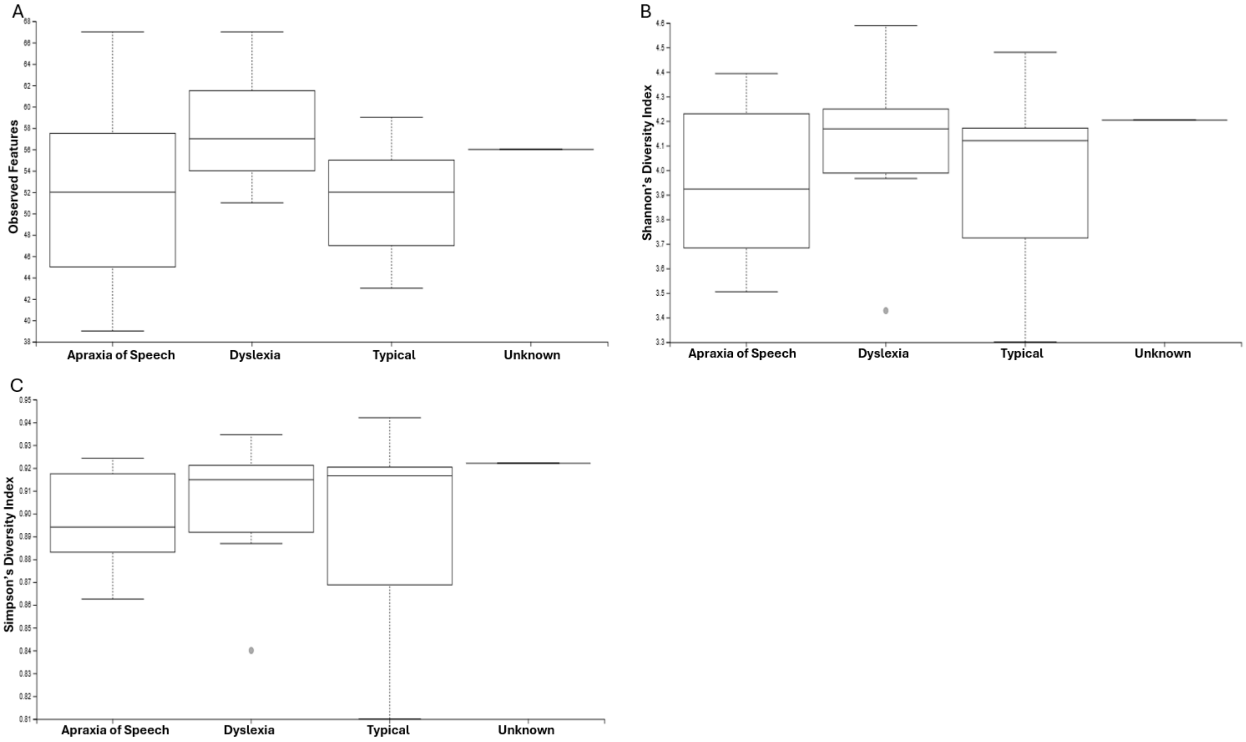

Figure S6. Alpha diversity boxplots of saliva samples aligned against the MIMt database.

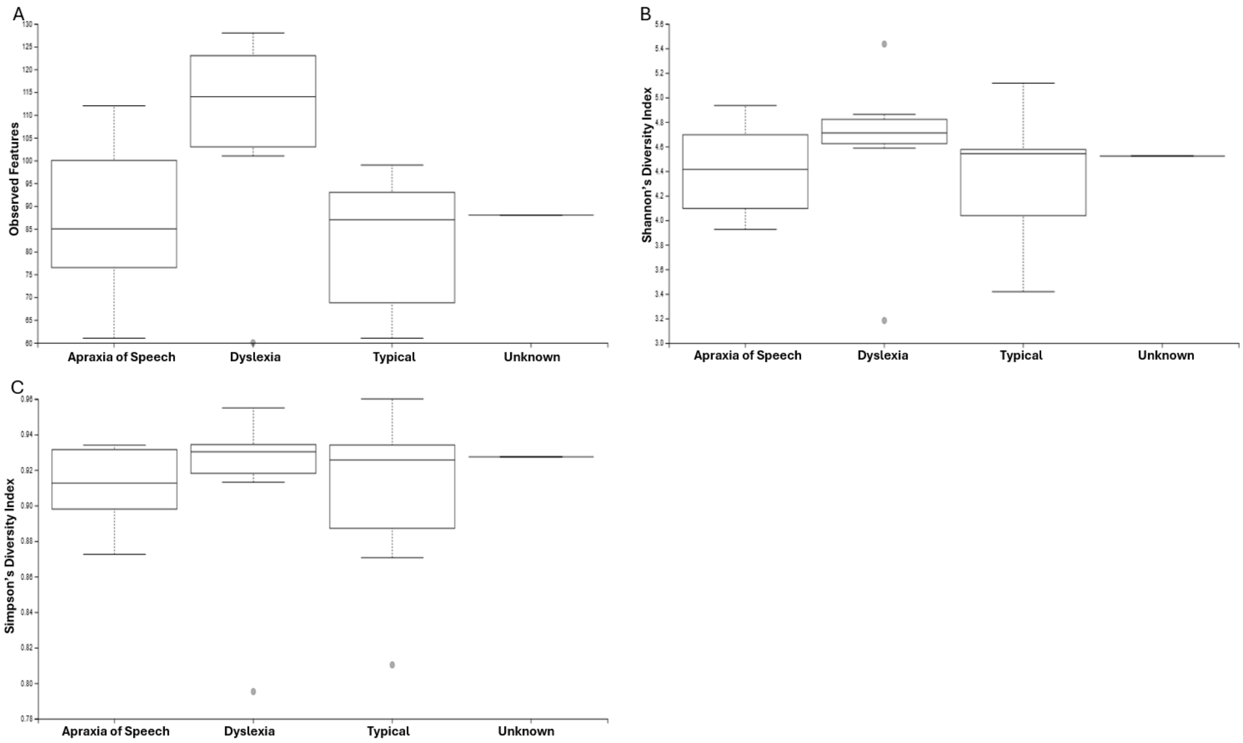

Figure S7. Alpha diversity boxplots of saliva samples aligned against the SILVA-138 database.

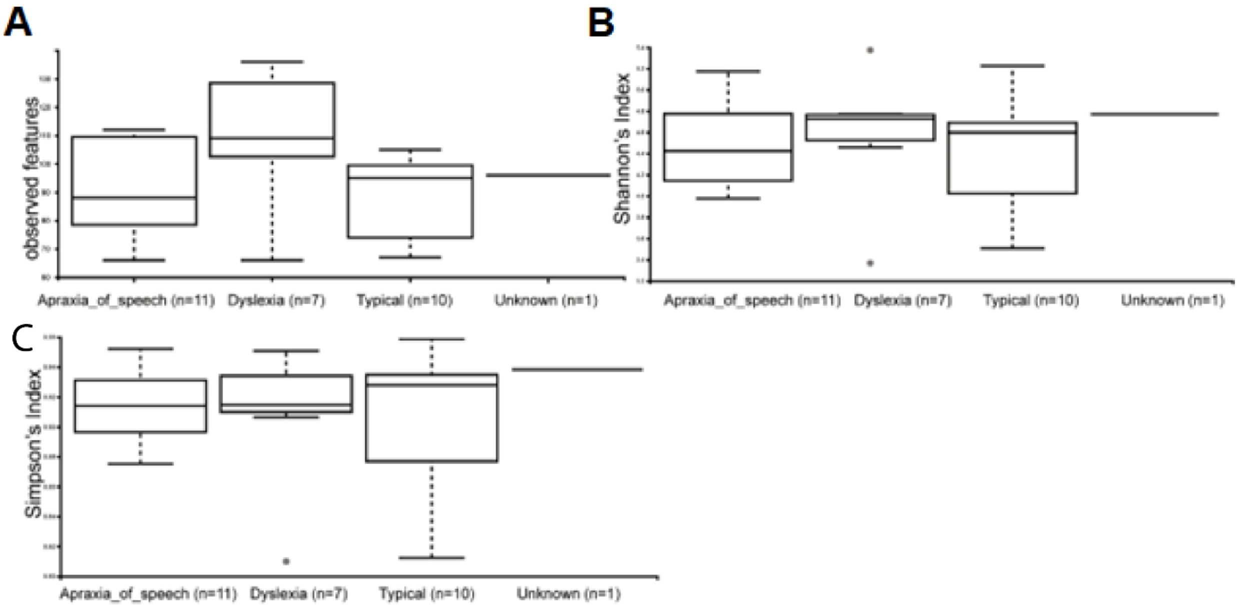

Figure S8. Alpha diversity boxplots of saliva samples aligned against the Greengenes2 database.

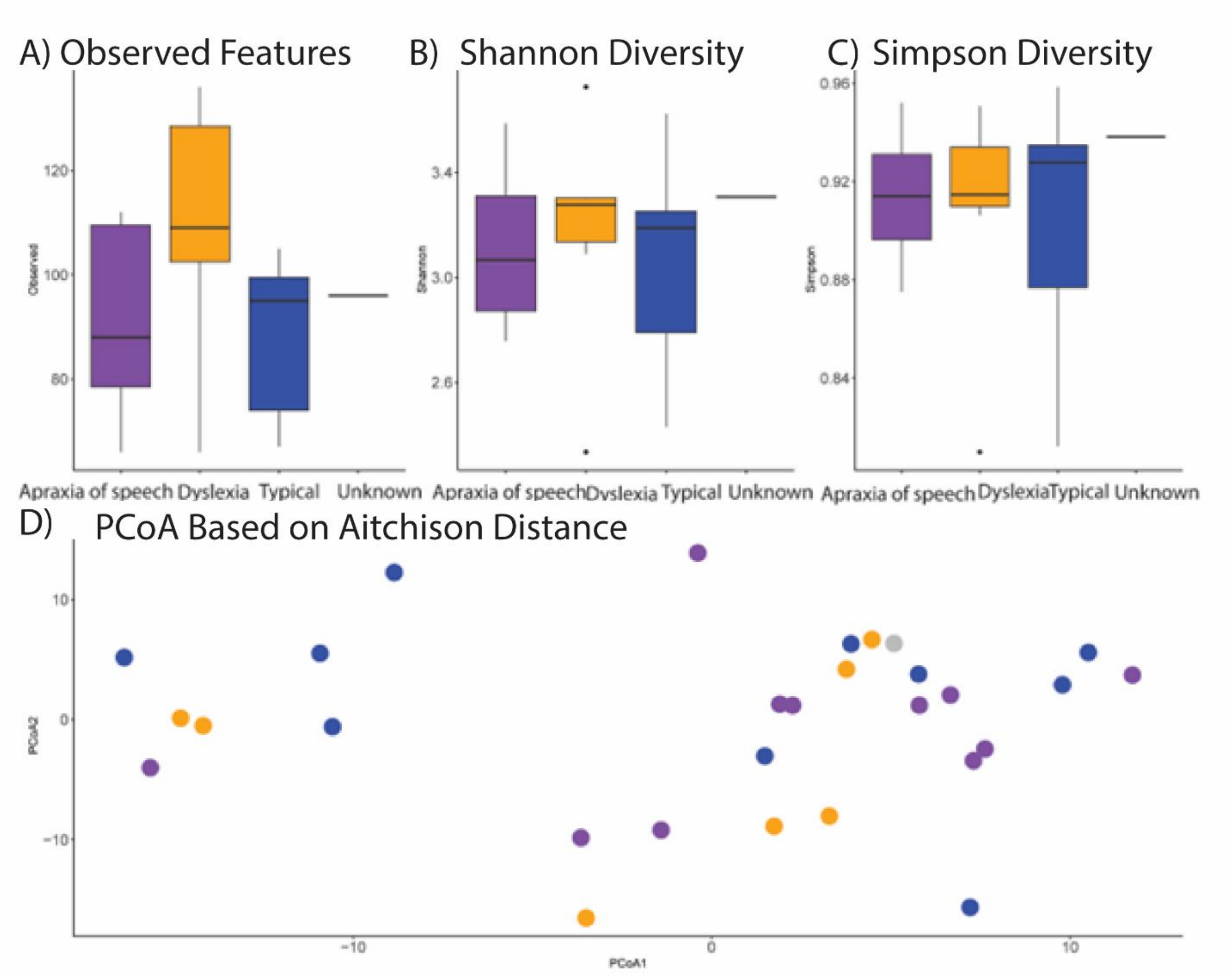

Figure S9. A) Observed features (ASVs) diversity , B) Shannon diversity, C) Simpson diversity and D) PCoA for saliva samples at the species level based on the Greengenes2 database.

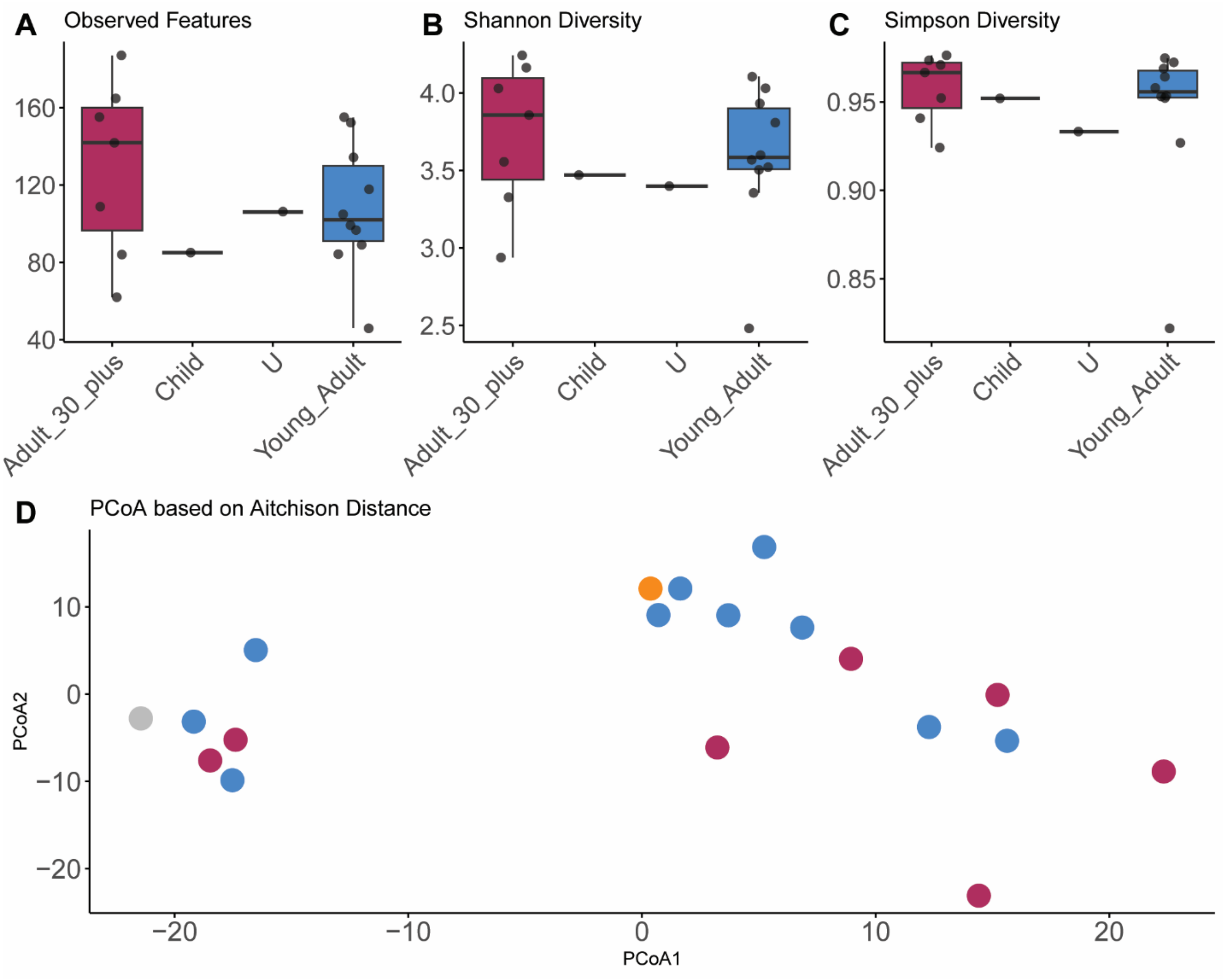

Figures S10 A-D. A) Observed features (ASVs) diversity , B) Shannon diversity, C) Simpson diversity and D) PCoA for fecal samples at the species level based on the Greengenes2 database.

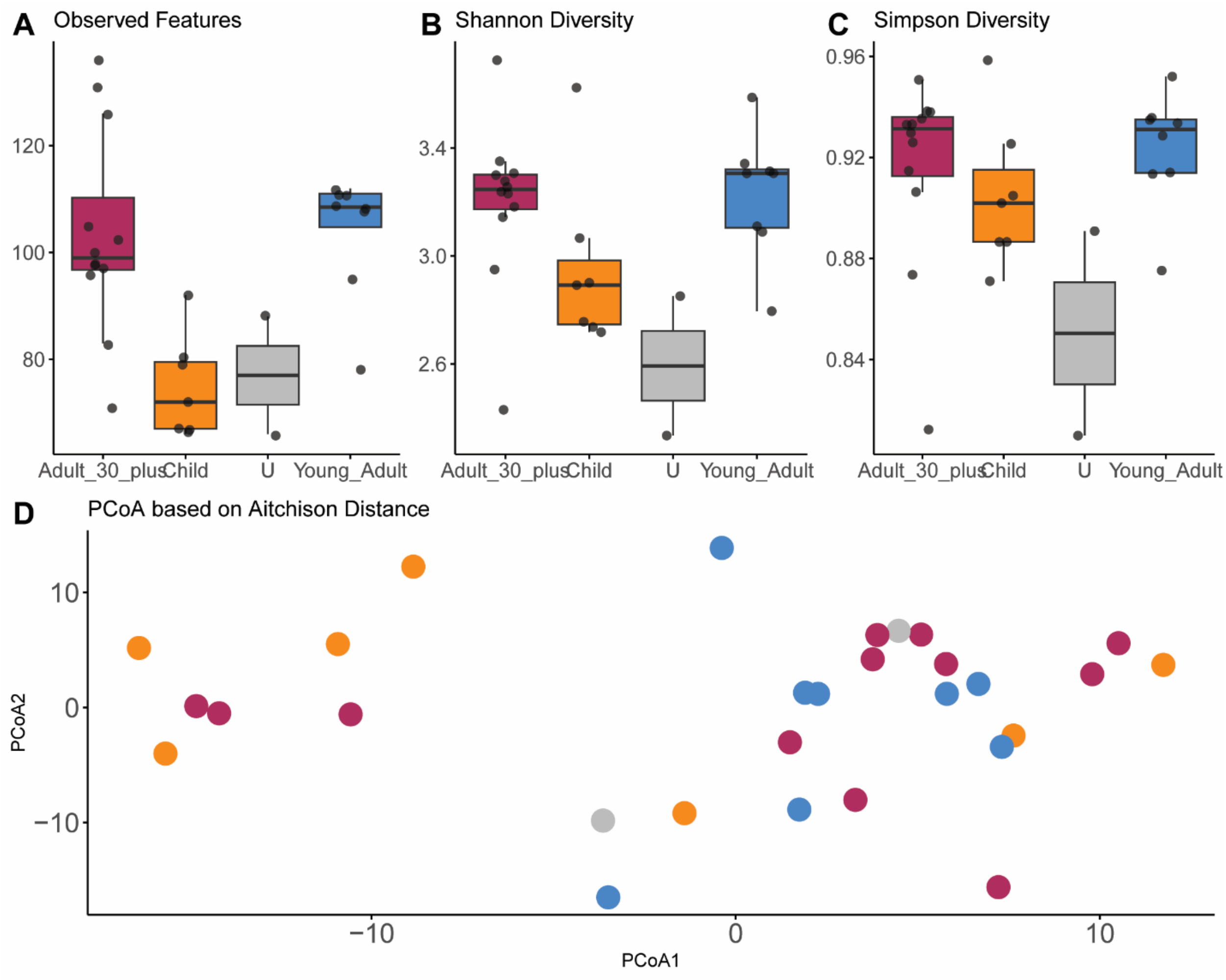

Figures S11 A-D. A) Observed features (ASVs) diversity , B) Shannon diversity, C) Simpson diversity and D) PCoA for saliva samples at the species level based on the Greengenes2 database.

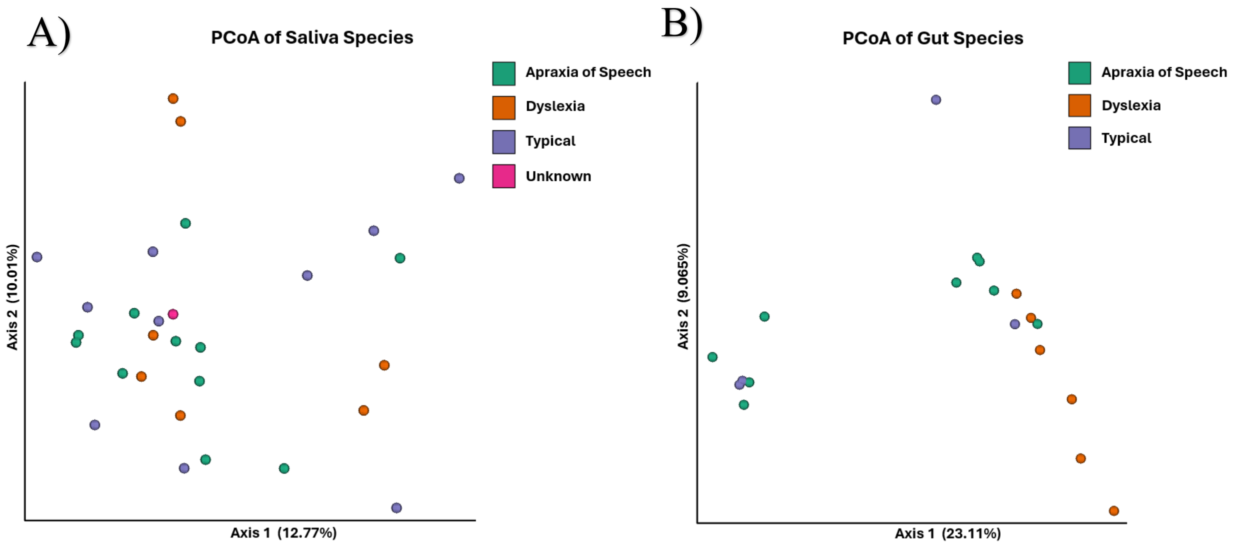

Figure S12. Beta diversity principal coordinate analysis (PCoA) plot of A) saliva and B) fecal samples aligned against the GSR database.

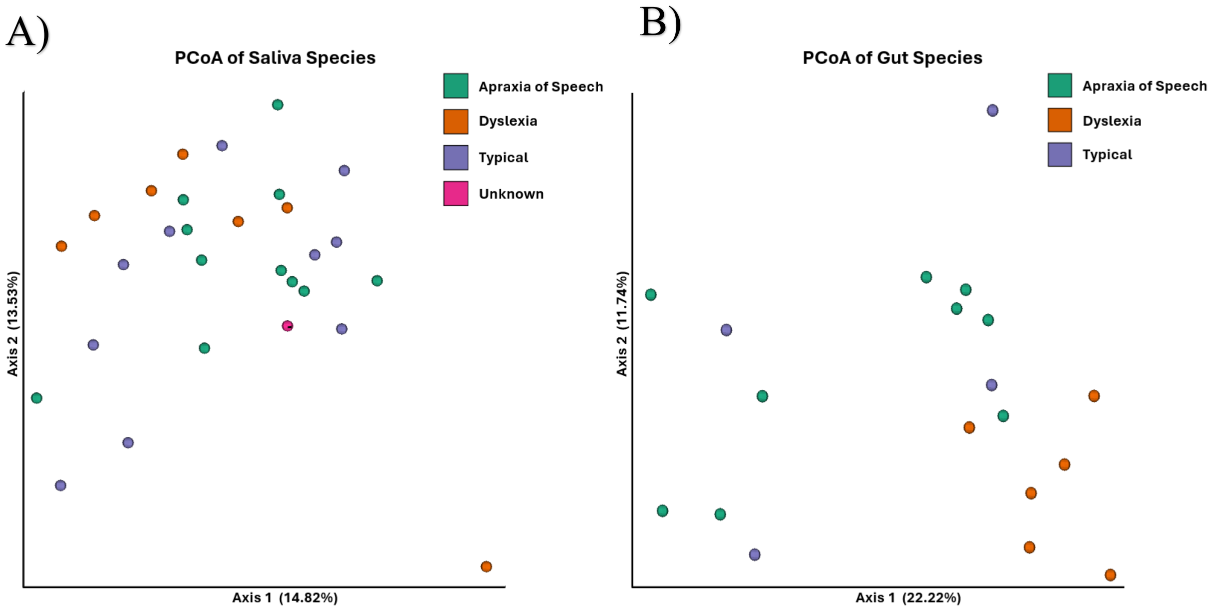

Figure S13. Beta diversity principal coordinate analysis (PCoA) plot of A) saliva and B) fecal samples aligned against the MIMt database

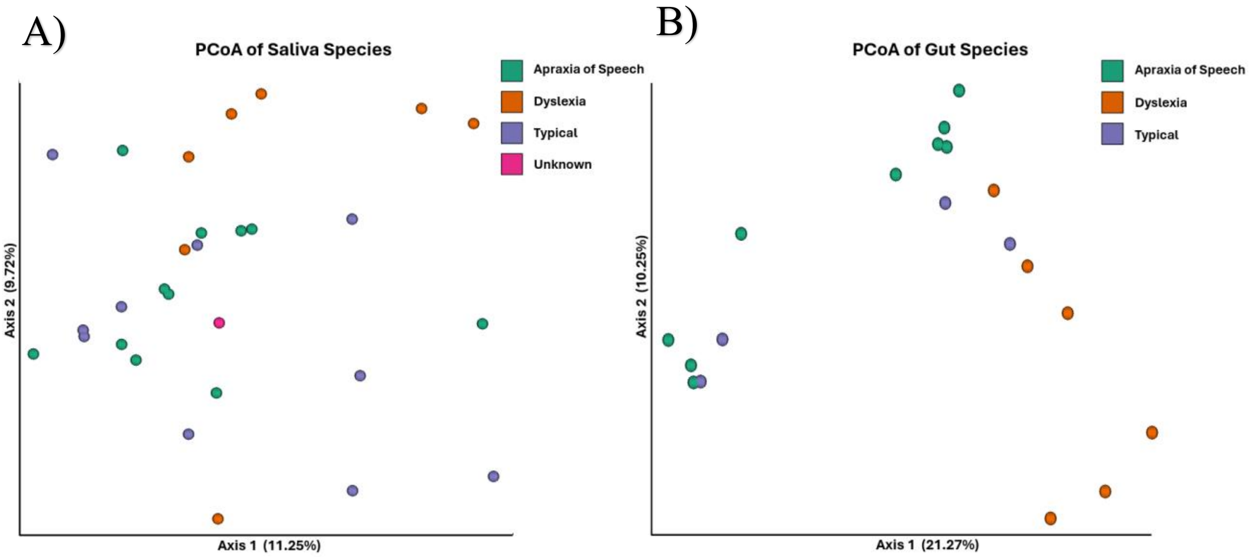

Figure S14. Beta diversity principal coordinate analysis (PCoA) plot of A) saliva and B) fecal samples aligned against the Silva-138 database.

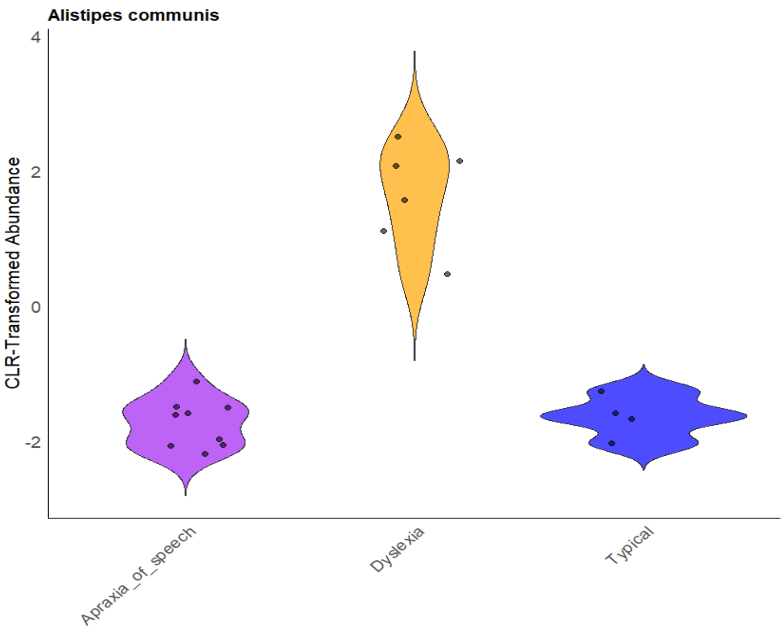

Figure S15. Significant results from MaAsLin2 differential abundance analysis performed on the fecal samples aligned against the GSR database

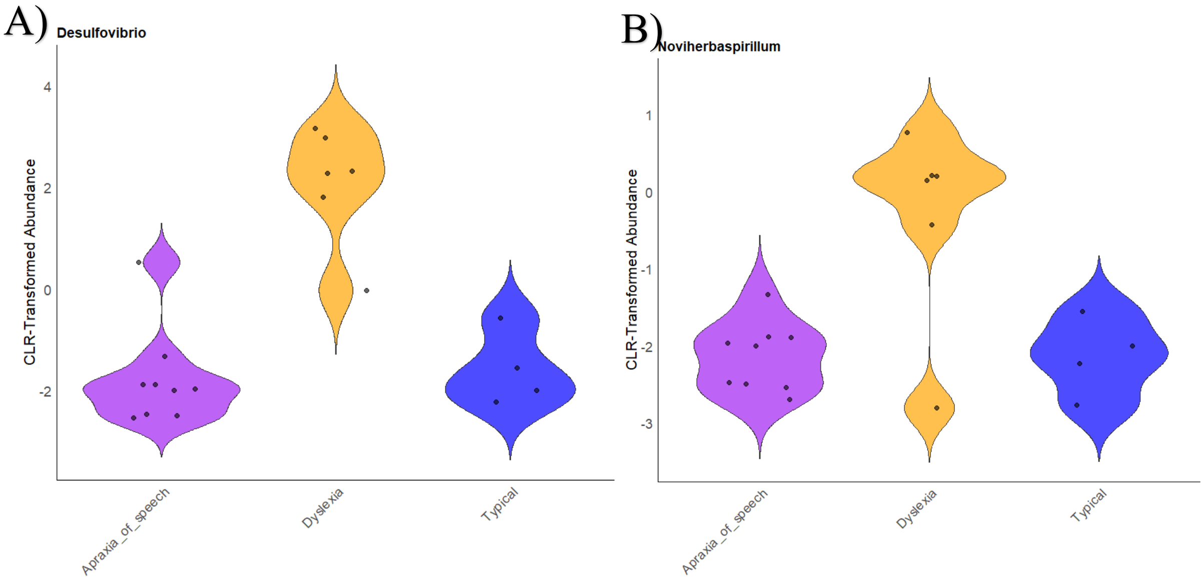

352

353 Figure S16 A-B. Significant results from MaAsLin2 differential abundance analysis performed on
354 the fecal samples aligned against the MIMt database.

355

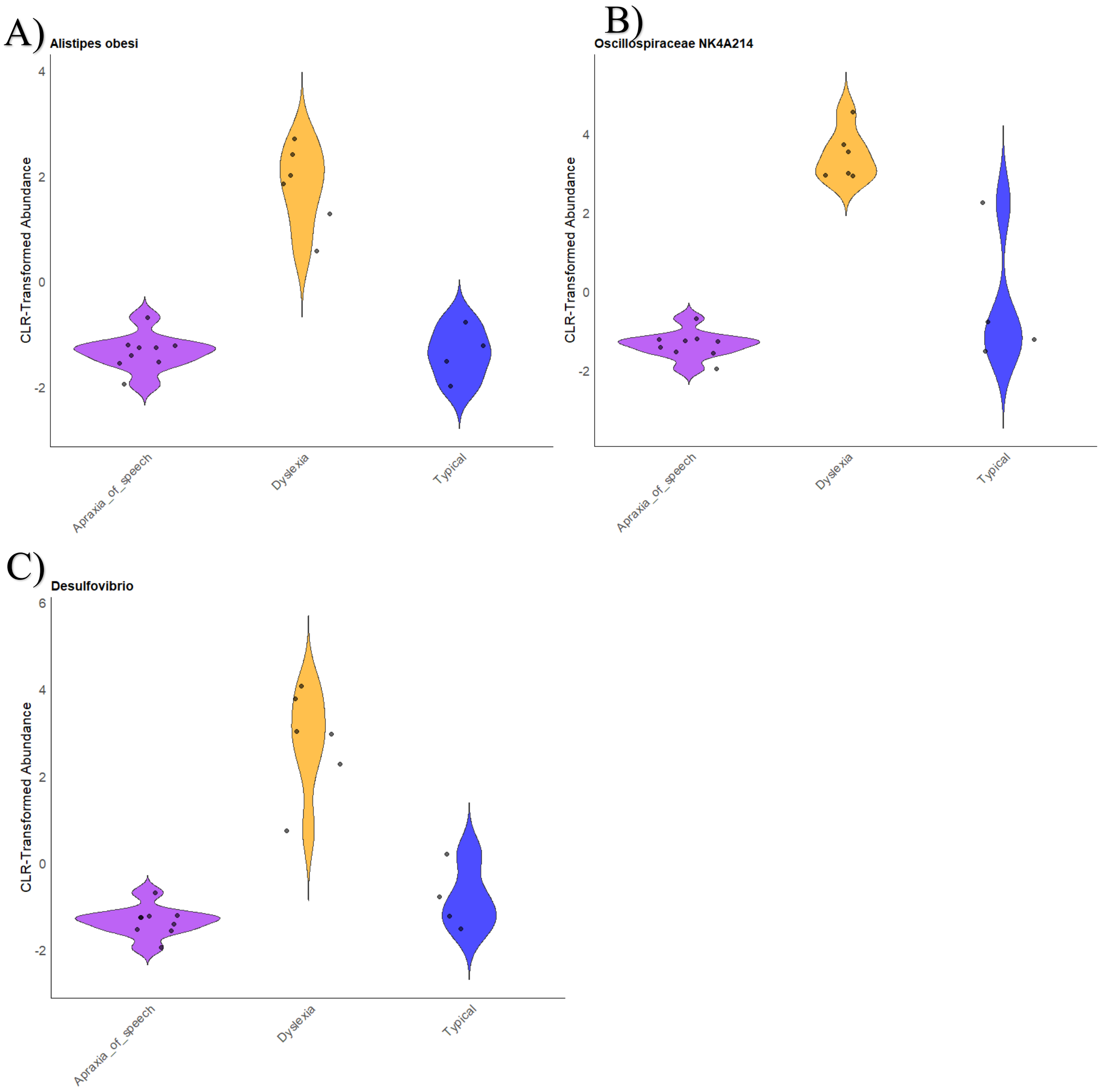

Figures S17 A-C. Significant results from MaAsLin2 differential abundance analysis performed on the fecal samples aligned against the SILVA-138 database.

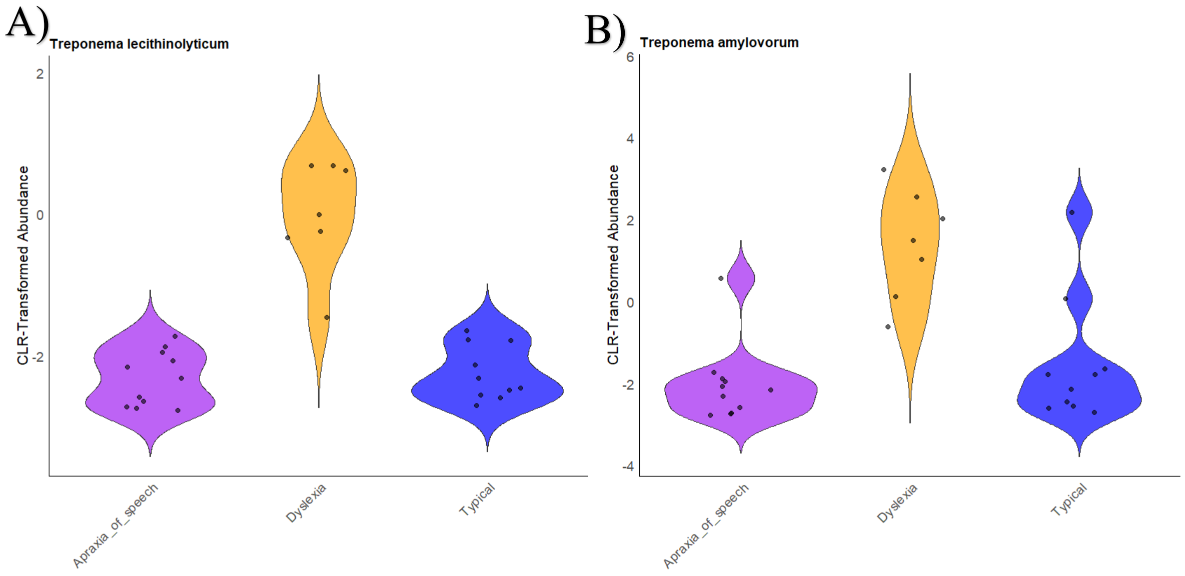

Figures S18 A-B. Significant results from MaAsLin2 differential abundance analysis performed on the saliva samples aligned against the GSR database.

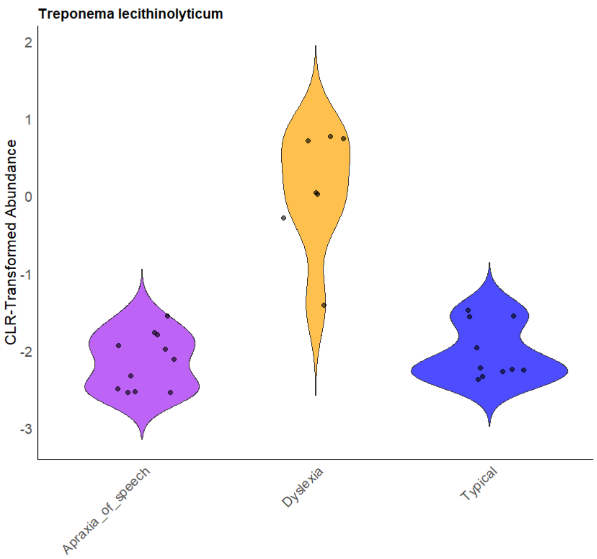

Figure S19. Significant results from MaAsLin2 differential abundance analysis performed on the saliva samples aligned against the SILVA-138 database.

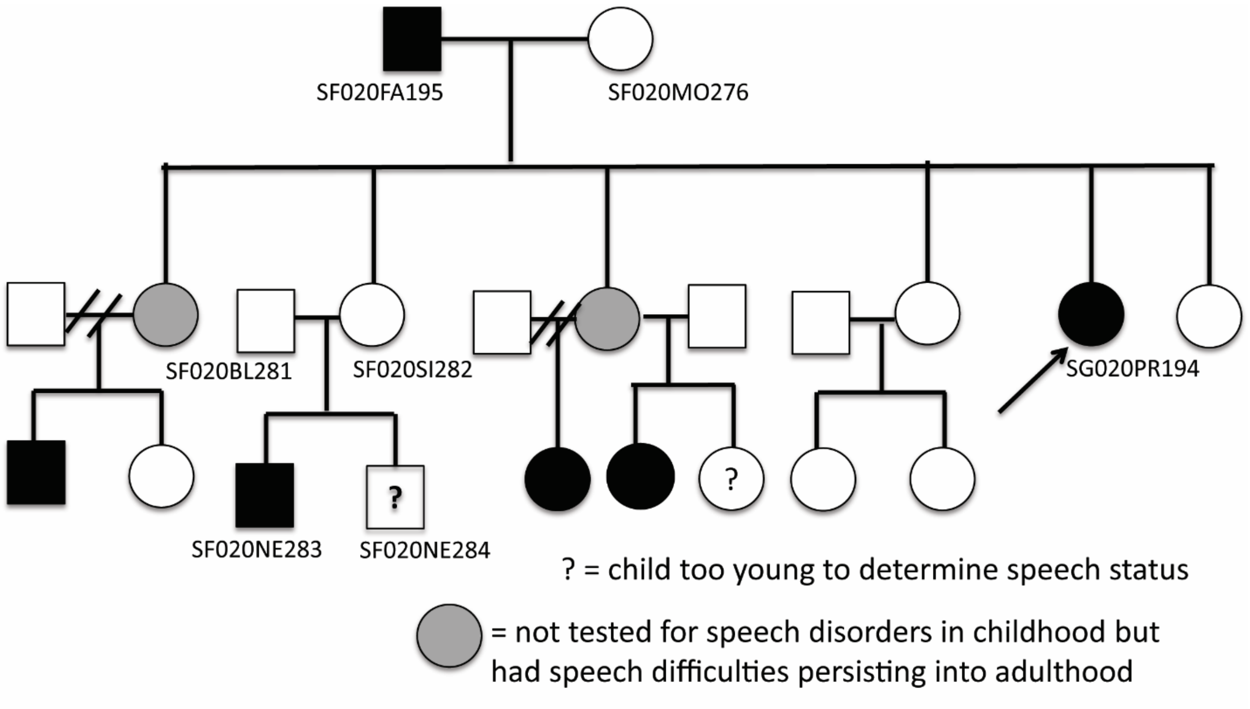

Figure S20. Pedigree illustrating the inheritance pattern of Childhood Apraxia of Speech within family SF020. Filled shapes (black) represent indivdiuals diagnosed with CAS. Gray circles indicate individuals who exhibited speech difficulties but did not receive a formal diagnosis. Shapes marked with question marks denote children who were too young to undergo diagnostic evaluation at the time of assessment.

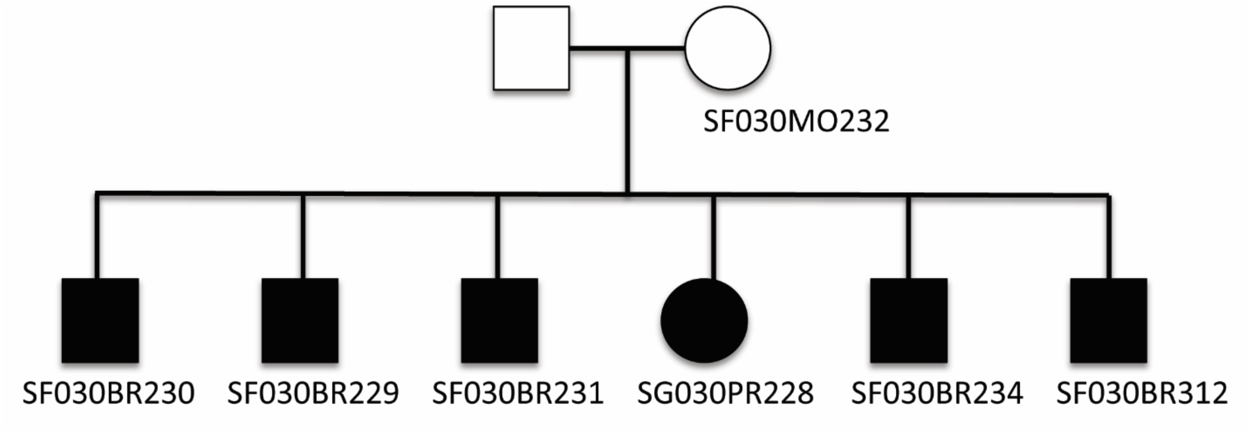

Figure S21. Pedigree illustrating the inheritance pattern of Childhood Apraxia of Speech within family SF030. Filled shapes (black) represent individuals diagnosed with CAS.

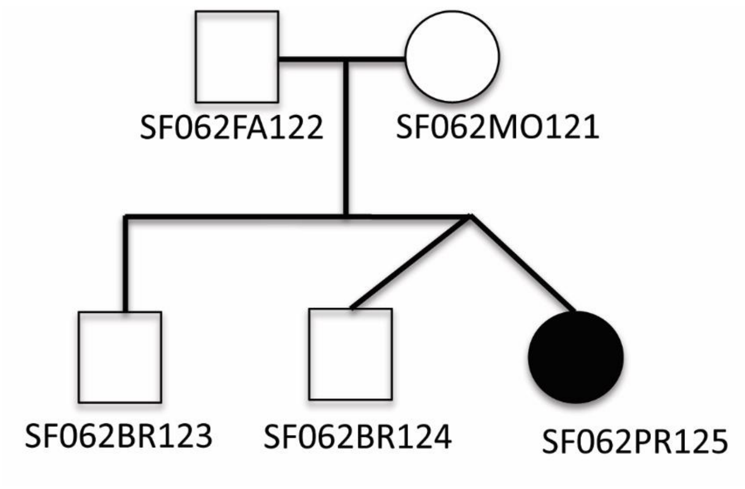

Figure S22. Pedigree illustrating the inheritance pattern of Childhood Apraxia of Speech within family SF062. Filled shapes (black) represent individuals diagnosed with CAS.

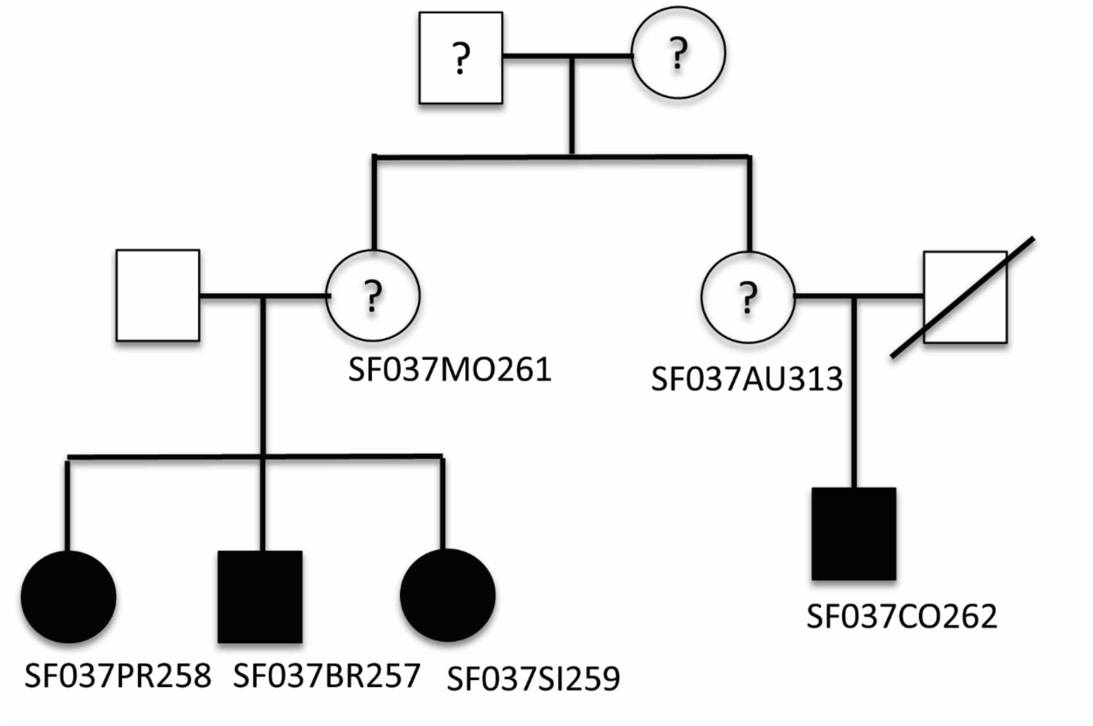

Figure S23. Pedigree illustrating the inheritance pattern of Childhood Apraxia of Speech within family SF020. Filled shapes (black) represent individuals diagnosed with CAS. Shapes marked with question marks denote children who were too young to undergo diagnostic evaluation at the time of assessment.

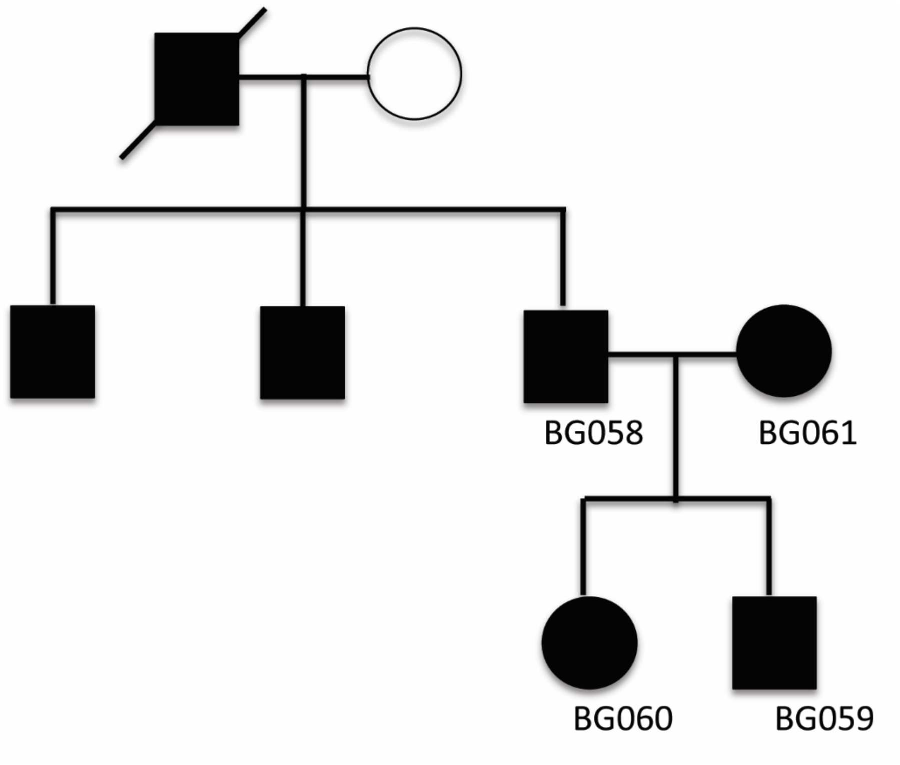

Figure S24. Pedigree illustrates the inheritance pattern of dyslexia within a family. Filled shapes (black) represent individuals diagnosed with dyslexia.

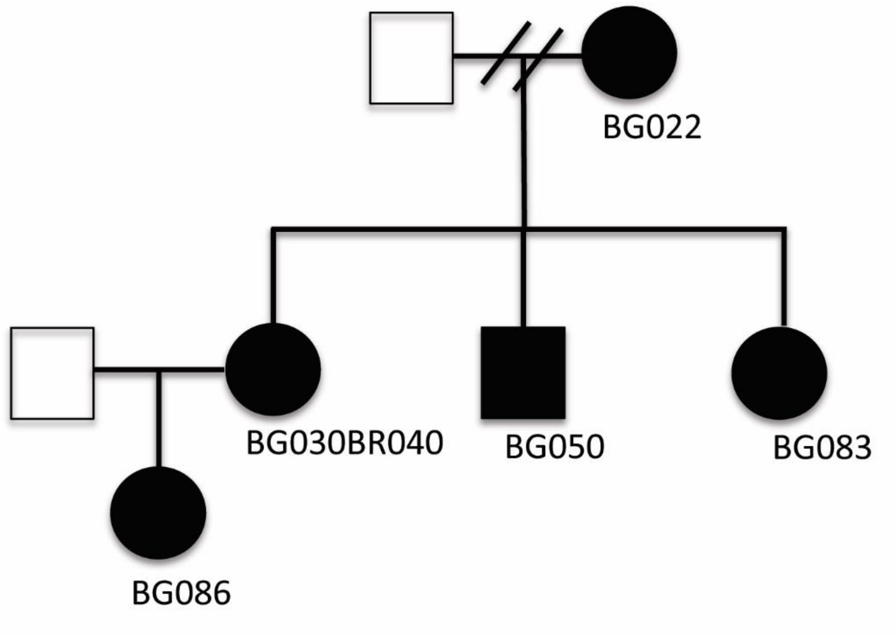

Figure S25. Pedigree illustrates the inheritance pattern of dyslexia within a family. Filled shapes (black) represent individuals diagnosed with dyslexia.

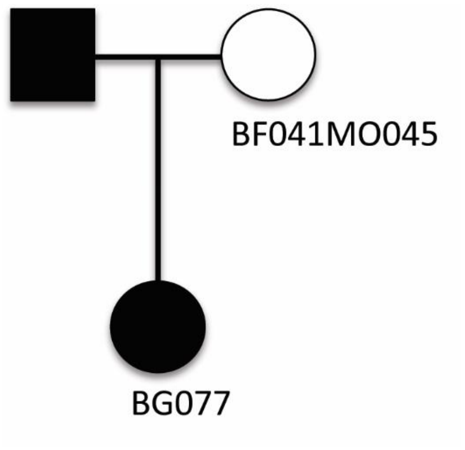

Figure S26. Pedigree illustrates the inheritance pattern of dyslexia within a family. Filled shapes (black) represent individuals diagnosed with dyslexia.

References

1. Miller GJ, Lewis BA. Reading Skills in Children With Suspected Childhood Apraxia of Speech and Children With Reading Disorders: Same or Different? Language, Speech, and Hearing Services in Schools. 2022;53: 985–1005. doi:10.1044/2022_LSHSS-21-00149

2. Murray E, McCabe P, Heard R, Ballard KJ. Differential Diagnosis of Children with Suspected Childhood Apraxia of Speech. Journal of Speech, Language, and Hearing Research. 2015;58: 43–60. doi:10.1044/2014_JSLHR-S-12-0358

3. Snowling MJ, Gallagher A, Frith U. Family Risk of Dyslexia Is Continuous: Individual Differences in the Precursors of Reading Skill. Child Development. 2003;74: 358–373. doi:10.1111/1467-8624.7402003

4. Thompson PA, Hulme C, Nash HM, Gooch D, Hayiou-Thomas E, Snowling MJ. Developmental dyslexia: predicting individual risk. Journal of Child Psychology and Psychiatry. 2015;56: 976–987. doi:10.1111/jcpp.12412

5. Paulesu E, Démonet J-F, Fazio F, McCrory E, Chanoine V, Brunswick N, et al. Dyslexia: Cultural Diversity and Biological Unity. Science. 2001;291: 2165–2167. doi:10.1126/science.1057179

6. Anastasopoulou I, Cheyne DO, Lieshout P van, Wilson PH, Ballard KJ, Johnson BW. A novel candidate neuromarker of central motor dysfunction in childhood apraxia of speech. J Neurosci. 2025 [cited 29 Apr 2025]. doi:10.1523/JNEUROSCI.1471-24.2025

7. Button L, Peter ,Beate, Stoel-Gammon ,Carol, and Raskind WH. Associations among measures of sequential processing in motor and linguistics tasks in adults with and without a family history of childhood apraxia of speech: A replication study. Clinical Linguistics & Phonetics. 2013;27: 192–212. doi:10.3109/02699206.2012.744097

8. Peter B, Bruce ,Laurel, Raaz ,Caitlin, Williams ,Emma, Pfeiffer ,Allie, and Rogalsky C. Comparing global motor characteristics in children and adults with childhood apraxia of speech to a cerebellar stroke patient: evidence for the cerebellar hypothesis in a developmental motor speech disorder. Clinical Linguistics & Phonetics. 2021;35: 368–392. doi:10.1080/02699206.2020.1861103

9. Peter B, Lancaster ,Hope, Vose ,Caitlin, Middleton ,Kyle, and Stoel-Gammon C. Sequential processing deficit as a shared persisting biomarker in dyslexia and childhood apraxia of speech. Clinical Linguistics & Phonetics. 2018;32: 316–346. doi:10.1080/02699206.2017.1375560

10. Peter B, Lancaster H, Vose C, Fares A, Schrauwen I, Huentelman M. Two unrelated children with overlapping 6q25.3 deletions, motor speech disorders, and language delays. American Journal of Medical Genetics Part A. 2017;173: 2659–2669. doi:10.1002/ajmg.a.38385

11. Peter B, Wijsman EM, Jr AQN, Genomics U of WC for M, Matsushita MM, Chapman KL, et al. Genetic Candidate Variants in Two Multigenerational Families with Childhood Apraxia of Speech. PLOS ONE. 2016;11: e0153864. doi:10.1371/journal.pone.0153864

12. Peter B, Button ,Le, Stoel-Gammon ,Carol, Chapman ,Kathy, and Raskind WH. Deficits in sequential processing manifest in motor and linguistic tasks in a multigenerational family with childhood apraxia of speech. Clinical Linguistics & Phonetics. 2013;27: 163–191. doi:10.3109/02699206.2012.736011

13. Koziol LF, Budding D, Andreasen N, D'Arrigo S, Bulgheroni S, Imamizu H, et al. Consensus Paper: The Cerebellum's Role in Movement and Cognition. Cerebellum. 2014;13: 151–177. doi:10.1007/s12311-013-0511-x

14. Manto M, Bower JM, Conforto AB, Delgado-García JM, da Guarda SNF, Gerwig M, et al. Consensus Paper: Roles of the Cerebellum in Motor Control—The Diversity of Ideas on Cerebellar Involvement in Movement. Cerebellum. 2012;11: 457–487. doi:10.1007/s12311-011-0331-9

15. Mariën P, van Dun K, Verhoeven J. Cerebellum and Apraxia. Cerebellum. 2015;14: 39–42. doi:10.1007/s12311-014-0620-1

16. Miller GJ, Lewis B, Benchek P, Freebairn L, Tag J, Budge K, et al. Reading Outcomes for Individuals With Histories of Suspected Childhood Apraxia of Speech. American Journal of Speech-Language Pathology. 2019;28: 1432–1447. doi:10.1044/2019_AJSLP-18-0132

17. Baker E, McLeod S. Evidence-Based Practice for Children With Speech Sound Disorders: Part 1 Narrative Review. Language, Speech, and Hearing Services in Schools. 2011;42: 102–139. doi:10.1044/0161-1461(2010/09-0075)

18. Reis A, Araújo S, Morais IS, Faísca L. Reading and reading-related skills in adults with dyslexia from different orthographic systems: a review and meta-analysis. Ann of Dyslexia. 2020;70: 339–368. doi:10.1007/s11881-020-00205-x

19. Almeida A, Mitchell AL, Tarkowska A, Finn RD. Benchmarking taxonomic assignments based on 16S rRNA gene profiling of the microbiota from commonly sampled environments. GigaScience. 2018;7: giy054. doi:10.1093/gigascience/giy054

20. Balvočiūtė M, Huson DH. SILVA, RDP, Greengenes, NCBI and OTT — how do these taxonomies compare? BMC Genomics. 2017;18: 114. doi:10.1186/s12864-017-3501-4

21. McDonald D, Jiang Y, Balaban M, Cantrell K, Zhu Q, Gonzalez A, et al. Greengenes2 unifies microbial data in a single reference tree. Nature biotechnology. 2024;42: 715–718.

22. Molano L-AG, Vega-Abellaneda S, Manichanh C. GSR-DB: a manually curated and optimized taxonomical database for 16S rRNA amplicon analysis. mSystems. 2024;9: e00950-23. doi:10.1128/msystems.00950-23

23. Cabezas MP, Fonseca NA, Muñoz-Mérida A. MIMt: a curated 16S rRNA reference database with less redundancy and higher accuracy at species-level identification. Environmental Microbiome. 2024;19: 1–13. doi:10.1186/s40793-024-00634-w

24. Hiergeist A, Ruelle J, Emler S, Gessner A. Reliability of species detection in 16S microbiome analysis: Comparison of five widely used pipelines and recommendations for a more standardized approach. PLOS ONE. 2023;18: e0280870. doi:10.1371/journal.pone.0280870

25. Pruesse E, Quast C, Knittel K, Fuchs BM, Ludwig W, Peplies J, et al. SILVA: A comprehensive online resource for quality checked and aligned ribosomal RNA sequence data compatible with ARB. Nucleic Acids Research. 2007;35: 7188–7196. doi:10.1093/nar/gkm864

26. Dixit K, Davray D, Chaudhari D, Kadam P, Kshirsagar R, Shouche Y, et al. Benchmarking of 16S rRNA gene databases using known strain sequences. Bioinformation. 2021;17: 377–391. doi:10.6026/97320630017377

27. Jovel J, Patterson J, Wang W, Hotte N, O'Keefe S, Mitchel T, et al. Characterization of the Gut Microbiome Using 16S or Shotgun Metagenomics. Front Microbiol. 2016;7. doi:10.3389/fmicb.2016.00459

28. Lehr K, Oosterlinck B, Then CK, Gemmell MR, Gedgaudas R, Bornschein J, et al. Comparison of different microbiome analysis pipelines to validate their reproducibility of gastric mucosal microbiome composition. mSystems. 2025;10: e01358-24. doi:10.1128/msystems.01358-24

29. Nagai T, Shiba T, Komatsu K, Watanabe T, Nemoto T, Maekawa S, et al. Optimal 16S rRNA gene amplicon sequencing analysis for oral microbiota to avoid the potential bias introduced by trimming length, primer, and database. Microbiology Spectrum. 2024;12: e03512-23. doi:10.1128/spectrum.03512-23

30. Lobanov V, Gobet A, Joyce A. Ecosystem-specific microbiota and microbiome databases in the era of big data. Environmental Microbiome. 2022;17: 37. doi:10.1186/s40793-022-00433-1

31. Kool J, Tymchenko L, Shetty SA, Fuentes S. Reducing bias in microbiome research: Comparing methods from sample collection to sequencing. Front Microbiol. 2023;14. doi:10.3389/fmicb.2023.1094800

32. Wright SL. Archaeological dental calculus: A rich bioarchive for exploring Classical Antiquity through ancient DNA methods. Journal of Archaeological Science: Reports. 2025;62: 105038. doi:10.1016/j.jasrep.2025.105038

33. Martino C, Morton JT, Marotz CA, Thompson LR, Tripathi A, Knight R, et al. A novel sparse compositional technique reveals microbial perturbations. MSystems. 2019;4: 10–1128.

34. Gloor GB, Macklaim JM, Pawlowsky-Glahn V, Egozcue JJ. Microbiome Datasets Are Compositional: And This Is Not Optional. Front Microbiol. 2017;8. doi:10.3389/fmicb.2017.02224

35. Gloor GB, Wu JR, Pawlowsky-Glahn V, Egozcue JJ. It's all relative: analyzing microbiome data as compositions. Annals of Epidemiology. 2016;26: 322–329. doi:10.1016/j.annepidem.2016.03.003

36. Vázquez-Baeza Y, Pirrung M, Gonzalez A, Knight R. EMPeror: a tool for visualizing high-throughput microbial community data. GigaScience. 2013;2: 2047-217X-2–16. doi:10.1186/2047-217X-2-16

37. Anderson MJ. A new method for non-parametric multivariate analysis of variance. Austral ecology. 2001;26: 32–46.

38. Estaki M, Jiang L, Bokulich NA, McDonald D, González A, Kosciolek T, et al. QIIME 2 Enables Comprehensive End-to-End Analysis of Diverse Microbiome Data and Comparative Studies with Publicly Available Data. Current Protocols in Bioinformatics. 2020;70: e100. doi:10.1002/cpbi.100
