# Supplementary material for: Distinct gut and oral microbiome patterns associated with dyslexia in a family-based cohort: A preliminary exploratory study
Source: PLoS One. 2026 Jul 21;21(7):e0353463. doi: 10.1371/journal.pone.0353463 (PMC13387549; doi:10.1371/journal.pone.0353463)
Supplement: Attachment — Submitted filename: Reply-to-the-Editor-and-Reviewers.docx [file pone.0353463.s004.docx]

**Reply to the Editor and Reviewers**

We thank the Editor and reviewers for their thoughtful comments and constructive comments, which have greatly improved the clarity and rigor of our manuscript. We have carefully considered and addressed all comments point by point. Our detailed responses are provided below in **red,** and corresponding revisions have been made in the manuscript.

**When submitting your revision, we need you to address these additional requirements.**

**1.Please ensure that your manuscript meets PLOS ONE's style requirements, including those for file naming. The PLOS ONE style templates can be found at**

[**https://journals.plos.org/plosone/s/file?id=wjVg/PLOSOne_formatting_sample_main_body.pdf**](https://journals.plos.org/plosone/s/file?id=wjVg/PLOSOne_formatting_sample_main_body.pdf) **and**

[**https://journals.plos.org/plosone/s/file?id=ba62/PLOSOne_formatting_sample_title_authors_affiliations.pdf**](https://track.editorialmanager.com/CL0/https:%2F%2Fjournals.plos.org%2Fplosone%2Fs%2Ffile%3Fid=ba62%2FPLOSOne_formatting_sample_title_authors_affiliations.pdf/1/010f019c2171df8a-d62866bc-72d9-4dde-b7d6-cf0b5ffc2fd1-000000/V7sRfzdUaP50hOYxkZXNFTWKxJDzw4kYmp_4YCHh2kg=248)

**2. Please note that funding information should not appear in any section or other areas of your manuscript. We will only publish funding information present in the Funding Statement section of the online submission form. Please remove any funding-related text from the manuscript.**

**3. We note that the grant information you provided in the ‘Funding Information’ and ‘Financial Disclosure’ sections do not match.**

**When you resubmit, please ensure that you provide the correct grant numbers for the awards you received for your study in the ‘Funding Information’ section.**

Thank you for bringing this to our attention. We have made sure the grant numbers are reported.

**4. Thank you for stating the following financial disclosure:**

**“Microbiome analysis was supported by a seed grant awarded to B.P. from the Institute for Social Science Research (ISSR) at Arizona State University. The work of M. O. was supported by the National Institute of Diabetes and Digestive and Kidney Diseases of the National Institutes of Health under Award Number T32DK137525.”**

**Please state what role the funders took in the study.  If the funders had no role, please state: "The funders had no role in study design, data collection and analysis, decision to publish, or preparation of the manuscript."**

Thank you for this clarification. We have updated the text to address this concern.

**5. When completing the data availability statement of the submission form, you indicated that you will make your data available on acceptance. We strongly recommend all authors decide on a data sharing plan before acceptance, as the process can be lengthy and hold up publication timelines. Please note that, though access restrictions are acceptable now, your entire data will need to be made freely accessible if your manuscript is accepted for publication. This policy applies to all data except where public deposition would breach compliance with the protocol approved by your research ethics board. If you are unable to adhere to our open data policy, please kindly revise your statement to explain your reasoning and we will seek the editor's input on an exemption. Please be assured that, once you have provided your new statement, the assessment of your exemption will not hold up the peer review process.**

We have revised our data availability statement to state that our data is publicly available. Specifically, we state that all our data is on the NCBI SRA database, under the project number: PRJNA1246995. The SRA database is a commonly used repository for microbiome data to be deposited. We also provide our bioinformatic scripts on GitHub for reproducibility.

**6. Your ethics statement should only appear in the Methods section of your manuscript. If your ethics statement is written in any section besides the Methods, please delete it from any other section.**

We have ensured that the ethics statement is only in the Methods section (Lines 80-81).

**7. We note that Figure 1 in your submission contain copyrighted images. All PLOS content is published under the Creative Commons Attribution License (CC BY 4.0), which means that the manuscript, images, and Supporting Information files will be freely available online, and any third party is permitted to access, download, copy, distribute, and use these materials in any way, even commercially, with proper attribution. For more information, see our copyright guidelines:** [**http://journals.plos.org/plosone/s/licenses-and-copyright**](http://journals.plos.org/plosone/s/licenses-and-copyright)**.**

**We require you to either (1) present written permission from the copyright holder to publish these figures specifically under the CC BY 4.0 license, or (2) remove the figures from your submission:**

**1. You may seek permission from the original copyright holder of Figure 1 to publish the content specifically under the CC BY 4.0 license.**

**We recommend that you contact the original copyright holder with the Content Permission Form (**[**http://journals.plos.org/plosone/s/file?id=7c09/content-permission-form.pdf**](http://journals.plos.org/plosone/s/file?id=7c09/content-permission-form.pdf)**) and the following text:**

**“I request permission for the open-access journal PLOS ONE to publish XXX under the Creative Commons Attribution License (CCAL) CC BY 4.0 (**[**http://creativecommons.org/licenses/by/4.0/**](http://creativecommons.org/licenses/by/4.0/)**). Please be aware that this license allows unrestricted use and distribution, even commercially, by third parties. Please reply and provide explicit written permission to publish XXX under a CC BY license and complete the attached form.”**

**Please upload the completed Content Permission Form or other proof of granted permissions as an "Other" file with your submission.**

**In the figure caption of the copyrighted figure, please include the following text: “Reprinted from [ref] under a CC BY license, with permission from [name of publisher], original copyright [original copyright year].”**

Thank you for bringing this to our attention. To address this concern, we have updated our Figure 1 to ensure that it has no copyright images (*i.e.,* we have removed all figures that were downloaded from BioRender). The updated figure also provides a clearer picture about the study design.

**2. If you are unable to obtain permission from the original copyright holder to publish these figures under the CC BY 4.0 license or if the copyright holder’s requirements are incompatible with the CC BY 4.0 license, please either i) remove the figure or ii) supply a replacement figure that complies with the CC BY 4.0 license. Please check copyright information on all replacement figures and update the figure caption with source information. If applicable, please specify in the figure caption text when a figure is similar but not identical to the original image and is therefore for illustrative purposes only.**

**8. Please remove your figures from within your manuscript file, leaving only the individual TIFF/EPS image files, uploaded separately. These will be automatically included in the reviewers’ PDF.**

Thank you for this comment. We have removed the figures from the manuscript.

**9. We note that there is identifying data in the Supporting Information file “SupplementalTables.xlsx”. Due to the inclusion of these potentially identifying data, we have removed this file from your file inventory. Prior to sharing human research participant data, authors should consult with an ethics committee to ensure data are shared in accordance with participant consent and all applicable local laws.**

**Data sharing should never compromise participant privacy. It is therefore not appropriate to publicly share personally identifiable data on human research participants. The following are examples of data that should not be shared:**

**-Name, initials, physical address**

**-Ages more specific than whole numbers**

**-Internet protocol (IP) address**

**-Specific dates (birth dates, death dates, examination dates, etc.)**

**-Contact information such as phone number or email address**

**-Location data**

**-ID numbers that seem specific (long numbers, include initials, titled “Hospital ID”) rather than random (small numbers in numerical order)**

**Data that are not directly identifying may also be inappropriate to share, as in combination they can become identifying. For example, data collected from a small group of participants, vulnerable populations, or private groups should not be shared if they involve indirect identifiers (such as sex, ethnicity, location, etc.) that may risk the identification of study participants.**

**Additional guidance on preparing raw data for publication can be found in our Data Policy (**[**https://journals.plos.org/plosone/s/data-availability#loc-human-research-participant-data-and-other-sensitive-data**](https://journals.plos.org/plosone/s/data-availability#loc-human-research-participant-data-and-other-sensitive-data)**) and in the following article:** [**http://www.bmj.com/content/340/bmj.c181.long**](http://www.bmj.com/content/340/bmj.c181.long)**.**

**Please remove or anonymize all personal information (<specific identifying information in file to be removed>), ensure that the data shared are in accordance with participant consent, and re-upload a fully anonymized data set. Please note that spreadsheet columns with personal information must be removed and not hidden as all hidden columns will appear in the published file.**

Thank you for bringing this to our attention. We have removed all potentially identifying data. All metadata columns (*i.e.,* anonymized SampleID, Family_ID, SampleType, Gender, Ethnicity, Phenotype, and Relationship to Proband) have been approved by the IRB.

**10. If the reviewer comments include a recommendation to cite specific previously published works, please review and evaluate these publications to determine whether they are relevant and should be cited. There is no requirement to cite these works unless the editor has indicated otherwise. 

Additional Editor Comments:

General comments:
Kindly, remove the repetitive speech in the manuscript about the sample size and the limitations. Only include it in the limitation and conclusion section.**
We thank the editor for this suggestion. We have revised the manuscript to remove repetitive references to sample size and study limitations from the Results section. These considerations are now discussed exclusively in the Discussion and reiterated in the Conclusion to ensure clarity without redundancy.

**Abstract:
Kindly, rewrite the abstract making it structured.
The aim of the study needs to be clarified in the abstract**
We have rewritten the abstract in a structured format and explicitly clarified the aim of the study, stating that the objective was to determine whether fecal and saliva microbiome features are associated with dyslexia or childhood apraxia of speech (CAS).

**Introduction and rational
Some issues needs clarification in the introduction and rational section
Why the authors compared the dyslexia with the CAS?- Kindly clarify your rational**
We thank the editor for this question. In the Introduction, we added the following explanation for selecting CAS and dyslexia for this study: **“**[6,7]. Although each [CAS and dyslexia] is distinct in their core observable characteristics (*i.e.,* phenotypes) and clinical management, both are frequently comorbid [8] and share some biomarkers for motor discoordination and difficulties with sequential information processing [9].”

In addition, in the third paragraph of the Introduction, we added, “Yet, they [CAS and dyslexia] may share overlapping neurobiological vulnerabilities. This makes them a useful comparative framework for exploring biological factors that may be shared across language impairments versus those that are disorder specific. One such factor may be the microbiome, as emerging evidence indicates that microbial communities involved along the oral-gut-brain axis can influence neurodevelopment, cognition, and behavior through immune, metabolic, and neuroactive signaling pathways [21–23].”

In short, this comparative framework allows us to explore whether microbiome associations are disorder-specific or shared across language impairments within a family-based design.

**Methods:
Why the number of saliva samples is different from fecal samples? Have some patients refused to give fecal samples? Were some samples spoilt?- Kindly, clarify these points in the methods section**

We thank the reviewer for this clarification request. We added an explanation in the Methods section: “In total, 29 saliva samples and 19 fecal samples were collected. The difference in sample numbers reflect participant choice to provide both sample types or saliva only. Some individuals elected to provide saliva samples only due to convenience and easy of collection. For all 19 fecal samples, a matched saliva sample from the same participant was also available.”

**Did you get the saliva and fecal samples from the same patient?**

Yes. Everyone who provided a fecal sample also provided a saliva sample, consistent with the explanation we added for the difference in sample numbers.

**The authors mentioned the categorization of patients including undetermined phenotyping and neurotypical. Do the authors mean that 18 individual have symptoms and the others did not have?**

We thank the editor for this clarification request. Yes, this is correct. Table 1 shows the breakdown of participants with dyslexia (7), with CAS (11), neither (11), or unknown (1). We added this clarification to the text.

**Could you clarify this point more for the reader in the manuscript?
The definitions of phenotype, neurotypical and alpha and beta diversity is better to be included to make it easier for the reader to catch up the meanings.**

We have revised the manuscript to improve clarity for readers. Definitions of participant phenotype and neurotypical status have been added to the Introduction. Moreover, we introduced the term phenotype as observable characteristic. We added the term “unaffected” to the term “neurotypical” for clarity. The definitions and operationalization of alpha and beta diversity metrics have been added to the Methods section.

**Results:
The results contain parts that looks like discussion. The standard is to display the results with a short comment about the trend if needed to express the meaning of the results. After that, you can discuss the results in details and compare with others using supporting references. Therefore, kindly, rewrite the result section based on these recommendations and omit the repeated parts.**

We thank the editor for this helpful recommendation. We have revised the Results section to focus strictly on the presentation of findings, with only brief descriptive statements included where necessary to convey observed trends. Interpretive language, literature comparisons, and repeated discussion of limitations have been removed from the Results and consolidated into the Discussion section. Redundant content has also been removed to improve clarity and conciseness.

**The authors mentioned the mechanistic pathway results. Kindly, mention the importance of this mechanistic pathway briefly in the introduction section and add a sentence that reveals that you will assess it in the corresponding part in the methods section.**

We thank the reviewer for this helpful suggestion. We have revised the Introduction to briefly describe the relevance of microbial metabolic pathways implicated in neuroactive signaling and sulfur metabolism and to clarify their potential importance in the context of the oral-gut-brain axis (L65-67).  **Limitation and conclusion:
Thanks for including the limitations and discussing them explicitly.
Add the inter-sex variability and ethnic variability to the limitations
Make the conclusion more concise and avoid repetition. Although the study is novel and important, Avoid speech that is overestimating of the results importance.**

We thank the reviewer for this helpful feedback. We have revised the Limitations section to explicitly include inter-sex variability and the predominantly White composition of the cohort as additional factors that may limit generalizability.

We have also revised the Conclusion to improve concision, remove repetitive statements, and ensure that the strength of the language appropriately reflects the exploratory nature of the study. Specifically, we avoided wording that could be interpreted as overstating the importance or immediate implications of the findings, while retaining the novelty and relevance of the work.  **Reviewers' comments:

Reviewer's Responses to Questions**

**Reviewer #1:**

**Major Comments
1. Clarify the Study Design and Cohort Composition

The abstract mentions a “family-based cohort,” but the relationships between dyslexia, CAS, and neurotypical participants are not explicitly stated.
Suggestion: Specify how many individuals belonged to each group and whether participants with dyslexia and CAS were from the same families or separate families.**
We have revised the abstract to explicitly state the number of participants in each phenotype group and to clarify that individuals with dyslexia and CAS were drawn from separate families, with neurotypical relatives serving as within-family controls. We also added details regarding the composition of all participating families and the type of sample (saliva or both saliva and fecal) available for each.

**2. Distinguish Between Dyslexia and CAS Groups More Clearly

The study groups appear uneven (fecal n=19, saliva n=29), making it unclear how many samples belonged to each diagnostic category.
Suggestion: Provide a brief breakdown (e.g., “X dyslexia, Y CAS, Z neurotypical”).**
Thank you for this suggestion. We have the total number of individuals in the study as well as the number of participants per phenotype described on lines 84-85. Our detailed description in the Methods section breaks down the number of families and which family members were diagnosed with either of the two conditions.

**3. Interpretation of Findings Needs More Caution

The enrichment of Alistipes A 871400 communis is interesting, but the text risks implying a causal or mechanistic link.
Suggestion: Reinforce that the associations are correlative and the sample size is small.**
We appreciate the reviewer’s suggestion. In the discussion, we have reinforced the idea that the associations are correlative and the sample size is small by stating:

“However, causality cannot be inferred because of the cross-sectional nature of the study, coupled with a modest sample size. Further research incorporating larger, independent cohorts and integrated multi-omic approaches, including shotgun metagenomics, metabolomics, and other functional profiling methods, will be needed to understand whether this taxon plays an active role in neurodevelopmental processes, reflects downstream consequences of shared environmental or dietary factors, or represents a correlated but unrelated signal.” (L-332-338).

**4. More Detail About the Analytical Methods

Many tools and databases are listed, but the abstract does not explain why multiple databases were used or whether consistency across them was evaluated.
Suggestion: Briefly mention whether the enrichment or diversity patterns were robust across databases.**

Thank you for bringing this concern to our attention. While we had described why multiple databases were used in the Appendix, we have updated the Methods section to explain why we use multiple databases and why we chose to present the results based on the taxonomic profiles using the Greengenes2 database.

**5. Clarify the Saliva vs. Fecal Microbiome Findings

The abstract notes differences in fecal microbiome profiles but is vague regarding saliva findings.
Suggestion: Clarify whether saliva microbiomes showed group-level differences or not.**
Thank you for bringing this to our attention. We have updated the text in the abstract to indicate that individuals with dyslexia also exhibit distinct saliva microbiome signatures compared to neurotypical family members, as well as individuals with CAS histories.

**Minor Comments
6. Precision in Terminology

“Participants with CAS histories” is unclear—does this mean persistent CAS, resolved CAS, or a history of speech delay?
Suggestion: Define the diagnostic category more precisely.**

We thank the reviewer for this helpful clarification request. "CAS histories" refers to a prior clinical diagnosis of childhood apraxia of speech or a self-reported history (L100-112).

**7. Improve Flow and Readability

The abstract is dense with methods, abbreviations, and technical references.
Suggestion: Streamline by focusing on the most important methodological points and moving details (e.g., specific database names) to methods in the full paper.**

We thank the reviewer for this helpful suggestion. We have revised the abstract to streamline methodological detail, focusing on the most essential aspects of the study design and analyses while removing technical specifics. Detailed methodological information has been retained in the Methods section of the full manuscript to improve readability and accessibility of the abstract.  **8. Grammar / Wording Adjustments

“remain underexplored” → “remains underexplored” (subject: microbiome).**

We have changed the text in the abstract to “remains underexplored…” **“patterns compared to neurotypical family members and participants with CAS histories” — the comparison could be clearer if rephrased as two explicit comparisons.

9. Consider Briefly Describing Functional Results

PICRUSt2 analysis is mentioned, but no functional findings are described.
Suggestion: Add 1–2 words on whether functional predictions aligned with taxonomic differences.**

We thank the reviewer for this helpful suggestion. We have revised the abstract to explicitly describe the PICRUSt2 functional predictions (L31-32), noting that dyslexia-associated functional pathways were detected in the fecal microbiome but not in saliva, thereby clarifying the relationship between functional predictions and observed taxonomic differences.

**Reviewer #2:**

**Thank you for the opportunity to review this manuscript.

I have reviewed the manuscript entitled “Distinct Gut and Oral Microbiome Patterns Associated with Dyslexia in a Family-Based Cohort.” This is an exploratory study that addresses an interesting question using paired fecal and saliva 16S rRNA (V4) data. The overall concept is timely, and the hypothesis-generating framing is appropriate. However, for the work to be publishable as a technically robust exploratory report, several sensitivity analyses and methodological clarifications are needed primarily because (i) there appears to be substantial imbalance in age and collection site across comparison groups, and (ii) the family-based sampling introduces non-independence that must be accounted for in inferential analyses.

In my view, the key requirements are:
1. Explicitly addressing age/site confounding;
2. Accounting for clustering at the family/household level;**

**3. Tempering species-level interpretations from 16S V4 ad clarifying classifier training;**

**4. Expanding reporting on negative controls/contamination and QC;
5. Ensuring metadata availability is sufficient for reproducibility; and
6. Aligning the strength of conclusions with the exploratory design and inference limits (including PICRUSt-based functional predictions).

The study is thoughtfully designed in several respects, including the paired gut/oral sampling, the focus on a family-based cohort, and the use of multiple reference databases. With the revisions below, the manuscript would be substantially stronger in technical validity and interpretability.

Major comments

1) Age and site imbalance (key confounding)
Microbiome composition is strongly influenced by age and by environmental/site factors. The current group structure appears meaningfully imbalanced by age and collection site, and these variables could plausibly explain a non-trivial portion of the observed separation. Please add sensitivity analyses that directly test robustness to these factors.
At minimum, I recommend considering:

• Stratified analyses, e.g., adult-only and child-only subsets where feasible;
• Models that include age and site as covariates (even if power is limited, reporting effect direction and magnitude is informative); and/or
• An approach to reduce imbalance, such as matched subsets or excluding the most extreme strata.
If these analyses are not feasible, the manuscript should substantially soften phenotype-linked interpretations and emphasize that findings are cohort-specific, exploratory signals.**

We thank the reviewer for this important methodological point. We agree that age and geographic site are potential confounding factors in microbiome studies and have taken steps to address this directly.

We conducted analyses examining the influence of both age and collection site on microbiome composition. With respect to geographic site, we did observe that it may represent a meaningful source of variation in our dataset. We have therefore added explicit acknowledgment of this throughout the manuscript, noting that geographic differences in diet, environment, and household exposures could contribute to the observed microbiome patterns. With respect to age, we similarly examined its potential influence on the observed group-level differences.

Given the modest sample size of this cohort, fully stratified analyses (e.g., adult-only and child-only subsets) were not feasible. We have therefore substantially softened phenotype-linked interpretations throughout the manuscript and consistently frame all findings as cohort-specific, exploratory signals that require replication in larger, more balanced cohorts before any generalizable conclusions can be drawn.

We have also expanded the Limitations section (L409-423) to explicitly discuss age and geographic site as potential confounders and to note that future studies should incorporate larger, geographically diverse samples with balanced group structures to disentangle disorder-specific microbiome signals from environmental and demographic influences.

**2) Family-based clustering (non-independence)

Because participants are related and may share household/environment, observations are not independent. For PERMANOVA and differential abundance analyses, the family/household structure should be incorporated explicitly for example:
• Restricted permutations/stratified permutations for PERMANOVA; and
• Where feasible, mixed-effects or otherwise cluster-robust approaches for differential abundance.
If mixed-effects modeling is not feasible due to sample size, a practical alternative is to report sensitivity checks such as leave-one-family-out analyses (or excluding influential family clusters) to demonstrate that key signals are not driven by a small number of families. In all cases, please include a clear statement describing pseudo-replication risk and how it was mitigated.**

We thank the reviewer for raising this important methodological concern. We fully acknowledge that the family-based structure of this cohort introduces non-independence among observations, and that this represents a meaningful limitation of the current analyses.

We agree that restricted permutations in PERMANOVA and mixed-effects modeling for differential abundance analyses would be the ideal approaches to account for family/household clustering. However, given the modest sample size of this cohort, mixed-effects modeling was not statistically feasible, as the number of parameters required would exceed what the data can reliably support.

We also note that in this cohort, geographic site and phenotype were substantially overlapping, meaning that families with a given diagnosis tended to cluster by location. This collinearity makes it difficult to fully disentangle the contributions of family structure, household environment, and phenotype to the observed microbiome patterns, and we acknowledge this explicitly as a limitation.

We consistently emphasize throughout the manuscript that all findings should be interpreted as exploratory and cohort-specific, and that replication in larger, independent family-based cohorts with sufficient sample sizes to support cluster-robust modeling will be essential to validate these signals.

**3) Species-level reporting from 16S V4 and database dependence

Species-level assignments from 16S V4 can be unstable and highly database-dependent. Since the manuscript already indicates differences across reference databases, I recommend making genus-level (or higher) interpretations the primary biological narrative, and clearly labeling any species-level findings as exploratory.
Please also clarify classifier training for each database, including whether reference sequences were trimmed to the exact amplicon region used in this study. If species-level highlights are retained, avoid diagnostic or causal phrasing, and consider explicitly noting that confirmation would require shotgun metagenomics and/or targeted qPCR in future work.**

We thank the reviewer for this important point regarding the limitations of species-level taxonomic assignments from 16S V4 data. We have revised the manuscript to include genus-level taxonomic patterns and to clearly label species-level findings as exploratory.

We have also clarified classifier training procedures in the Methods section (L156-164). Specifically, pre-trained Naive Bayes classifiers were used for each reference database, and reference sequences were not trimmed to the exact V4 amplicon region prior to classification. We now explicitly note that species-level assignments may be database-dependent and require confirmation using higher-resolution approaches such as shotgun metagenomics and/or targeted qPCR.

These revisions improve transparency and align interpretation with the resolution of the data.

**4) Negative controls and contamination/QC reporting

Given the small cohort and multi-step wet-lab pipeline, transparent reporting of extraction blanks, PCR negatives, and contamination screening is essential. Please add a dedicated Methods paragraph specifying:
• Which negative controls were included (and at what stages);
• QC thresholds used; and
• How potential contaminants were assessed and handled.
If negative controls were not included, this should be stated prominently, and low-abundance taxa findings should be interpreted conservatively.**

We thank the reviewer for highlighting the importance of transparent contamination reporting (L148-150). We have added a dedicated Methods paragraph explicitly stating that extraction blanks and PCR-negative controls were not included in this study. We clarify that, as a result, formal contaminant identification approaches based on negative controls could not be applied, and that low-abundance and species-level taxa should be interpreted conservatively.

We have also expanded the Limitations section (L428-434) to discuss the implications of this omission and to emphasize that future studies should include negative controls at multiple stages of the wet-lab pipeline. These revisions improve transparency and align interpretation with the technical constraints of the dataset.

**5) Data availability and reproducible metadata

Sharing reads and code is a strong step. To fully support reproducibility, please ensure that a de-identified metadata table required to reproduce the primary analyses is accessible, including at minimum: age, sex, collection site, phenotype variables, and an anonymized family/household ID. If some metadata must remain controlled, please describe a formal access pathway with clear criteria and governance.**

We thank the reviewer for emphasizing the importance of reproducible metadata. During initial submission, the metadata table was not included in the review materials because it contained identifying information. We have since fully de-identified the dataset and now provide a metadata table as a supplementary file.

The shared metadata include age, sex, sample collection site (fecal or saliva), phenotype variables, and an anonymized family/household identifier for each individual included in the analysis. These data are sufficient to reproduce the primary analyses reported in the manuscript.  **6) Conclusions and strength of language

The Discussion should consistently match claim strength to the exploratory design and to the limitations imposed by confounding and clustering. Please avoid wording that implies diagnostic utility, therapeutic targeting, or causal directionality. For PICRUSt-based functional predictions, emphasize that these represent inferred functional potential rather than measured function, and add brief interpretive cautions (and any quality/coverage indicators you can report).**
We have revised the Conclusion to align claim strength with the exploratory design and study limitations, removing language implying diagnostic or therapeutic utility, emphasizing associative findings, and explicitly clarifying that PICRUSt-based results represent inferred functional potential rather than measured function.

**Minor comments

1. Please correct minor typographical issues and use consistent statistical notation (e.g., “q = …”). Ensure consistent formatting for taxonomic names (italics for genus/species) and consistent naming of software/tools.
2. Please explicitly state which feature table level was used for alpha diversity, beta diversity, and PERMANOVA (ASV-level vs taxonomically collapsed tables), as this choice can affect results.**

Regarding minor comment #1, we have carefully reviewed the manuscript to correct typographical errors, standardize statistical notation, ensure all genus and species names are italicized throughout, and apply consistent naming conventions for all software and tools.

Regarding minor comment #2, we have added explicit clarification to the Methods section specifying the feature table levels used for each analysis. Specifically, we now state that alpha diversity, beta diversity, and PERMANOVA analyses were conducted on taxonomically collapsed feature tables at the genus level (p=6) and species level (p=7) in QIIME2 (L185-187).  **Overall assessment

Overall, this work has promise as a hypothesis-generating contribution. Addressing the confounding structure, family clustering, and reporting clarifications above would substantially improve technical soundness and better align the presented evidence with the manuscript’s conclusions.**
