# Supplementary material for: Distinct gut and oral microbiome patterns associated with dyslexia in a family-based cohort: A preliminary exploratory study
Source: PLoS One. 2026 Jul 21;21(7):e0353463. doi: 10.1371/journal.pone.0353463 (PMC13387549; doi:10.1371/journal.pone.0353463)
Supplement: Peer review history

# Peer review: Decision Letter 0

**DOI:** 10.1371/journal.pone.0353463.r001

**Authors:** Shimaa Yousof

2 Feb 2026

PONE-D-25-57492

Distinct Gut and Oral Microbiome Patterns Associated with Dyslexia in a Family-Based Cohort

PLOS One

Dear Dr. Wright,

Thank you for submitting your manuscript to PLOS ONE. After careful consideration, we feel that it has merit but does not fully meet PLOS ONE’s publication criteria as it currently stands. Therefore, we invite you to submit a revised version of the manuscript that addresses the points raised during the review process.

Please submit your revised manuscript by Mar 19 2026 11:59PM. If you will need more time than this to complete your revisions, please reply to this message or contact the journal office at plosone@plos.org. When you're ready to submit your revision, log on to https://www.editorialmanager.com/pone/ and select the 'Submissions Needing Revision' folder to locate your manuscript file.

Please include the following items when submitting your revised manuscript:

- A letter that responds to each point raised by the academic editor and reviewer(s). You should upload this letter as a separate file labeled 'Response to Reviewers'.
- A marked-up copy of your manuscript that highlights changes made to the original version. You should upload this as a separate file labeled 'Revised Manuscript with Track Changes'.
- An unmarked version of your revised paper without tracked changes. You should upload this as a separate file labeled 'Manuscript'.

If you would like to make changes to your financial disclosure, please include your updated statement in your cover letter. Guidelines for resubmitting your figure files are available below the reviewer comments at the end of this letter.

If applicable, we recommend that you deposit your laboratory protocols in protocols.io to enhance the reproducibility of your results. Protocols.io assigns your protocol its own identifier (DOI) so that it can be cited independently in the future. For instructions see: https://journals.plos.org/plosone/s/submission-guidelines#loc-laboratory-protocols. Additionally, PLOS ONE offers an option for publishing peer-reviewed Lab Protocol articles, which describe protocols hosted on protocols.io. Read more information on sharing protocols at https://plos.org/protocols?utm_medium=editorial-email&utm_source=authorletters&utm_campaign=protocols.

We look forward to receiving your revised manuscript.

Kind regards,

Shimaa Mohammad Yousof, Msc, M.D., Ph.D

Academic Editor

PLOS One

Journal Requirements:

When submitting your revision, we need you to address these additional requirements.

1.Please ensure that your manuscript meets PLOS ONE's style requirements, including those for file naming. The PLOS ONE style templates can be found at

https://journals.plos.org/plosone/s/file?id=wjVg/PLOSOne_formatting_sample_main_body.pdf and

https://journals.plos.org/plosone/s/file?id=ba62/PLOSOne_formatting_sample_title_authors_affiliations.pdf

2. Please note that funding information should not appear in any section or other areas of your manuscript. We will only publish funding information present in the Funding Statement section of the online submission form. Please remove any funding-related text from the manuscript.

3. We note that the grant information you provided in the ‘Funding Information’ and ‘Financial Disclosure’ sections do not match.

When you resubmit, please ensure that you provide the correct grant numbers for the awards you received for your study in the ‘Funding Information’ section.

4. Thank you for stating the following financial disclosure:

“Microbiome analysis was supported by a seed grant awarded to B.P. from the Institute for Social Science Research (ISSR) at Arizona State University. The work of M. O. was supported by the National Institute of Diabetes and Digestive and Kidney Diseases of the National Institutes of Health under Award Number T32DK137525.”

Please state what role the funders took in the study.  If the funders had no role, please state: "The funders had no role in study design, data collection and analysis, decision to publish, or preparation of the manuscript."

If this statement is not correct you must amend it as needed.

Please include this amended Role of Funder statement in your cover letter; we will change the online submission form on your behalf.

5. When completing the data availability statement of the submission form, you indicated that you will make your data available on acceptance. We strongly recommend all authors decide on a data sharing plan before acceptance, as the process can be lengthy and hold up publication timelines. Please note that, though access restrictions are acceptable now, your entire data will need to be made freely accessible if your manuscript is accepted for publication. This policy applies to all data except where public deposition would breach compliance with the protocol approved by your research ethics board. If you are unable to adhere to our open data policy, please kindly revise your statement to explain your reasoning and we will seek the editor's input on an exemption. Please be assured that, once you have provided your new statement, the assessment of your exemption will not hold up the peer review process.

6. Your ethics statement should only appear in the Methods section of your manuscript. If your ethics statement is written in any section besides the Methods, please delete it from any other section.

7. We note that Figure 1 in your submission contain copyrighted images. All PLOS content is published under the Creative Commons Attribution License (CC BY 4.0), which means that the manuscript, images, and Supporting Information files will be freely available online, and any third party is permitted to access, download, copy, distribute, and use these materials in any way, even commercially, with proper attribution. For more information, see our copyright guidelines: http://journals.plos.org/plosone/s/licenses-and-copyright.

We require you to either (1) present written permission from the copyright holder to publish these figures specifically under the CC BY 4.0 license, or (2) remove the figures from your submission:

1. You may seek permission from the original copyright holder of Figure 1 to publish the content specifically under the CC BY 4.0 license.

We recommend that you contact the original copyright holder with the Content Permission Form (http://journals.plos.org/plosone/s/file?id=7c09/content-permission-form.pdf) and the following text:

“I request permission for the open-access journal PLOS ONE to publish XXX under the Creative Commons Attribution License (CCAL) CC BY 4.0 (http://creativecommons.org/licenses/by/4.0/). Please be aware that this license allows unrestricted use and distribution, even commercially, by third parties. Please reply and provide explicit written permission to publish XXX under a CC BY license and complete the attached form.”

Please upload the completed Content Permission Form or other proof of granted permissions as an "Other" file with your submission.

In the figure caption of the copyrighted figure, please include the following text: “Reprinted from [ref] under a CC BY license, with permission from [name of publisher], original copyright [original copyright year].”

2. If you are unable to obtain permission from the original copyright holder to publish these figures under the CC BY 4.0 license or if the copyright holder’s requirements are incompatible with the CC BY 4.0 license, please either i) remove the figure or ii) supply a replacement figure that complies with the CC BY 4.0 license. Please check copyright information on all replacement figures and update the figure caption with source information. If applicable, please specify in the figure caption text when a figure is similar but not identical to the original image and is therefore for illustrative purposes only.

8. Please remove your figures from within your manuscript file, leaving only the individual TIFF/EPS image files, uploaded separately. These will be automatically included in the reviewers’ PDF.

9. We note that there is identifying data in the Supporting Information file “SupplementalTables.xlsx”. Due to the inclusion of these potentially identifying data, we have removed this file from your file inventory. Prior to sharing human research participant data, authors should consult with an ethics committee to ensure data are shared in accordance with participant consent and all applicable local laws.

Data sharing should never compromise participant privacy. It is therefore not appropriate to publicly share personally identifiable data on human research participants. The following are examples of data that should not be shared:

-Name, initials, physical address

-Ages more specific than whole numbers

-Internet protocol (IP) address

-Specific dates (birth dates, death dates, examination dates, etc.)

-Contact information such as phone number or email address

-Location data

-ID numbers that seem specific (long numbers, include initials, titled “Hospital ID”) rather than random (small numbers in numerical order)

Data that are not directly identifying may also be inappropriate to share, as in combination they can become identifying. For example, data collected from a small group of participants, vulnerable populations, or private groups should not be shared if they involve indirect identifiers (such as sex, ethnicity, location, etc.) that may risk the identification of study participants.

Additional guidance on preparing raw data for publication can be found in our Data Policy (https://journals.plos.org/plosone/s/data-availability#loc-human-research-participant-data-and-other-sensitive-data) and in the following article: http://www.bmj.com/content/340/bmj.c181.long.

Please remove or anonymize all personal information (<specific identifying information in file to be removed>), ensure that the data shared are in accordance with participant consent, and re-upload a fully anonymized data set. Please note that spreadsheet columns with personal information must be removed and not hidden as all hidden columns will appear in the published file.

10. If the reviewer comments include a recommendation to cite specific previously published works, please review and evaluate these publications to determine whether they are relevant and should be cited. There is no requirement to cite these works unless the editor has indicated otherwise.

Additional Editor Comments:

General comments:

Kindly, remove the repetitive speech in the manuscript about the sample size and the limitations. Only include it in the limitation and conclusion section.

Abstract:

Kindly, rewrite the abstract making it structured.

The aim of the study needs to be clarified in the abstract

Introduction and rational

Some issues needs clarification in the introduction and rational section

Why the authors compared the dyslexia with the CAS?- Kindly clarify your rational

Methods:

Why the number of saliva samples is different from fecal samples? Have some patients refused to give fecal samples? Were some samples spoilt?- Kindly, clarify these points in the methods section

Did you get the saliva and fecal samples from the same patient?

The authors mentioned the categorization of patients including undetermined phenotyping and neurotypical. Do the authors mean that 18 individual have symptoms and the others did not have? Could you clarify this point more for the reader in the manuscript?

The definitions of phenotype, neurotypical and alpha and beta diversity is better to be included to make it easier for the reader to catch up the meanings.

The results:

The results contain parts that looks like discussion. The standard is to display the results with a short comment about the trend if needed to express the meaning of the results. After that, you can discuss the results in details and compare with others using supporting references. Therefore, kindly, rewrite the result section based on these recommendations and omit the repeated parts.

The authors mentioned the mechanistic pathway results. Kindly, mention the importance of this mechanistic pathway briefly in the introduction section and add a sentence that reveals that you will assess it in the corresponding part in the methods section.

Limitation and conclusion:

Thanks for including the limitations and discussing them explicitly.

Add the inter-sex variability and ethnic variability to the limitations

Make the conclusion more concise and avoid repetition. Although the study is novel and important, Avoid speech that is overestimating of the results importance.

[Note: HTML markup is below. Please do not edit.]

Reviewers' comments:

Reviewer's Responses to Questions

**Comments to the Author**

1. Is the manuscript technically sound, and do the data support the conclusions?

The manuscript must describe a technically sound piece of scientific research with data that supports the conclusions. Experiments must have been conducted rigorously, with appropriate controls, replication, and sample sizes. The conclusions must be drawn appropriately based on the data presented.

Reviewer #1: Partly

Reviewer #2: Partly

**********

2. Has the statistical analysis been performed appropriately and rigorously?

Reviewer #1: Yes

Reviewer #2: No

**********

3. Have the authors made all data underlying the findings in their manuscript fully available?

The PLOS Data policy requires authors to make all data underlying the findings described in their manuscript fully available without restriction, with rare exception (please refer to the Data Availability Statement in the manuscript PDF file). The data should be provided as part of the manuscript or its supporting information, or deposited to a public repository. For example, in addition to summary statistics, the data points behind means, medians and variance measures should be available. If there are restrictions on publicly sharing data—e.g. participant privacy or use of data from a third party—those must be specified.

Reviewer #1: Yes

Reviewer #2: No

**********

4. Is the manuscript presented in an intelligible fashion and written in standard English?

PLOS ONE does not copyedit accepted manuscripts, so the language in submitted articles must be clear, correct, and unambiguous. Any typographical or grammatical errors should be corrected at revision, so please note any specific errors here.

Reviewer #1: Yes

Reviewer #2: Yes

**********

5. Review Comments to the Author

Please use the space provided to explain your answers to the questions above. You may also include additional comments for the author, including concerns about dual publication, research ethics, or publication ethics. (Please upload your review as an attachment if it exceeds 20,000 characters)

Reviewer #1: Major Comments

1. Clarify the Study Design and Cohort Composition

The abstract mentions a “family-based cohort,” but the relationships between dyslexia, CAS, and neurotypical participants are not explicitly stated.

Suggestion: Specify how many individuals belonged to each group and whether participants with dyslexia and CAS were from the same families or separate families.

2. Distinguish Between Dyslexia and CAS Groups More Clearly

The study groups appear uneven (fecal n=19, saliva n=29), making it unclear how many samples belonged to each diagnostic category.

Suggestion: Provide a brief breakdown (e.g., “X dyslexia, Y CAS, Z neurotypical”).

3. Interpretation of Findings Needs More Caution

The enrichment of Alistipes A 871400 communis is interesting, but the text risks implying a causal or mechanistic link.

Suggestion: Reinforce that the associations are correlative and the sample size is small.

4. More Detail About the Analytical Methods

Many tools and databases are listed, but the abstract does not explain why multiple databases were used or whether consistency across them was evaluated.

Suggestion: Briefly mention whether the enrichment or diversity patterns were robust across databases.

5. Clarify the Saliva vs. Fecal Microbiome Findings

The abstract notes differences in fecal microbiome profiles but is vague regarding saliva findings.

Suggestion: Clarify whether saliva microbiomes showed group-level differences or not.

Minor Comments

6. Precision in Terminology

“Participants with CAS histories” is unclear—does this mean persistent CAS, resolved CAS, or a history of speech delay?

Suggestion: Define the diagnostic category more precisely.

7. Improve Flow and Readability

The abstract is dense with methods, abbreviations, and technical references.

Suggestion: Streamline by focusing on the most important methodological points and moving details (e.g., specific database names) to methods in the full paper.

8. Grammar / Wording Adjustments

“remain underexplored” → “remains underexplored” (subject: microbiome).

“patterns compared to neurotypical family members and participants with CAS histories” — the comparison could be clearer if rephrased as two explicit comparisons.

9. Consider Briefly Describing Functional Results

PICRUSt2 analysis is mentioned, but no functional findings are described.

Suggestion: Add 1–2 words on whether functional predictions aligned with taxonomic differences.

Reviewer #2: Thank you for the opportunity to review this manuscript.

I have reviewed the manuscript entitled “Distinct Gut and Oral Microbiome Patterns Associated with Dyslexia in a Family-Based Cohort.” This is an exploratory study that addresses an interesting question using paired fecal and saliva 16S rRNA (V4) data. The overall concept is timely, and the hypothesis-generating framing is appropriate. However, for the work to be publishable as a technically robust exploratory report, several sensitivity analyses and methodological clarifications are needed primarily because (i) there appears to be substantial imbalance in age and collection site across comparison groups, and (ii) the family-based sampling introduces non-independence that must be accounted for in inferential analyses.

In my view, the key requirements are:

1. Explicitly addressing age/site confounding;

2. Accounting for clustering at the family/household level;

3. Tempering species-level interpretations from 16S V4 and clarifying classifier training;

4. Expanding reporting on negative controls/contamination and QC;

5. Ensuring metadata availability is sufficient for reproducibility; and

6. Aligning the strength of conclusions with the exploratory design and inference limits (including PICRUSt-based functional predictions).

The study is thoughtfully designed in several respects, including the paired gut/oral sampling, the focus on a family-based cohort, and the use of multiple reference databases. With the revisions below, the manuscript would be substantially stronger in technical validity and interpretability.

Major comments

1) Age and site imbalance (key confounding)

Microbiome composition is strongly influenced by age and by environmental/site factors. The current group structure appears meaningfully imbalanced by age and collection site, and these variables could plausibly explain a non-trivial portion of the observed separation. Please add sensitivity analyses that directly test robustness to these factors.

At minimum, I recommend considering:

• Stratified analyses, e.g., adult-only and child-only subsets where feasible;

• Models that include age and site as covariates (even if power is limited, reporting effect direction and magnitude is informative); and/or

• An approach to reduce imbalance, such as matched subsets or excluding the most extreme strata.

If these analyses are not feasible, the manuscript should substantially soften phenotype-linked interpretations and emphasize that findings are cohort-specific, exploratory signals.

2) Family-based clustering (non-independence)

Because participants are related and may share household/environment, observations are not independent. For PERMANOVA and differential abundance analyses, the family/household structure should be incorporated explicitly for example:

• Restricted permutations/stratified permutations for PERMANOVA; and

• Where feasible, mixed-effects or otherwise cluster-robust approaches for differential abundance.

If mixed-effects modeling is not feasible due to sample size, a practical alternative is to report sensitivity checks such as leave-one-family-out analyses (or excluding influential family clusters) to demonstrate that key signals are not driven by a small number of families. In all cases, please include a clear statement describing pseudo-replication risk and how it was mitigated.

3) Species-level reporting from 16S V4 and database dependence

Species-level assignments from 16S V4 can be unstable and highly database-dependent. Since the manuscript already indicates differences across reference databases, I recommend making genus-level (or higher) interpretations the primary biological narrative, and clearly labeling any species-level findings as exploratory.

Please also clarify classifier training for each database, including whether reference sequences were trimmed to the exact amplicon region used in this study. If species-level highlights are retained, avoid diagnostic or causal phrasing, and consider explicitly noting that confirmation would require shotgun metagenomics and/or targeted qPCR in future work.

4) Negative controls and contamination/QC reporting

Given the small cohort and multi-step wet-lab pipeline, transparent reporting of extraction blanks, PCR negatives, and contamination screening is essential. Please add a dedicated Methods paragraph specifying:

• Which negative controls were included (and at what stages);

• QC thresholds used; and

• How potential contaminants were assessed and handled.

If negative controls were not included, this should be stated prominently, and low-abundance taxa findings should be interpreted conservatively.

5) Data availability and reproducible metadata

Sharing reads and code is a strong step. To fully support reproducibility, please ensure that a de-identified metadata table required to reproduce the primary analyses is accessible, including at minimum: age, sex, collection site, phenotype variables, and an anonymized family/household ID. If some metadata must remain controlled, please describe a formal access pathway with clear criteria and governance.

6) Conclusions and strength of language

The Discussion should consistently match claim strength to the exploratory design and to the limitations imposed by confounding and clustering. Please avoid wording that implies diagnostic utility, therapeutic targeting, or causal directionality. For PICRUSt-based functional predictions, emphasize that these represent inferred functional potential rather than measured function, and add brief interpretive cautions (and any quality/coverage indicators you can report).

Minor comments

1. Please correct minor typographical issues and use consistent statistical notation (e.g., “q = …”). Ensure consistent formatting for taxonomic names (italics for genus/species) and consistent naming of software/tools.

2. Please explicitly state which feature table level was used for alpha diversity, beta diversity, and PERMANOVA (ASV-level vs taxonomically collapsed tables), as this choice can affect results.

Overall assessment

Overall, this work has promise as a hypothesis-generating contribution. Addressing the confounding structure, family clustering, and reporting clarifications above would substantially improve technical soundness and better align the presented evidence with the manuscript’s conclusions.

**********

6. PLOS authors have the option to publish the peer review history of their article (what does this mean?). If published, this will include your full peer review and any attached files.

If you choose “no”, your identity will remain anonymous but your review may still be made public.

**Do you want your identity to be public for this peer review?**  For information about this choice, including consent withdrawal, please see our Privacy Policy.

Reviewer #1: **Yes:** ahmed alshewered

Reviewer #2: No

**********

[NOTE: If reviewer comments were submitted as an attachment file, they will be attached to this email and accessible via the submission site. Please log into your account, locate the manuscript record, and check for the action link "View Attachments". If this link does not appear, there are no attachment files.]

To ensure your figures meet our technical requirements, please review our figure guidelines: https://journals.plos.org/plosone/s/figures

You may also use PLOS’s free figure tool, NAAS, to help you prepare publication quality figures: https://journals.plos.org/plosone/s/figures#loc-tools-for-figure-preparation.

NAAS will assess whether your figures meet our technical requirements by comparing each figure against our figure specifications.

# Author comment: Author response to Decision Letter 1

**DOI:** 10.1371/journal.pone.0353463.r002

19 Mar 2026

Reply to the Editor and Reviewers

We thank the Editor and reviewers for their thoughtful comments and constructive comments, which have greatly improved the clarity and rigor of our manuscript. We have carefully considered and addressed all comments point by point. Our detailed responses are provided below in red, and corresponding revisions have been made in the manuscript.

When submitting your revision, we need you to address these additional requirements.

1.Please ensure that your manuscript meets PLOS ONE's style requirements, including those for file naming. The PLOS ONE style templates can be found at

https://journals.plos.org/plosone/s/file?id=wjVg/PLOSOne_formatting_sample_main_body.pdf and

https://journals.plos.org/plosone/s/file?id=ba62/PLOSOne_formatting_sample_title_authors_affiliations.pdf

2. Please note that funding information should not appear in any section or other areas of your manuscript. We will only publish funding information present in the Funding Statement section of the online submission form. Please remove any funding-related text from the manuscript.

3. We note that the grant information you provided in the ‘Funding Information’ and ‘Financial Disclosure’ sections do not match.

When you resubmit, please ensure that you provide the correct grant numbers for the awards you received for your study in the ‘Funding Information’ section.

Thank you for bringing this to our attention. We have made sure the grant numbers are reported.

4. Thank you for stating the following financial disclosure:

“Microbiome analysis was supported by a seed grant awarded to B.P. from the Institute for Social Science Research (ISSR) at Arizona State University. The work of M. O. was supported by the National Institute of Diabetes and Digestive and Kidney Diseases of the National Institutes of Health under Award Number T32DK137525.”

Please state what role the funders took in the study. If the funders had no role, please state: "The funders had no role in study design, data collection and analysis, decision to publish, or preparation of the manuscript."

Thank you for this clarification. We have updated the text to address this concern.

5. When completing the data availability statement of the submission form, you indicated that you will make your data available on acceptance. We strongly recommend all authors decide on a data sharing plan before acceptance, as the process can be lengthy and hold up publication timelines. Please note that, though access restrictions are acceptable now, your entire data will need to be made freely accessible if your manuscript is accepted for publication. This policy applies to all data except where public deposition would breach compliance with the protocol approved by your research ethics board. If you are unable to adhere to our open data policy, please kindly revise your statement to explain your reasoning and we will seek the editor's input on an exemption. Please be assured that, once you have provided your new statement, the assessment of your exemption will not hold up the peer review process.

We have revised our data availability statement to state that our data is publicly available. Specifically, we state that all our data is on the NCBI SRA database, under the project number: PRJNA1246995. The SRA database is a commonly used repository for microbiome data to be deposited. We also provide our bioinformatic scripts on GitHub for reproducibility.

6. Your ethics statement should only appear in the Methods section of your manuscript. If your ethics statement is written in any section besides the Methods, please delete it from any other section.

We have ensured that the ethics statement is only in the Methods section (Lines 80-81).

7. We note that Figure 1 in your submission contain copyrighted images. All PLOS content is published under the Creative Commons Attribution License (CC BY 4.0), which means that the manuscript, images, and Supporting Information files will be freely available online, and any third party is permitted to access, download, copy, distribute, and use these materials in any way, even commercially, with proper attribution. For more information, see our copyright guidelines: http://journals.plos.org/plosone/s/licenses-and-copyright.

We require you to either (1) present written permission from the copyright holder to publish these figures specifically under the CC BY 4.0 license, or (2) remove the figures from your submission:

1. You may seek permission from the original copyright holder of Figure 1 to publish the content specifically under the CC BY 4.0 license.

We recommend that you contact the original copyright holder with the Content Permission Form (http://journals.plos.org/plosone/s/file?id=7c09/content-permission-form.pdf) and the following text:

“I request permission for the open-access journal PLOS ONE to publish XXX under the Creative Commons Attribution License (CCAL) CC BY 4.0 (http://creativecommons.org/licenses/by/4.0/). Please be aware that this license allows unrestricted use and distribution, even commercially, by third parties. Please reply and provide explicit written permission to publish XXX under a CC BY license and complete the attached form.”

Please upload the completed Content Permission Form or other proof of granted permissions as an "Other" file with your submission.

In the figure caption of the copyrighted figure, please include the following text: “Reprinted from [ref] under a CC BY license, with permission from [name of publisher], original copyright [original copyright year].”

Thank you for bringing this to our attention. To address this concern, we have updated our Figure 1 to ensure that it has no copyright images (i.e., we have removed all figures that were downloaded from BioRender). The updated figure also provides a clearer picture about the study design.

2. If you are unable to obtain permission from the original copyright holder to publish these figures under the CC BY 4.0 license or if the copyright holder’s requirements are incompatible with the CC BY 4.0 license, please either i) remove the figure or ii) supply a replacement figure that complies with the CC BY 4.0 license. Please check copyright information on all replacement figures and update the figure caption with source information. If applicable, please specify in the figure caption text when a figure is similar but not identical to the original image and is therefore for illustrative purposes only.

8. Please remove your figures from within your manuscript file, leaving only the individual TIFF/EPS image files, uploaded separately. These will be automatically included in the reviewers’ PDF.

Thank you for this comment. We have removed the figures from the manuscript.

9. We note that there is identifying data in the Supporting Information file “SupplementalTables.xlsx”. Due to the inclusion of these potentially identifying data, we have removed this file from your file inventory. Prior to sharing human research participant data, authors should consult with an ethics committee to ensure data are shared in accordance with participant consent and all applicable local laws.

Data sharing should never compromise participant privacy. It is therefore not appropriate to publicly share personally identifiable data on human research participants. The following are examples of data that should not be shared:

-Name, initials, physical address

-Ages more specific than whole numbers

-Internet protocol (IP) address

-Specific dates (birth dates, death dates, examination dates, etc.)

-Contact information such as phone number or email address

-Location data

-ID numbers that seem specific (long numbers, include initials, titled “Hospital ID”) rather than random (small numbers in numerical order)

Data that are not directly identifying may also be inappropriate to share, as in combination they can become identifying. For example, data collected from a small group of participants, vulnerable populations, or private groups should not be shared if they involve indirect identifiers (such as sex, ethnicity, location, etc.) that may risk the identification of study participants.

Additional guidance on preparing raw data for publication can be found in our Data Policy (https://journals.plos.org/plosone/s/data-availability#loc-human-research-participant-data-and-other-sensitive-data) and in the following article: http://www.bmj.com/content/340/bmj.c181.long.

Please remove or anonymize all personal information (<specific identifying information in file to be removed>), ensure that the data shared are in accordance with participant consent, and re-upload a fully anonymized data set. Please note that spreadsheet columns with personal information must be removed and not hidden as all hidden columns will appear in the published file.

Thank you for bringing this to our attention. We have removed all potentially identifying data. All metadata columns (i.e., anonymized SampleID, Family_ID, SampleType, Gender, Ethnicity, Phenotype, and Relationship to Proband) have been approved by the IRB.

10. If the reviewer comments include a recommendation to cite specific previously published works, please review and evaluate these publications to determine whether they are relevant and should be cited. There is no requirement to cite these works unless the editor has indicated otherwise.

Additional Editor Comments:

General comments:

Kindly, remove the repetitive speech in the manuscript about the sample size and the limitations. Only include it in the limitation and conclusion section.

We thank the editor for this suggestion. We have revised the manuscript to remove repetitive references to sample size and study limitations from the Results section. These considerations are now discussed exclusively in the Discussion and reiterated in the Conclusion to ensure clarity without redundancy.

Abstract:

Kindly, rewrite the abstract making it structured.

The aim of the study needs to be clarified in the abstract

We have rewritten the abstract in a structured format and explicitly clarified the aim of the study, stating that the objective was to determine whether fecal and saliva microbiome features are associated with dyslexia or childhood apraxia of speech (CAS).

Introduction and rational

Some issues needs clarification in the introduction and rational section

Why the authors compared the dyslexia with the CAS?- Kindly clarify your rational

We thank the editor for this question. In the Introduction, we added the following explanation for selecting CAS and dyslexia for this study: “[6,7]. Although each [CAS and dyslexia] is distinct in their core observable characteristics (i.e., phenotypes) and clinical management, both are frequently comorbid [8] and share some biomarkers for motor discoordination and difficulties with sequential information processing [9].”

In addition, in the third paragraph of the Introduction, we added, “Yet, they [CAS and dyslexia] may share overlapping neurobiological vulnerabilities. This makes them a useful comparative framework for exploring biological factors that may be shared across language impairments versus those that are disorder specific. One such factor may be the microbiome, as emerging evidence indicates that microbial communities involved along the oral-gut-brain axis can influence neurodevelopment, cognition, and behavior through immune, metabolic, and neuroactive signaling pathways [21–23].”

In short, this comparative framework allows us to explore whether microbiome associations are disorder-specific or shared across language impairments within a family-based design.

Methods:

Why the number of saliva samples is different from fecal samples? Have some patients refused to give fecal samples? Were some samples spoilt?- Kindly, clarify these points in the methods section

We thank the reviewer for this clarification request. We added an explanation in the Methods section: “In total, 29 saliva samples and 19 fecal samples were collected. The difference in sample numbers reflect participant choice to provide both sample types or saliva only. Some individuals elected to provide saliva samples only due to convenience and easy of collection. For all 19 fecal samples, a matched saliva sample from the same participant was also available.”

Did you get the saliva and fecal samples from the same patient?

Yes. Everyone who provided a fecal sample also provided a saliva sample, consistent with the explanation we added for the difference in sample numbers.

The authors mentioned the categorization of patients including undetermined phenotyping and neurotypical. Do the authors mean that 18 individual have symptoms and the others did not have?

We thank the editor for this clarification request. Yes, this is correct. Table 1 shows the breakdown of participants with dyslexia (7), with CAS (11), neither (11), or unknown (1). We added this clarification to the text.

Could you clarify this point more for the reader in the manuscript?

The definitions of phenotype, neurotypical and alpha and beta diversity is better to be included to make it easier for the reader to catch up the meanings.

We have revised the manuscript to improve clarity for readers. Definitions of participant phenotype and neurotypical status have been added to the Introduction. Moreover, we introduced the term phenotype as observable characteristic. We added the term “unaffected” to the term “neurotypical” for clarity. The definitions and operationalization of alpha and beta diversity metrics have been added to the Methods section.

Results:

The results contain parts that looks like discussion. The standard is to display the results with a short comment about the trend if needed to express the meaning of the results. After that, you can discuss the results in details and compare with others using supporting references. Therefore, kindly, rewrite the result section based on these recommendations and omit the repeated parts.

We thank the editor for this helpful recommendation. We have revised the Results section to focus strictly on the presentation of findings, with only brief descriptive statements included where necessary to convey observed trends. Interpretive language, literature comparisons, and repeated discussion of limitations have been removed from the Results and consolidated into the Discussion section. Redundant content has also been removed to improve clarity and conciseness.

The authors mentioned the mechanistic pathway results. Kindly, mention the importance of this mechanistic pathway briefly in the introduction section and add a sentence that reveals that you will assess it in the corresponding part in the methods section.

We thank the reviewer for this helpful suggestion. We have revised the Introduction to briefly describe the relevance of microbial metabolic pathways implicated in neuroactive signaling and sulfur metabolism and to clarify their potential importance in the context of the oral-gut-brain axis (L65-67).

Limitation and conclusion:

Thanks for including the limitations and discussing them explicitly.

Add the inter-sex variability and ethnic variability to the limitations

Make the conclusion more concise and avoid repetition. Although the study is novel and important, Avoid speech that is overestimating of the results importance.

We thank the reviewer for this helpful feedback. We have revised the Limitations section to explicitly include inter-sex variability and the predominantly White composition of the cohort as additional factors that may limit generalizability.

We have also revised the Conclusion to improve concision, remove repetitive statements, and ensure that the strength of the language appropriately reflects the exploratory nature of the study. Specifically, we avoided wording that could be interpreted as overstating the importance or immediate implications of the findings, while retaining the novelty and relevance of the work.

Reviewers' comments:

Reviewer's Responses to Questions

Reviewer #1:

Major Comments

1. Clarify the Study Design and Cohort Composition

The abstract mentions a “family-based cohort,” but the relationships between dyslexia, CAS, and neurotypical participants are not explicitly stated.

Suggestion:

Attachment Submitted filename: Reply-to-the-Editor-and-Reviewers.docx

# Peer review: Decision Letter 1

**DOI:** 10.1371/journal.pone.0353463.r003

**Authors:** Shimaa Yousof

25 Jun 2026

Distinct gut and oral microbiome patterns associated with dyslexia in a family-based cohort

PONE-D-25-57492R1

Dear Dr. Wright,

Dear Authors

I am delighted to inform you that your article is provisionally accepted for publication.  However, minor corrections need to be done before publication.

-The title identifies the work as a preliminary exploratory study.Add this sentence to the title: "a preliminary exploratory study".

-The Abstract and Conclusion explicitly state that the findings are hypothesis-generating. Add this sentence to the abstract" "This preliminary exploratory study should be considered hypothesis-generating."

-The limitations section clearly emphasizes the unresolved issues of family clustering, site confounding, and lack of contamination controls. Add this sentence to the discussion: "Given the potential confounding by collection site, family clustering, and the absence of laboratory negative controls, these findings require validation in larger independent cohorts."

Within one week, you’ll receive an e-mail detailing the required amendments. When these have been addressed, you’ll receive a formal acceptance letter and your manuscript will be scheduled for publication.

An invoice will be generated when your article is formally accepted. Please note, if your institution has a publishing partnership with PLOS and your article meets the relevant criteria, all or part of your publication costs will be covered. Please make sure your user information is up-to-date by logging into Editorial Manager at Editorial Manager® and clicking the ‘Update My Information' link at the top of the page. For questions related to billing, please contact billing support.

If your institution or institutions have a press office, please notify them about your upcoming paper to help maximize its impact. If they’ll be preparing press materials, please inform our press team as soon as possible -- no later than 48 hours after receiving the formal acceptance. Your manuscript will remain under strict press embargo until 2 pm Eastern Time on the date of publication. For more information, please contact onepress@plos.org.

Kind regards,

Shimaa Mohammad Yousof, Msc, M.D., Ph.D

Academic Editor

PLOS One

Additional Editor Comments

Reviewers' comments:

Reviewer's Responses to Questions

**Comments to the Author**

1. If the authors have adequately addressed your comments raised in a previous round of review and you feel that this manuscript is now acceptable for publication, you may indicate that here to bypass the “Comments to the Author” section, enter your conflict of interest statement in the “Confidential to Editor” section, and submit your "Accept" recommendation.

Reviewer #3: (No Response)

Reviewer #4: All comments have been addressed

**********

2. Is the manuscript technically sound, and do the data support the conclusions?

The manuscript must describe a technically sound piece of scientific research with data that supports the conclusions. Experiments must have been conducted rigorously, with appropriate controls, replication, and sample sizes. The conclusions must be drawn appropriately based on the data presented.

Reviewer #3: Partly

Reviewer #4: Yes

**********

3. Has the statistical analysis been performed appropriately and rigorously?

Reviewer #3: Yes

Reviewer #4: Yes

**********

4. Have the authors made all data underlying the findings in their manuscript fully available?

The PLOS Data policy requires authors to make all data underlying the findings described in their manuscript fully available without restriction, with rare exception (please refer to the Data Availability Statement in the manuscript PDF file). The data should be provided as part of the manuscript or its supporting information, or deposited to a public repository. For example, in addition to summary statistics, the data points behind means, medians and variance measures should be available. If there are restrictions on publicly sharing data—e.g. participant privacy or use of data from a third party—those must be specified.

Reviewer #3: Yes

Reviewer #4: Yes

**********

5. Is the manuscript presented in an intelligible fashion and written in standard English?

PLOS ONE does not copyedit accepted manuscripts, so the language in submitted articles must be clear, correct, and unambiguous. Any typographical or grammatical errors should be corrected at revision, so please note any specific errors here.

Reviewer #3: Yes

Reviewer #4: Yes

**********

6. Review Comments to the Author

Please use the space provided to explain your answers to the questions above. You may also include additional comments for the author, including concerns about dual publication, research ethics, or publication ethics. (Please upload your review as an attachment if it exceeds 20,000 characters)

Reviewer #3: Thank you for the invitation to review the revision.

The authors have addressed several reporting and transparency concerns; however, important methodological limitations remain, particularly unresolved confounding by site/phenotype, unaccounted family-level clustering, absence of negative controls, and limited reproducibility of metadata. These should be explicitly reflected in the Results, Discussion, Limitations, Abstract, and Data Availability Statement before further consideration.

1. The authors state they examined age and collection site, and acknowledge geography as a meaningful source of variation, but they do not clearly report adjusted models, matched analyses, or stratified sensitivity analyses. Since phenotype and site appear to overlap strongly, this remains a major interpretive limitation.

2. Family/household clustering remains insufficiently handled. The original concern was non-independence due to related participants and shared household/environment. The authors acknowledge this and explain that mixed-effects modelling was not feasible. Still, they also did not provide restricted PERMANOVA, leave-one-family-out analyses, or cluster-robust sensitivity checks. This is probably the most important unresolved methodological issue.

3. Authors now state that no extraction blanks or PCR-negative controls were included. This is transparent, but it means formal contaminant detection could not be performed. For a small microbiome study, this substantially weakens confidence in low-abundance and species-level findings.

4. Data availability may still be incomplete. Authors say reads and scripts are available, but the data availability statement indicates that deidentified demographic information “can be requested,” rather than being fully publicly available. This may conflict with PLOS ONE’s preference for complete public availability unless an ethics-based restriction is justified.

5. Some wording still risks overstatement. Phrases such as “distinct faecal and saliva microbiomes” and “key taxa associated with dyslexia” are acceptable only if consistently framed as exploratory, cohort-specific, and non-causal. I would recommend replacing “key taxa” with “candidate taxa” or “taxa showing exploratory associations.”

Reviewer #4: Wright and colleagues investigated fecal and salivary microbiome diversity, composition, and functional potential using 16S rRNA gene amplicon sequencing to explore potential developmental and etiological links in childhood apraxia of speech (CAS) and dyslexia. The manuscript reports that individuals with dyslexia exhibit distinct fecal microbiome diversity patterns compared to those with CAS, along with dyslexia-associated functional pathways in the fecal microbiome, whereas no functional differences were observed in the salivary microbiome. In addition to genetic predisposition studies, this work provides an interesting perspective suggesting that microbial communities along the oral–gut–brain axis may be associated with dyslexia and CAS, potentially influencing neurodevelopment and neural activity.

However, as this is an exploratory study, the sample size is limited, which may lead to overinterpretation and should caution against inferring causality.

The manuscript appropriately acknowledges and addresses several key methodological concerns:

• The cohort composition has been clarified by explicitly describing family relationships, and the issue of non-independence due to family-based clustering has been acknowledged, with an emphasis on the need for replication in independent cohorts.

• The authors recognize that causal inference is not possible given the limited sample size and appropriately highlight the need for larger cohorts and more comprehensive analytical approaches in future studies.

• To avoid overinterpretation, the results have been appropriately reframed as correlational, and functional findings are clearly presented as predictive rather than causal.

Overall, the authors have thoughtfully addressed the major and minor concerns raised, improving the clarity, rigor, and interpretability of the manuscript while appropriately maintaining an exploratory and hypothesis-generating framework.

**********

7. PLOS authors have the option to publish the peer review history of their article (what does this mean?). If published, this will include your full peer review and any attached files.

If you choose “no”, your identity will remain anonymous but your review may still be made public.

**Do you want your identity to be public for this peer review?**  For information about this choice, including consent withdrawal, please see our Privacy Policy.

Reviewer #3: **Yes:** Sonu Bhaskar

Reviewer #4: No

**********

# Editor report: Acceptance letter

**DOI:** 10.1371/journal.pone.0353463.r004

**Authors:** Shimaa Yousof

PONE-D-25-57492R1

PLOS One

Dear Dr. Wright,

I'm pleased to inform you that your manuscript has been deemed suitable for publication in PLOS One. Congratulations! Your manuscript is now being handed over to our production team.

At this stage, our production department will prepare your paper for publication. This includes ensuring the following:

* All references, tables, and figures are properly cited

* All relevant supporting information is included in the manuscript submission,

* There are no issues that prevent the paper from being properly typeset

You will receive further instructions from the production team, including instructions on how to review your proof when it is ready. Please keep in mind that we are working through a large volume of accepted articles, so please give us a few days to review your paper and let you know the next and final steps.

Lastly, if your institution or institutions have a press office, please let them know about your upcoming paper now to help maximize its impact. If they'll be preparing press materials, please inform our press team within the next 48 hours. Your manuscript will remain under strict press embargo until 2 pm Eastern Time on the date of publication. For more information, please contact onepress@plos.org.

You will receive an invoice from PLOS for your publication fee after your manuscript has reached the completed accept phase. If you receive an email requesting payment before acceptance or for any other service, this may be a phishing scheme. Learn how to identify phishing emails and protect your accounts at https://explore.plos.org/phishing.

If we can help with anything else, please email us at customercare@plos.org.

Thank you for submitting your work to PLOS ONE and supporting open access.

Kind regards,

PLOS ONE Editorial Office Staff

on behalf of

Associate Professor Shimaa Mohammad Yousof

Academic Editor

PLOS One
